# Supplementary material for: Dementia care gaps in Sub-Saharan Africa: A scoping review of technology, environmental, community perspectives, and financial constraints
Source: PLOS Glob Public Health. 2026 Oct 6;6(10):e0007265. doi: 10.1371/journal.pgph.0007265 (PMC13641696; doi:10.1371/journal.pgph.0007265)
Supplement: Supplementary file 1 [file pgph.0007265.s003.pdf]

PLOS Global Public Health

Understanding Dementia Care Gaps in SSA: An Integrated Analysis of Technology, Social Determinants, Community Perspectives, and Financial Constraints

--Manuscript Draft--

Commented [J1]: Strong but slightly verbose; consider tightening by removing redundancy ("Integrated Analysis" already implies multidimensional scope).

**Manuscript Number:**	PGPH-D-26-00901
**Article Type:**	Research Article
**Full Title:**	Understanding Dementia Care Gaps in SSA: An Integrated Analysis of Technology, Social Determinants, Community Perspectives, and Financial Constraints
**Short Title:**	SSA Dementia Review
**Corresponding Author:**	Maiya G. Block Ngaybe<br>The University of Arizona College of Architecture Planning and Landscape Architecture<br>Tucson, AZ UNITED STATES OF AMERICA
**Order of Authors:**	Soarov Chakra Borty
	Maiya G. Block Ngaybe, PhD, MPH
	Angela E Apeagyei, PhD
	Divya Bhagianadh, PhD, MPH
	Monica H Swahn, PhD
	Nazmus Sakib, PhD, MS
**Keywords:**	Dementia; Aging; Global Health; Sub-Saharan Africa; Health systems
**Abstract:**	Dementia is an emerging public health problem in Sub-Saharan Africa (SSA). Rapidly changing demographics, underdeveloped health infrastructure, and limited awareness contribute to delayed diagnosis, treatment, and care. This review examined recent literature from 2021 to 2025 to identify gaps in dementia care across four key domains: environmental and social factors, technological capacity, community-based caregiving, and financial and economic factors. First, social and ecological factors, including low health literacy, rural residence, and weak primary care infrastructure, influence disease diagnosis and access to care. Second, technological capacity remains limited, with inadequate availability of diagnostic tools, telemedicine facilities, electronic health information systems, and digital health solutions that could improve early detection, management, and monitoring. Third, evidence from community-based care highlights heavy reliance on informal caregivers who often lack training and institutional support, resulting in substantial emotional, physical, and financial burden. Finally, inadequate public financing for dementia care, coupled with limited insurance coverage, further restricts access to dementia care services and support. Overall, this review highlights critical gaps in policy, evidence generation, cost-effective technology investment, caregiver support, and health systems strengthening across all levels of care. Addressing these multidimensional challenges is crucial to improving the quality of life for people living with dementia and their caregivers across SSA.
**Additional Information:**	
**Question**	**Response**
**Financial Disclosure**<br><br>Enter a financial disclosure statement that describes the sources of funding for the work included in this submission and the role the funder(s) played. This includes grants and any commercial funding of the work or authors.	No funding to disclose.

Powered by Editorial Manager® and ProduXion Manager® from Aries Systems Corporation

This statement will be typeset if the manuscript is accepted for publication.<br><br>*Please review the submission guidelines and the instructions link below for detailed requirements and guidance.*	
**Competing Interests**<br><br>On behalf of all authors, disclose any competing interests that could be perceived to bias this work.<br><br>This statement will be typeset if the manuscript is accepted for publication.<br><br>*Please review the instructions link below and PLOS Global Public Health's competing interests policy to determine what information must be disclosed at submission.*	None to disclose.
**Data Availability**<br><br>Before publication, Authors are required to make fully available and without restriction all data underlying their findings. Please see our PLOS Data Policy page for detailed information on this policy.<br><br>A **Data Availability Statement**, detailing where the data can be accessed, is required at first submission. Insert your Data Availability Statement in the box below.<br><br>Please see the data reporting section of our submission guidelines for instructions on what you need to include in your Data Availability Statement.	All data are publicly available as this is a review of peer-reviewed publications.

This statement will be typeset if the manuscript is accepted for publication.<br><br>*PLOS allows rare exemptions to address legal and ethical concerns. If you have legal or ethical restrictions, please use the box below to detail these in full sentences for the Journal team to consider.*	

Powered by Editorial Manager® and ProduXion Manager® from Aries Systems Corporation

Manuscript

Click here to access/download;Manuscript;PLOS_Global_Health_CUGH_Dem

Understanding Dementia Care Gaps in Sub-Saharan Africa: An Integrated Analysis of Social Determinants, Technological Capacity, Community Care, and Financial Constraints

Soarov Chakra Borty[1], Maiya G. Block Ngaybe[2], Angela E Apeagyei[3], Divya Bhagianadh[4], Monica H Swahn[5], Nazmus Sakib[1]

1 Department of Information Technology, Kennesaw State University, Kennesaw, Georgia, United States
2 School of Landscape Architecture, College of Architectural Planning and Landscape Architecture, University of Arizona, Tucson, Arizona, United States
3 Department of Health Metrics Sciences, School of Medicine, University of Washington, Seattle, Washington, United States
4 Department of Health, Human Performance, & Recreation, University of Arkansas, Fayetteville, Arkansas, United States
5 School of Public Health, Virginia Commonwealth University, Richmond, Virginia, United States

*mgblock@arizona.edu

Abstract

Dementia is an emerging public health problem in Sub-Saharan Africa (SSA). Rapidly changing demographics, underdeveloped health infrastructure, and limited awareness of dementia symptoms and care pathways contribute to delayed diagnosis, treatment, and care. This review examined recent literature from 2021 to 2025 to identify gaps in dementia care across four key domains: environmental and social factors, technological capacity, community-based caregiving, and financial and economic factors. First, social and ecological factors, including low health literacy, rural residence, and weak primary care infrastructure, influence disease diagnosis and access to care. Second, technological capacity remains limited, with inadequate availability of diagnostic tools, telemedicine facilities, electronic health information systems, and digital health solutions that could improve early detection, management, and monitoring. Third, evidence from community-based care highlights heavy reliance on informal caregivers who often lack training and institutional support, resulting in substantial emotional, physical, and financial burden. Finally, inadequate public financing for dementia care, coupled with limited insurance coverage, further restricts access to dementia care services and support. Overall, this review highlights critical gaps in policy, evidence generation, cost-effective technology investment, caregiver support, and health systems strengthening across all levels of care. Addressing these multidimensional challenges is crucial to improving the quality of life for people living with dementia and their caregivers across SSA.

Commented [J2]: Generic phrasing; please quantify burden (e.g., prevalence or growth rate) for stronger impact.

Commented [J3]: This is a good temporal scope; but please clarify whether this is a **systematic or scoping review** explicitly here

Commented [J4]: This sentence needs at least one concrete example (e.g., MRI scarcity, telemedicine coverage).

1 Introduction

Dementia is an emerging public health concern in the SSA region due to epidemiological and demographic transitions, as well as significant inequities in access to diagnostic services and long-term care services [1–6]. By 2050, people living with dementia

Commented [J5]: Redundant phrasing ("Sub-Saharan Africa (SSA)" already defined).

(PLWD) will double in SSA, although they currently account for a smaller share of global dementia cases than in high-income countries [7–9]. This relatively low reported case count is likely due to large numbers of missed diagnoses and limited capabilities. Dementia in SSA remains broadly overlooked across international research, surveillance efforts, and policy agendas, despite these trends [10, 11]. Caregiving for PLWD is almost entirely the responsibility of informal caregivers (primarily women) in these settings, as in other low and middle-income countries (LMIC). Resource constraints and minimal healthcare infrastructure exacerbate the burden of dementia and limit access to formal care services. While health expenditure remains generally low at the national level across all LMICs, SSA bears the lowest health spending of any region in the world. In 2021, countries in SSA spent an estimated USD 92 per capita on health, compared with USD 379 per capita in North Africa and the Middle East, which ranked next lowest at the time [12]. This disparity partly explains the strong reliance on informal caregivers seen in LMIC settings. Unpaid informal caregiving (65% of total costs) is the most significant contributor to the overall financial burden of dementia in LMICs, and most countries fail to offer any support to informal carers [11].

Over the past three or four decades, SSA African countries have witnessed a remarkable increase in life expectancy, with a focus on maternal and child health, infectious disease control, and primary health care [13]. As a result, the phenomenon of population aging has appeared more quickly than in previous generations, which has brought attention to the problem of dementia and other age-related health issues. In this regard, the problem of dementia and research on aging can be considered a relatively recent phenomenon, which has brought a timely opportunity to develop and expand models of dementia care and research tailored to the aging population of SSA. Dementia in sub-Saharan Africa has historically received limited attention within health systems and policy agendas, though recent national and international efforts point to increasing engagement with dementia-related challenges. Several studies from various countries, including Ethiopia, Ghana, Kenya, Nigeria, South Africa, and Tanzania, have been conducted in recent years [14–18]. These studies emphasize the impact of environmental circumstances, social inequities, culture, and fragile health systems on shaping dementia care facilities. Moreover, these analyses highlight gaps in policy, a lack of health insurance, limited diagnostic capabilities, and a shortage of specialized healthcare professionals. Several factors, including high medical expenses and inadequate technological support for dementia care-seeking, place a significant financial burden on households in SSA [19]. Families often face numerous challenges, including out-of-pocket costs, time spent on healthcare visits, transportation costs, assistive services availability, loss of leisure time, and psychosocial strain among caregivers [17, 20]. Coupled with limited health insurance coverage and weak social welfare systems, these pressures often lead to catastrophic health expenditures [18, 21], where households spend more than 10% of their household income on health care. Despite their importance, comprehensive and systematically quantified assessments of these technological, financial, and economic burdens remain rare in SSA.

Dementia care in SSA is characterized by intersecting challenges in research capacity, workforce development, and policy environments. This review examines the existing evidence on technological gaps, financial and economic barriers, and preliminary obstacles to early diagnosis in SSA. Comprehensive dementia care encompasses numerous layers of the healthcare system, including technology, workforce capacity, community support, and financial protection. The four classifications map directly onto these layers, permitting gaps to be examined across prevention, diagnosis, care delivery, and long-term support. Together, these classifications are utilized as a systems-based organizing structure to frame the gap analysis, consistent with the WHO health system framework [22]. This systems-based structure supports cross-domain integration. This

Commented [J6]: Please provide exact projected figures for precision

Commented [J7]: Vague; specify *diagnostic capacity constraints*.

Commented [J8]: Strong data point; include citation consistency.

Commented [J9]: Informal phrasing; replace with exact timeframe.

Commented [J10]: Avoid subjective language; please quantify change.

Commented [J11]: Needs citation or clearer justification.

Commented [J12]: Weak phrasing; specify number or type of studies. ...

Commented [J13]: Broad claim; you need to supporting reference or clarification

Commented [J14]: Strong claim; but must be supported by citation.

Commented [J15]: Ambiguous; clarify whether structural, clinical, or policy-related.

Commented [J16]: I think these should be explicitly listed again for clarity

review focuses on studies/papers published between 2021 and 2025; earlier landmark analyses and global reports are cited to provide epidemiological context and conceptual framing.

In line with the World Health Organization (WHO) health system building blocks framework that views healthcare systems through the lens of six interrelated components: service delivery, health workforce, health information systems, medical products and technologies, financing, and leadership and governance, this review takes on the systems-based approach to provide insights on gaps in dementia care provision in SSA [22, 23]. This paper adds to the literature by providing a comprehensive review across multiple domains relevant to dementia care in SSA, with a focus on more recent studies. This review synthesizes the existing literature to identify research gaps and explore reported health care technologies to address issues across various aspects of the health system, including delayed diagnosis, inadequate care coordination, and family-based caregiving.

2 Methods

2.1 Study Design and Reporting Framework

The study was undertaken as a scoping review to systematically present the available literature gaps in dementia care for PLWD in SSA. The scoping review approach was chosen because of the diversity and evolving nature of research on dementia care in SSA, which has involved studies ranging from clinical trials to healthcare system research, policy studies, and technology-based interventions. The review sought to determine the extent of the available recent literature, the major themes, and the gaps in the research and care. The review process followed the guidelines outlined in the extension of the Preferred Reporting Items for Systematic Reviews and Meta-Analysis (PRISMA-ScR), which provides a framework to ensure transparency, reproducibility, and quality [24].

Commented [J17]: Good choice; however, justification should explicitly reference methodological literature

Commented [J18]: Acceptable but could be tightened for concision.

2.2 Information Sources and Search Strategy

An extensive literature search was conducted using various online databases, including PubMed, Scopus, ScienceDirect, IEEE Xplore, and Google Scholar. These databases were chosen to cover the various disciplines of dementia care in SSA, including biomedical, public health, social sciences, and technology-based research. The literature search was limited to the period from January 2021 to December 2025. This time frame was chosen to cover the latest literature on dementia care, healthcare, and technology-based interventions in SSA. In addition to the above databases, the literature search was supplemented by a website search and citation tracking of key articles. The literature search was conducted using combinations of keywords related to dementia and SSA, including "dementia," "Alzheimer's disease," "cognitive impairment," "aging," "care," "caregiving," "health systems," "healthcare access," "technology," "digital health," and "SSA."

Commented [J19]: Specify search strings or Boolean operators for reproducibility.

Commented [J20]: Needs justification due to reproducibility concerns.

Commented [J21]: Too vague; specify which websites

2.3 Eligibility Criteria

Commented [J22]: Formatting inconsistent (bullet indentation irregular).

The studies were selected based on predetermined inclusion and exclusion criteria.

Inclusion criteria:

i. Studies related to dementia, Alzheimer's, and other cognitive impairments.

ii. Studies conducted in, or relevant to, countries within SSA region.

iii. Studies relevant to dementia care, caregiving, healthcare access, workforce issues, and policy and system or technology-related interventions.

iv. Original research articles, review articles, and studies published within the period 2021-2025.

Exclusion criteria:

i. Studies not relevant to dementia or cognitive impairments.

ii. Studies not relevant to SSA region.

iii. Studies not relevant to the scope of dementia care and healthcare system issues.

iv. Editorials, commentaries, or opinion articles without substantive empirical or analytical content.

Commented [J23]: Good, but clarify whether systematic reviews were included.

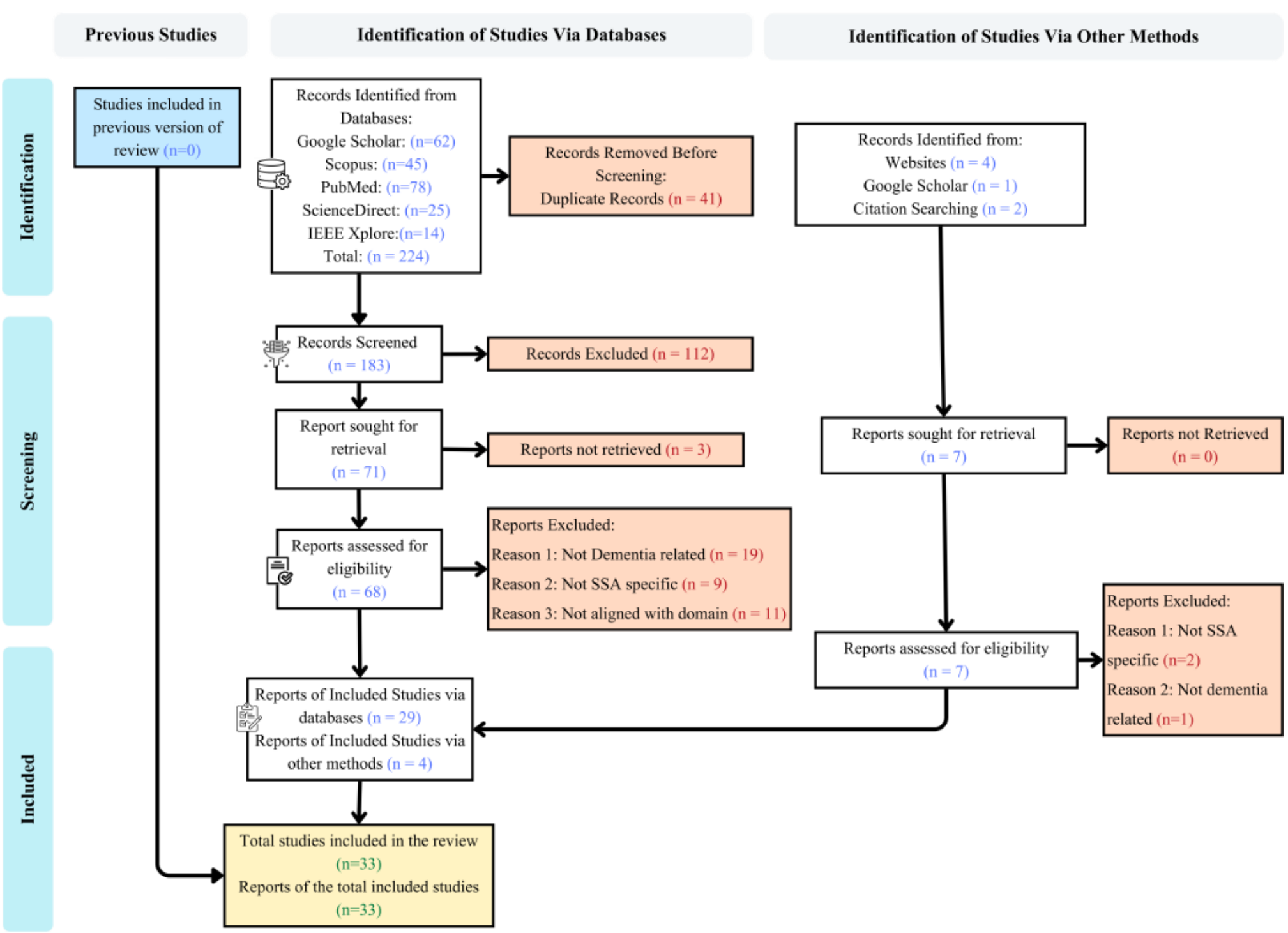

Fig 1. Flow diagram of literature search and screening for dementia care gaps in SSA.

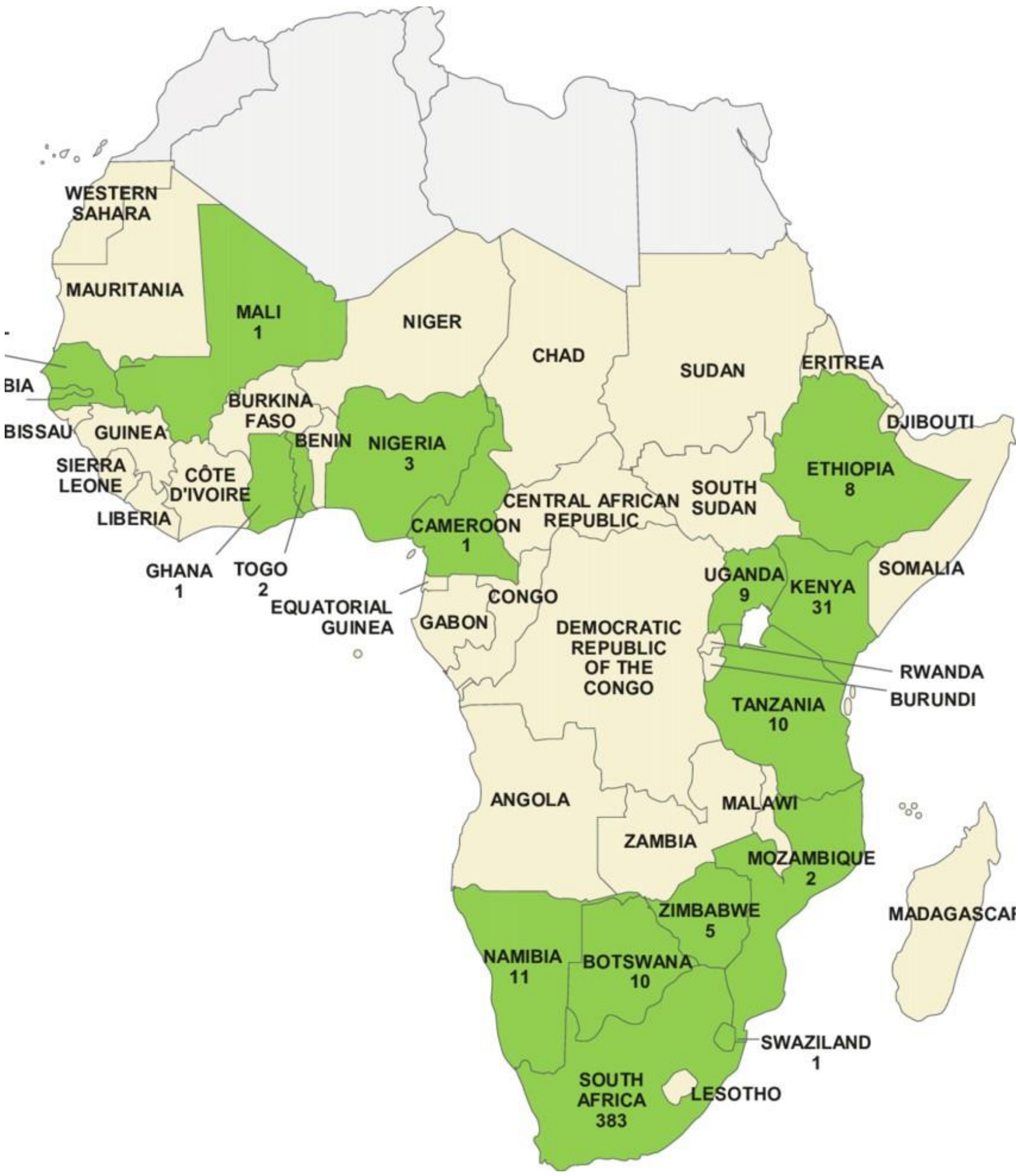

Fig 2. Map of SSA showing the geographic distribution of dementia-related studies across countries. The green shading indicates the countries represented in the included literature, while the beige shading indicates the countries where no relevant studies were identified. The numbers refer to the number of included studies from each country [25]

2.4 Study Selection Process

All records retrieved from the databases and other sources were compiled and duplicates removed before screening. Two steps are followed in selecting studies for the systematic review. First, the titles and abstracts are used to exclude studies that are not relevant to the review question. Second, the full-text articles are used to select the studies based on the inclusion criteria. Any doubt about the inclusion of a study is resolved through discussion among the review team. The process of selecting studies, including the exclusion of irrelevant studies, is presented in a flow diagram to ensure transparency in the reporting of the review process.

2.5 Synthesis of Results

The extracted data are then synthesized using descriptive and thematic analysis to establish the status of dementia care in SSA. This synthesis differs from most data synthesis approaches, which aim to combine data quantitatively. Still, in this case, the gaps identified in four major areas, which include health systems capacity gaps, caregiving support gaps, service delivery and access gaps, and technology and innovation gaps, are targeted. The data synthesized is categorized under thematic areas, which include health system capacity gaps such as financial, economic, environmental, and social constraints, caregiving gaps such as training and support for informal and community-based caregivers, service delivery and access gaps such as fragmented care processes and access to care, and technology and innovation gaps such as the use and availability of technology in dementia diagnosis, care, and patient monitoring.

Figure 1 illustrates the PRISMA flow diagram, which outlines the study identification, screening, eligibility, and inclusion process for the recent literature on dementia care gaps in SSA. Figure 2 presents a map of SSA highlighting the geographic distribution of dementia-related studies across countries.

3 Environmental and social challenges

This section focuses on the environmental, social, and systemic drivers that shape dementia diagnosis and management across Sub-Saharan Africa. Based upon a retrospective analysis from Northeastern Nigeria in which Wakawa et al. [26] examined over two decades of patient records from the region's sole neuropsychiatric facility, 1,216 cases of dementia among adults aged 60 years and older were documented. Alzheimer's disease (60.5%) and vascular dementia (24.5%) were the most prevalent subtypes, with hypertension (41.6%) as the leading comorbidity. There was a limited diagnostic capacity due to a lack of neuroimaging tools, biomarkers, and a specialized workforce. This reflects the broader environmental and infrastructural constraints of dementia care in resource-limited regions. The authors emphasize that both vascular comorbidities and environmental stressors, such as chronic hypertension and conflict-related trauma, may contribute to the region's escalating dementia rates, underscoring the need for policy reforms that strengthen neurological health infrastructure and surveillance systems.

Similarly, Wisdom et al. [27] identified environmental and occupational exposures as important but often overlooked contributors to neurodegenerative disease in Nigeria, including fine particulate air pollution, pesticide exposure, and groundwater contamination with heavy metals. Their analysis estimated that dementia prevalence has risen by nearly 400% over two decades, disproportionately affecting aging and low-income populations. The authors argue for integrating dementia prevention into environmental health policies, noting that Nigeria's current health governance lacks coherent frameworks linking neurodegenerative disease to ecological regulation or

Commented [J24]: Should specify inter-reviewer agreement method (e.g., Cohen's kappa).

Commented [J25]: Grammatical issue → "data are synthesised and categorised".

non-communicable disease (NCD) control. Moreover, the concentration of neurologists and diagnostic centers in urban areas amplifies rural inequities and delays in diagnosis.

In South Africa, Ssonko et al. [28] conducted the first longitudinal analysis of memory clinic data from Groote Schuur Hospital in Cape Town, revealing that Alzheimer's disease (70.9%) and vascular dementia (14.6%) accounted for the majority of 165 dementia cases. The study found that cognitive decline averaged 2.1 Mini-Mental State Examination (MMSE) points per year for Alzheimer's and 1.3 for vascular dementia. Educational attainment and cardiovascular comorbidities, linked to environmental and lifestyle factors such as diet and urbanization, emerged as significant predictors of both cognitive decline and mortality. The study situates these findings within broader socioeconomic and infrastructural determinants, arguing that the limited formalized dementia registries and geriatric services in SSA hinder effective governance and continuity of care.

Beyond environmental stressors, social and cultural determinants deeply influence dementia risk, diagnosis, and management across SSA. Bosire et al. [29] conducted ethnographic research at the Aga Khan University Hospital in Nairobi, documenting healthcare providers' perspectives on dementia diagnosis and management in Kenya. Although tertiary private hospitals offered multidisciplinary, globally aligned diagnostic pathways, providers highlighted the severe inequities between the private and public sectors. Most Kenyans lack access to neuroimaging, cognitive assessments, and specialized care from trained professionals. Stigma, misconceptions linking dementia to witchcraft or punishment, and the absence of a national dementia policy were recurrent themes. These sociocultural barriers delay care-seeking, limit early detection, and increase the burden on caregivers. The authors advocate for Kenya's first national dementia strategy to bridge clinical, cultural, and governance divides.

In Nigeria, Wisdom et al. [27] further illuminated how gender, education, and social position intersect with stigma to shape the lived experience of dementia. Older women, in particular, face discrimination and violence due to widespread beliefs that cognitive decline results from witchcraft. Informal caregivers, predominantly women, bear over 70% of unpaid care hours, often without institutional or financial support. These findings highlight important governance and policy implications, suggesting that dementia should be framed not only as a medical condition but also as a social issue with relevance to aging and gender equity policies.

While pharmacological interventions remain limited, psychosocial and behavioral strategies have shown promise for addressing this issue. Beyene et al. [30] conducted the first systematic review of dementia management practices in SSA, analyzing randomized clinical trials and cohort studies across eight countries. They found that Cognitive Stimulation Therapy (CST), a low-cost, group-based behavioral intervention, was feasible and effective in improving cognitive and language outcomes in low-resource settings. Furthermore, early initiation of combination antiretroviral therapy was associated with improved cognitive outcomes among individuals with HIV-associated dementia (HIVAD). The review also revealed systemic gaps, including a lack of culturally validated diagnostic tools, inadequate training of community health workers, and limited support networks for caregivers. The authors call for scaling up CST and integrating dementia management into primary healthcare systems through task-shifting and training initiatives. These findings align with Wakawa et al. [26], who advocate for decentralized care models to extend dementia services beyond tertiary facilities. The establishment of the Northern Nigeria Dementia Research Group marks a significant step toward creating a national dementia registry and promoting the translation of research into policy and practice. Both studies emphasize the importance of governance structures that facilitate early detection, community-based care, and the sharing of regional data. Figure 3 summarizes the environmental, cultural, and social

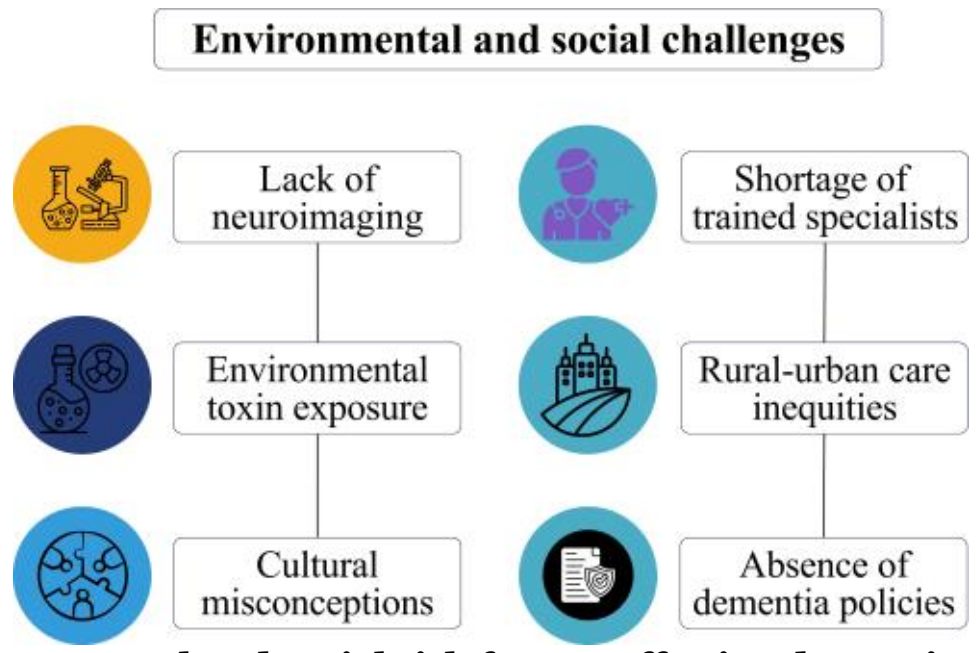

Fig 3. Environmental and social risk factors affecting dementia diagnosis in SSA. Dementia diagnosis in SSA is shaped by interacting environmental, social, and structural constraints, including limited access to neuroimaging and trained specialists, as well as pronounced rural–urban inequities in diagnostic services.[1,2] Sociocultural misconceptions, stigma, and the absence of formal dementia policy frameworks further delay care-seeking and hinder early detection.[3] **Notes:** [1] Diagnostic capacity and specialist workforce limitations in resource-constrained settings [26, 28]. [2] Environmental and occupational exposures linked to neurodegenerative risk [27]. [3] Sociocultural barriers, stigma, and policy absence affecting dementia recognition and diagnosis [29].

factors contributing to disparities in dementia care across SSA.

Collectively, these studies demonstrate that dementia in SSA is shaped by intersecting environmental, social, and systemic factors that demand an integrated governance response. Three broad policy imperatives emerge. First, it is essential to integrate dementia into the National Health Frameworks and plans. Dementia should be formally embedded within national aging, NCD, and environmental health policies. Governments can align with the WHO Global Action Plan on the Public Health Response to Dementia (2017–2025) by developing country-specific dementia strategies, establishing registries, and promoting caregiver protections. Secondly, capacity building and decentralization of efforts are key. Investment in workforce training, particularly in task-shifting, primary care, and community health workers, is essential. There is also evidence that this approach, supported by findings from Ethiopia, Nigeria, and Kenya, can bridge diagnostic and treatment gaps in rural regions. Finally, cross-sectoral and international collaboration is essential. Effective governance needs to extend beyond the health sector to address the broader social and environmental determinants of dementia. Integration with ecological regulation (air quality, pesticide control), education, and social welfare systems is key. Furthermore, partnerships between African governments, research consortia, and global funding bodies can support evidence-based policymaking and the development of research infrastructure.

The growing body of dementia research in SSA provides critical insights into how environmental exposures and social inequities converge to shape cognitive aging. Across Nigeria, Kenya, South Africa, and Ethiopia, recent evidence suggests the need for urgent governance reforms that integrate environmental stewardship, social protection, and health system strengthening. By reframing dementia as both a public health and a social governance challenge, SSA can lead the development of a new, integrated, and equity-centered global health policy model for the prevention and care of neurodegenerative diseases.

4 Technology-related challenges

Digital health technologies can enhance dementia care in low-resource settings; however, their adoption across SSA remains minimal due to infrastructural, financial, and training limitations. Internet connectivity is inconsistent and often unavailable in rural areas, and many healthcare facilities lack a reliable power supply and the necessary hardware to conduct telemedicine and remote consultations [31, 32]. Telemedicine, tele-support for caregivers, has been investigated as one of the solutions for extending care for dementia patients, but the development of this type of service in SSA is uneven due to problems with information technology infrastructure. The systematic reviews of the effects of telemedicine in low and middle-income countries, as well as SSA, imply the potential of telemedicine to improve access to specialist consultation and caregiver support, while also identifying persistent barriers related to infrastructure reliability, workforce training, and integration with existing health systems [33, 34]. One key area that could be improved by applying technological advancements to SSA's support structure for dementia is standardization and greater accessibility of screening. With so many different cultural settings and linguistic patterns across SSA, a lack of standardized screening methods could merely reflect a scarcity of understanding and preparation within healthcare. Frontline health workers in many countries misclassify dementia symptoms with "normal aging," "stress," or general cognitive decline as a person gets older [35, 36]. Training challenges, cultural adaptation, and health system preparedness are some of the reasons for the unequal administration of cognitive assessment tests like the Mini-Mental State Examination (MMSE) and the Montreal Cognitive Assessment (MoCA), especially in cases where test content is unavailable in local languages or culturally adapted forms across SSA [31].

Another technological barrier is the limited availability of laboratory infrastructure, neuroimaging, and biomarker assays for diagnosing dementia. Positron emission tomography scans, molecular imaging, and Cerebrospinal Fluid (CSF) biomarker testing are cutting-edge technologies available in high-income countries but are limited in SSA [26]. Moreover, in SSA, access to MRI imaging, which is essential for dementia diagnosis, is severely constrained by restricted access, high costs, and frequent equipment downtime [27]. Due to limited access to diagnostic technologies, health care practitioners are often left to diagnose based on clinical history alone, making it difficult to distinguish dementia from other causes of intellectual impairment, such as tumors, stroke, and hydrocephalus. This diagnostic uncertainty increases the risk of dementia being underdiagnosed or misdiagnosed. For instance, in rural Kenya and Nigeria, cognitive symptoms can be misattributed to depression, epilepsy, or psychiatric conditions because of the lack of appropriate screening tools and specialized diagnostic equipment [27, 37]. Moreover, Yenesew et al. [38] describe how East African research organizations lack the capacity in biomarker, genetic, and epigenetic research, further limiting region-specific understanding of dementia etiology. This also limits the infrastructure, such as screening tools, culturally adapted interventions, and predictive models of dementia risk. Furthermore, limited coordination among dementia-related data systems may create a cyclical barrier, hindering investment in technology due to insufficient data. Without technology, data cannot be collected and studied properly. Without these basic technologies, SSA countries remain constrained in producing population-level data on dementia subtypes and risk factors. Combined with limited financial resources, structural barriers linked to laboratory infrastructure, biomedical engineering capacity, and maintenance support further restrict the use of modern diagnostic technologies for dementia diagnosis and care. These limit the use of modern technologies in diagnostic equipment for proper dementia care.

The technological gap is also deeply interconnected with human resource shortages. Across SSA, there is a scarcity of individuals with expertise in dementia care, including

neurologists, geriatricians, psychiatrists, and neuropsychologists [35, 39, 40]. With a population of 200 million people, Nigeria has only 90 neurologists, substantially constraining the diagnostic capacity of neurological conditions, including dementia [27]. Due to a lack of specialists, mobile and digital screening tools often yield unsatisfactory results, as these tools require confirmatory diagnosis and clinical oversight [41]. Primary healthcare workers often report low confidence in cognitive assessments and in distinguishing dementia from other neurological or psychiatric disorders because of the limited dementia content in medical and nursing curricula. This contributes to diagnostic misclassification, delayed referrals, and the perpetuation of stigmatizing opinions about dementia [36]. The specialist shortage is therefore not simply a workforce issue; it is also a structural technological hindrance that restricts the performance of diagnostic tools, reduces the usefulness of digital screening innovations, and restricts the establishment of multidisciplinary dementia clinics [41]. Access to pharmacological remedies, including acetylcholinesterase inhibitors (donepezil, rivastigmine) and memantine, is minimal due to cost, stock-outs, or absence from national formularies [27, 31].

Studies from countries including Ghana, South Africa, and Tanzania reveal similar trends: healthcare providers often lack essential tools to diagnose congenital changes and functional decline, despite observing these changes [39–41]. Mobile health

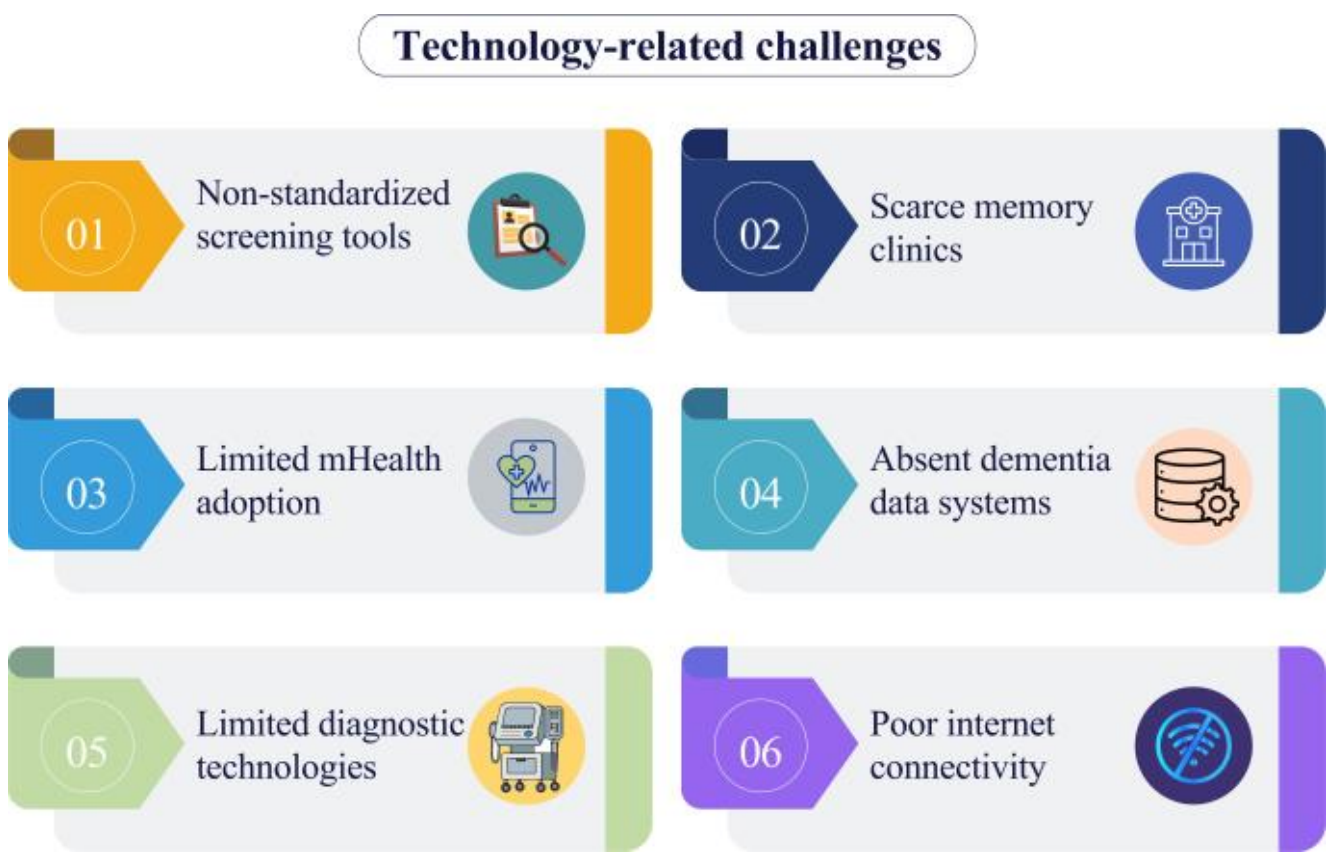

Fig 4. Technology gaps and limitations affecting dementia care. Dementia care in SSA is constrained by fragmented and non-standardized screening tools, limited diagnostic technologies, and weak digital health infrastructure, hindering early diagnosis and continuity of care.[1,2] Structural barriers, including scarce memory clinics, weak referral pathways, and shortages of trained specialists, further limit the effectiveness of technology-enabled interventions.[3] Persistent system-level constraints affecting telemedicine, mHealth adoption, and dementia data systems remain evident across the region.[4] **Notes:** [1] Limited cultural adaptation of cognitive screening tools [31, 35]. [2] Restricted access to neuroimaging and biomarker-based diagnostics [26, 27]. [3] Shortages of specialists and memory clinic infrastructure [39, 40]. [4] Infrastructure barriers affecting telemedicine and dementia data systems [32, 42].

(mHealth) systems play a crucial role in caregiver education, symptom monitoring, and early disease detection. However, studies indicate that increased dropout rates, usability challenges, and poor integration with existing health care strategies are issues in SSA [31, 42]. App-based screening tools deployed in Tanzania show potential, but there are some limitations, including the fact that digital screening cannot substitute confirmatory diagnosis, and referral pathways to specialist care remain fragile [41]. Moreover, Paddick et al. demonstrated the feasibility of using a tablet-based dementia screening app that incorporated culturally adapted cognitive assessment items and was administered by non-specialist community health workers. However, they highlighted the limited scope of technology-driven approaches in the absence of broader systemic changes to address issues such as limited specialist referral capacity and insufficient training. Further, without validation, scalable screening technology, and a robust health strategy, early detection will remain unstable. SSA countries cannot fully leverage the benefits of telemedicine or digital caregiver support without national digital health strategies, electronic health records, and stable digital networks. One of the most pressing challenges is the absence of dedicated, interoperable dementia-specific data systems, despite the presence of broader digital health infrastructures such as electronic health records and DHIS2 platforms in countries including Nigeria, Kenya, and Ethiopia [32, 42]. Without this mechanism, it is challenging for policymakers to make a decision on the prevalence, geographic allocation, and socioeconomic effects of dementia. Figure 4 illustrates the key technology gaps and limitations that affect dementia diagnosis and care delivery in SSA.

5 Community-based dementia care and informal caregiving challenges

This subsection discusses community-based dementia care and informal caregiving, which aligns with the WHO's health system building block of service delivery and its focus on people-centered, community-oriented care. Informal care is typically provided in multigenerational households with limited resources and minimal support from the formal health system [43], and caregiving demands increase with increasing severity of behavioral and psychological symptoms of dementia (BPSD) [44]. Studies predominantly reported women as informal caregivers for PLWD, with proportions ranging from 70% to 90% across settings in Uganda, Benin, Tanzania, Ghana, and the Democratic Republic of Congo. Caregivers themselves are often older adults or middle-aged individuals, frequently spouses or adult daughters of care recipients [43, 45–48]. Studies also reveal limited knowledge about dementia, and high levels of unmet support needs among caregivers [43, 49, 50]. While explicit quantification was not available across these studies, the Benin time-use evaluation study estimated that caregivers spend 8-12 hours/day on informal caregiving activities like supervising the PLWD, assisting with activities of daily living (ADL), and managing behavioral symptoms [48].

Studies reported higher caregiving burden across the different country settings. Three studies specifically reported caregiving burden using a standardized Zarit Burden Interview (ZBI) that assesses subjective caregiver burden and reported moderate to severe burden among informal caregivers of PLWD [45–47]. Studies also reported psychological distress among caregivers with reports of high prevalence of depression, anxiety, and emotional exhaustion. Okwaraji et al. [51] reported a strong association between BPSD severity and burnout and psychological stress among caregivers in Nigeria. They provide the most detailed quantification of caregiver burden, with 40% of caregivers experiencing psychological distress and nearly 25% experiencing emotional exhaustion. Functional impairment and higher BPSD severity were also associated with

greater supervision needs and frequent nighttime caregiving requirements [44, 47].

Studies also showed a significant economic burden associated with dementia caregiving in the SSA setting. Caregiving can disrupt employment, as caregivers often struggle to balance their work and caregiving responsibilities. Caregivers often report reducing work hours, leaving jobs entirely, or shifting to informal labor to accommodate caregiving responsibilities [48, 51, 52]. Kenne Malaha et al. [48] provided a direct economic quantification of dementia care costs in Benin, estimating the annual dementia care cost in 2021 to be purchasing power parity dollars (PPP$) 2,399. An estimated 92% of this cost was attributed to informal care. This high number underscores the substantial economic burden borne by families when caring for people with learning disabilities. A national situational analysis from South Africa linked dementia-related care expenditures (not just limited to informal caregiving-related expenses) to chronic financial instability [52].

Information on interventions and their effectiveness is limited by study design and lack of generalizability. A study in Uganda found positive associations between interventions such as gardening and improved mental health scores, as well as lower psychological distress among caregivers [54]. A singing training program in South Africa showed associations with improved dementia caregiver confidence [55]. A community intervention focused on stigma reduction demonstrated high rates of acceptance and feasibility, with marked improvements in caregivers' attitudes towards dementia [56].

Health system resource constraints severely impact PLWD and their caregivers in SSA, both directly and indirectly. Sparse dementia specific services that remain

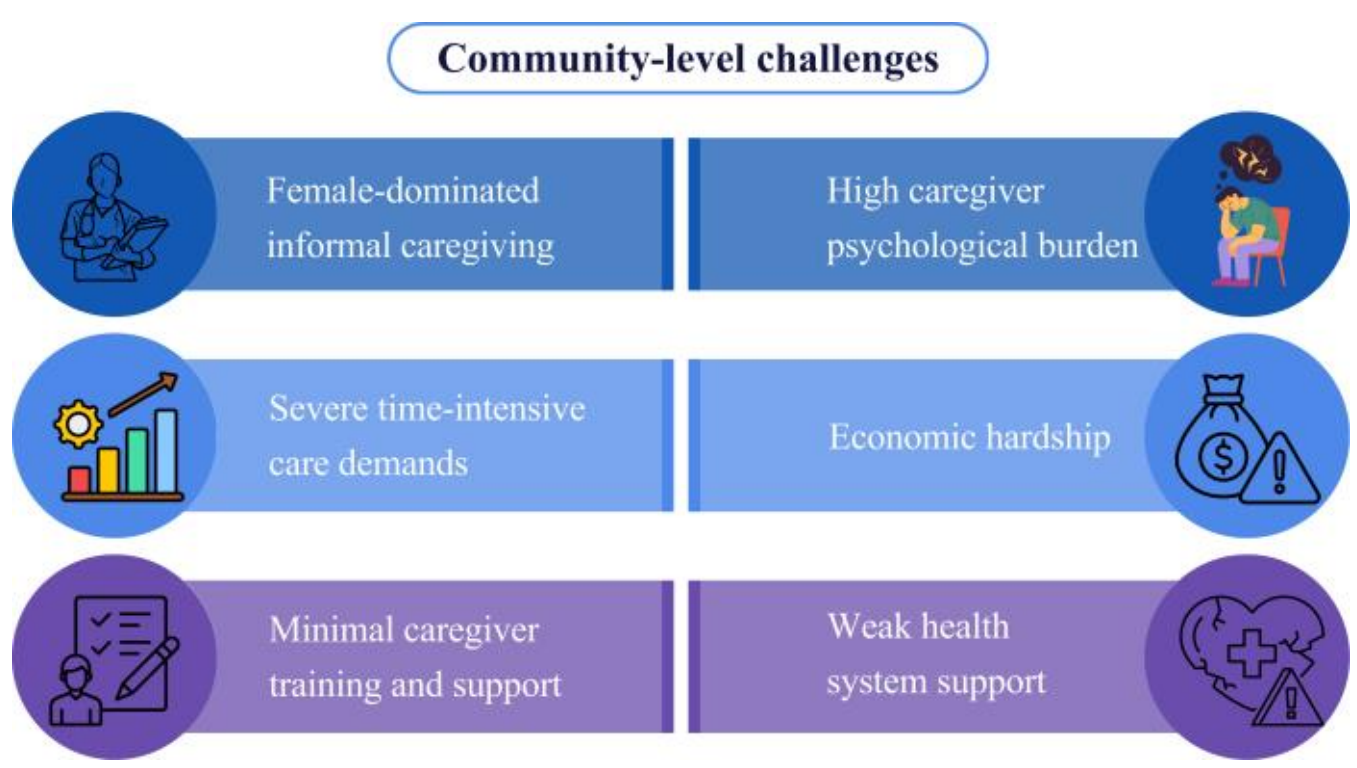

Fig 5. Community-based dementia care and informal caregiving in SSA. Evidence indicates that dementia care in SSA is predominantly home and community-based, with informal caregiving largely undertaken by women and characterized by substantial time demands and psychological burden.[1,2] Limited caregiver training, delayed diagnosis, and weak health-system support further shift responsibility to households, contributing to economic strain and unmet care needs.[3,4] **Notes:** [1] Gendered informal caregiving roles and caregiver burden [43, 46, 47]. [2] Associations between behavioral and psychological symptoms of dementia and caregiver distress [44, 45, 51]. [3] Time-intensive caregiving and economic impacts of informal care [48, 52]. [4] Health-system constraints, delayed diagnosis, and limited service availability [29, 52, 53].

fragmented, underdeveloped, or absent place a heavy burden on informal caregivers [52]. Underdiagnosis or delayed diagnosis of dementia due to a lack of resources creates a cycle of unmanaged symptoms and delayed care with implications for caregivers. Workforce shortages and gaps, including low provider knowledge, may impact care-seeking behavior [52, 53]. The responsibility of managing undiagnosed PLWD falls squarely on family members. Informal caregivers themselves receive little to no training on managing dementia symptoms. Unreliable access to medicines, coupled with a lack of information and stigma, may drive families to seek traditional healers, further delaying medical diagnosis and treatment [57]. A lack of financial protection and dependence on out-of-pocket household spending lead families into catastrophic health spending. This financial instability is compounded by the loss of caregiver income [48, 52].

The evidence from SSA highlights substantial caregiver burden, limited or non-existent caregiver support, and gendered caregiving norms, all of which are further compounded by weakened health system infrastructure. Figure 5 depicts informal caregiving dynamics and locally rooted support systems for dementia care in SSA.

6 Financial and economic challenges

Financial and economic barriers remain central determinants of access, quality, and sustainability of dementia care across SSA. Three recent studies included estimates of dementia costs in SSA. Two of these studies were global dementia cost studies that reported estimates of dementia costs in SSA [58, 59]. The remaining one [60] highlighted results from an earlier study, Guerchet et al. [8]. In Pedroza et al. [58], the methodology relied on a systematic review of the published literature to generate estimates of costs associated with dementia care. These studies mainly captured costs related to spending on direct health services or monetary costs. Because there are very few cost studies based on primary data published for people living with dementia in SSA to inform modeling, their estimates relied on modeling costs based on covariates and assumptions. The results from the modelling study show that in 2019, for SSA, the spending attributable to dementia was $552 million (Uncertainty interval: $325-$816 million), 0.7% of total health spending. This estimate was projected to rise to $6,955 million ($3,401 - $12,001 million) by 2050. The second study, Chen et al. [59], reported data on the economic costs (including both monetary and non-monetary costs) of dementia in SSA, based on a macroeconomic model simulation. Specifically, the authors used a health-augmented macroeconomic model to estimate the macroeconomic burden of Alzheimer's disease and other related dementias (ADODs). Their model accounted for labor supply effects for the person living with dementia (morbidity and mortality) as well as for their caregivers (reduction in hours of work), as well as age-sex differences, education, work experience, treatment, and formal care costs, diverting from investments and savings. The authors estimate that between 2020 and 2050, the economic burden of ADODs in SSA will be approximately $158 billion (126-199) in international dollars. This translates to 0.108 as a proportion of total economic output (gross domestic product) between 2020 and 2050. Given the rising proportion of the population above the age of 60, these costs could potentially increase rapidly if the appropriate policies and interventions are not implemented to manage them.

While there exists anecdotal and qualitative evidence that the indirect costs, such as informal caregiving, constitute substantial aspects of the economic burden of dementia care for families with people living with dementia, there is limited observed or documented data on these costs. The few existing estimates are modelled approximations from earlier years [60]. It is reported that in 2015, dementia cost SSA $ 6.2 billion, of which more than two-thirds (70%) was attributable to informal care provided by the families of people living with dementia. Furthermore, the existing

structure of care for people living with dementia in most SSA countries relies predominantly on families providing most of the non-medical support required by people living with dementia. The high proportion of carers signals a potentially enormous cost to national output that is unaccounted for. This is an unaccounted cost because the economies of several SSA countries have significant informal sectors; therefore, such support provided informally is likely under-reported in official records. As such, quantifying non-financial and informal costs will require innovative documentation and analysis.

The existing literature mostly reports modeling costs based on limited data. Hence, the evidence base has yet to focus on the sources and mechanisms that people living with dementia in SSA use to pay for care. However, there is some evidence on how older adults generally pay for care in SSA. This broader literature base suggests a heavy reliance on household out-of-pocket spending [61, 62]. Additionally, given the general disease burden in SSA countries, we observe that even in countries with national health insurance systems, the benefits plan tends to prioritize infectious diseases over non-communicable diseases and may not always cover medications. Nonetheless, older populations may be exempt from paying enrollment or premium fees to access benefits under national health insurance programs. Typically, the burden of financing falls on the individual and their family [63]. These include not only direct spending on consultation visits, medication procurement, and diagnostics, but also the bulk of the responsibility for assisting with activities of daily living when necessary, as part of the care plan. In

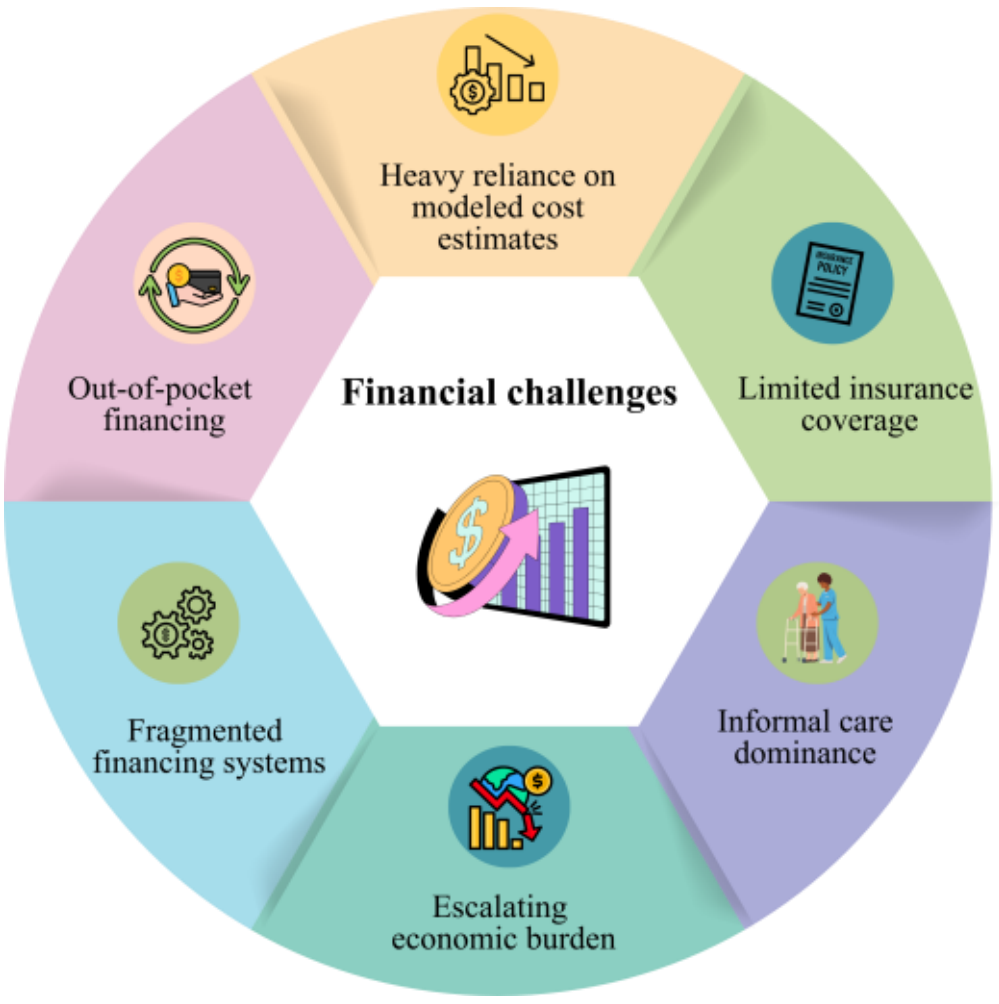

Fig 6. Financial and economic challenges shaping dementia care systems in SSA. The literature indicates a reliance on modeled cost estimates due to limited primary cost data[1], a predominant dependence on household out-of-pocket financing[2], and largely informal, family-based caregiving responsibilities that disproportionately fall on women[3]. These challenges are compounded by population aging and the rising burden of cardiovascular and neurological diseases[4]. **Notes:** [1] Synthesized evidence from cost modeling studies in SSA. [2] [61, 62] [3] [63] [4] [31]

most SSA countries, this responsibility falls predominantly on women within families.

The potential cost drivers in SSA include a rising burden of stroke and other cardiovascular diseases, an aging population, and a population with a relatively lower education level [31]. Understanding the drivers of these financial and economic costs may help shed light on the broader societal implications for the future and highlight potential pathways for addressing, managing, and responding appropriately. While the nascent stage of this research topic means that there are limited studies that primarily explore the determinants of financial and economic costs borne by people living with dementia, there are descriptive suggestions of potential cost drivers in the limited epidemiological studies that do exist. The cost drivers highlighted above suggest that a response strategy focused on a life-course and prevention-oriented approach might be appropriate to keep both financial and non-financial costs within manageable limits.

Leveraging the World Health Organization's six building blocks of a health system framework, the financing principles could identify potential pathways for countries in SSA to comprehensively cover the financial and economic costs of dementia care for people living with dementia and their families. The financing block proposes three functions of a financing system: mobilizing revenue, pooling resources, and purchasing services. Mobilizing revenue refers to the process of raising funds to cover the costs of health services. Pooling addresses the importance of sharing risks across groups by aggregating individuals and resources into groups, enabling them to benefit from economies of scale. Lastly, purchasing refers to the collective mobilization of resources to procure the necessary health goods and services. Countries in SSA vary in their macroeconomic contexts, fiscal space, and potential for revenue mobilization; as such, each country's approach to developing a comprehensive financing plan for dementia will need to take local constraints into account. Nonetheless, there are global examples of approaches to revenue mobilization, pooling, and purchasing that countries can draw on to develop contextually appropriate responses. For SSA in particular, a key consideration is the relatively young population and the opportunities it presents to develop a financing strategy that prioritizes a prevention-oriented approach to harness gains from a preemptive stance. In terms of specific examples, South Korea and Brazil offer models for pursuing a preventive approach and leveraging a universal health care platform to provide comprehensive coverage for dementia treatment, as well as the formal caregiving support required in later stages through long-term care coverage. Figure 6 illustrates key financial and economic barriers influencing the organization and sustainability of dementia care systems in SSA.

In summary, the financial and economic costs of dementia in SSA are an area that is underexplored and underrepresented in the current literature. More primary data on costs by severity, age, sex, and caregiver-related factors across the different countries in SSA are needed. While SSA is currently considered a relatively young population, its older population is set to increase substantially by 2050. Understanding the potential financial and economic ramifications fully will be essential for planning appropriately on the continent.

7 Discussion

This review highlights the challenges in dementia care in SSA and addresses both social and economic issues. The literature reviewed identifies technology gaps as a significant barrier to early diagnosis, effective monitoring, and coordinated care. Many countries continue to face limited cognitive screening capacity, digital health infrastructure, and trained personnel to deploy telemedicine-based support tools. At the same time, several emerging technologies, including mobile screening applications and community-based digital tools, show promise for improving dementia care in SSA. For example, expanding

mobile phone use, growing digital health initiatives, and the increasing prioritization of non-communicable diseases in global health agendas provide a platform for developing and testing cost-effective, sustainable, and innovative approaches to dementia care. However, challenges related to scalability, limited internet access in rural areas, and insufficient frontline provider training complicate implementation.

Cultural misconceptions and low dementia literacy prevent families from seeking help and following care tracks, and environmental and social risks compound disparities. Rural residents face a higher burden while trying to access specialized services due to inaccessible services and weaker primary care networks. Social factors, including poverty, gender roles, and limited caregiver education, impact caregiver burden and patient outcomes. Our review highlights the role of informal caregivers, primarily women, who fulfil the role of primary caregiver without training, respite services, or psychosocial support. The emotional, physical, and economic strain consistently documented in the included studies underscores the need for structured training programs, community outreach, and support initiatives to improve caregiver well-being.

In many SSA countries, patients lack insurance coverage and have limited access to government-funded services, including formal care, diagnostic services, and medications. Very few countries have national dementia policies, and these findings emphasize the urgent need for integrated, context-specific solutions that address structural, technological, and social barriers simultaneously. In addition to funding, the results also indicate a set of interrelated weaknesses in the other building blocks of health systems, as defined by WHO, such as service delivery, health workforce capacity, health information systems, and governance, which collectively contribute to the fragmentation and scalability challenges of dementia care in SSA.

A significant strength of this review is its synthesis of the recent literature (2021-2025), which provides an overview of the challenges and technological opportunities in the care of individuals living with dementia in SSA. Additionally, the thematic synthesis helps capture the various aspects of the care system, caregivers, and technology that have otherwise been treated in isolation. However, the review has several limitations. First, the literature review is based on documented literature, which may not capture the undocumented community practices. Additionally, the heterogeneity of studies across countries in SSA makes direct comparisons difficult. Finally, the review is based on a narrative synthesis, which did not allow for calculating intervention efficacy. Nonetheless, the findings of the review help guide the development of the literature, policies, and technology in the care of individuals living with dementia in SSA.

8 Conclusion

Despite these systemic challenges, this period also presents a critical chance to enhance dementia research and care in SSA, in the wake of increasing policy attention to aging, the development of digital health infrastructure, and improved collaboration. Dementia care in SSA is significantly shaped by technological, social, community, and economic disparities that limit early diagnosis, treatment, and care. Using the WHO health system framework as an analytical lens, this review emphasizes how gaps in health financing, service delivery, and workforce capacity contribute to the absence of a comprehensive and sustainable dementia care system in SSA. This review implies the need to develop more national policies, digital and primary care infrastructure, caregiver center programs, and public awareness. Supporting community-based dementia care models, investing in affordable technologies, and expanding financial services can improve access to dementia services. Further research should focus on locally available mechanisms, culturally sensitive approaches, and investing in more scalable and

Commented [J26]: discussion; tighten and focus on contributions.

Commented [J27]: Informal phrasing; replace with "critical opportunity".

sustainable models. Addressing these multifaceted gaps is essential for improving the quality of life for people with dementia and ensuring equitable, accessible, and sustainable dementia care services in SSA. In addition, workforce development training programs are an integral component of dementia care services. Finally, developing region-specific data warehouses can aid policymakers and reveal potential interventions for SSA.

Commented [J28]: Excellent recommendation; could be expanded

References

Commented [J29]: Missing DOIs in several citations.

1. Prynn JE, Davey C, Davis D, Kuper H, Mugisha J, Seeley J. Cognition in older adults in Uganda: Correlates, trends over time and association with mortality in prospective population study. PLOS global public health. 2023;3(11):e0001798.

2. Brooke J, Ojo O. Contemporary views on dementia as witchcraft in sub-Saharan Africa: A systematic literature review. Journal of clinical nursing. 2020;29(1-2):20-30.

3. Adebisi AT, Salawu MA. Misconception of dementia-related disorders in Sub-Saharan Africa. Frontiers in Neurology. 2023;14:1148076.

4. Mavrodaris A, Powell J, Thorogood M. Prevalences of dementia and cognitive impairment among older people in sub-Saharan Africa: a systematic review. Bulletin of the World Health Organization. 2013;91:773-83.

5. Olayinka OO, Mbuyi NN. Epidemiology of dementia among the elderly in sub-Saharan Africa. International Journal of Alzheimer's disease. 2014;2014(1):195750.

6. Wakawa IA, Musami UB, Kwairanga SH, Ogualili PN, Mahmood MY, Fugu MA, et al. Management of Dementia in a Resource-Constrained Sub-Saharan African Setting: Outcome of a Retrospective Survey of Clinical Practice in the Only Neuropsychiatric Facility in Northeastern Nigeria. medRxiv. 2024:2024-09.

7. Prince M, Wimo A, Guerchet M, Ali GC, Wu YT, Prina M. World Alzheimer report 2015. The global impact of dementia: an analysis of prevalence, incidence, cost and trends.; 2015.

8. Guerchet M, Mayston R, Lloyd-Sherlock P, Prince M, Akinyemi R, Paddick SM, et al. Dementia in sub-Saharan Africa: Challenges and opportunities. London, UK: Alzheimer's Disease International; 2017.

9. Nichols E, Steinmetz JD, Vollset SE, Fukutaki K, Chalek J, Abd-Allah F, et al. Estimation of the global prevalence of dementia in 2019 and forecasted prevalence in 2050: an analysis for the Global Burden of Disease Study 2019. The Lancet Public Health. 2022;7(2):e105-25.

10. Akinyemi RO, Owolabi MO, Ihara M, Damasceno A, Ogunniyi A, Dotchin C, et al. Stroke, cerebrovascular diseases and vascular cognitive impairment in Africa. Brain research bulletin. 2019;145:97-108.

11. Organization WH, et al. Global status report on the public health response to dementia. Geneva, Switzerland: World Health Organization; 2021. Available from: https://www.who.int/publications/i/item/9789240033245.

12. Apeagyei AE, Lidral-Porter B, Patel N, Solorio J, Tsakalos G, Wang Y, et al. Financing health in sub-Saharan Africa 1990–2050: Donor dependence and expected domestic health spending. PLOS Global Public Health. 2024;4(8):e0003433.

13. Violante F, Collaborators GD, et al. Global age-sex-specific fertility, mortality, healthy life expectancy (HALE), and population estimates in 204 countries and territories, 1950-2019: a comprehensive demographic analysis for the Global Burden of Disease Study 2019. The Lancet. 2020;396(10258):1160-203.

14. Yoseph M, Paddick SM, Gray WK, Andrea D, Barber R, Colgan A, et al. Prevalence estimates of dementia in older adults in rural Kilimanjaro 2009–2010 and 2018–2019: is there evidence of changing prevalence? International Journal of Geriatric Psychiatry. 2021;36(6):950-9.

15. Mushi D, Rongai A, Paddick SM, Dotchin C, Mtuya C, Walker R. Social representation and practices related to dementia in Hai District of Tanzania. BMC Public Health. 2014;14(1):260.

16. Ogunniyi A, Hall K, Baiyewu O, Gureje O, Unverzagt F. Caring for individuals with dementia: the Nigerian experience. West African journal of medicine. 2005;24(3):259-62.

17. Aboderin IA, Beard JR. Older people's health in sub-Saharan Africa. The Lancet. 2015;385(9968):e9-e11.

18. Amoakoh-Coleman M. Tackling Ghana's dementia underdiagnosis needs investment in workforce, diagnostics, education and insurance. Nature Aging. 2025:1-1.

19. Okoroh JS, Riviello R. Challenges in healthcare financing for surgery in sub-Saharan Africa. Pan African Medical Journal. 2021;38(1).

20. Wimo A, Guerchet M, Ali GC, Wu YT, Prina AM, Winblad B, et al. The worldwide costs of dementia 2015 and comparisons with 2010. Alzheimer's & Dementia. 2017;13(1):1-7.

21. Eze P, Lawani LO, Agu UJ, Acharya Y. Catastrophic health expenditure in sub-Saharan Africa: systematic review and meta-analysis. Bulletin of the World Health Organization. 2022;100(5):337.

22. Indicators AHO. Monitoring the building blocks of health systems. Geneva, Switzerland: WHO Document Production Services. 2010.

23. Doshmangir L, Sanadghol A, Kakemam E, Majdzadeh R. The involvement of non-governmental organisations in achieving health system goals based on the WHO six building blocks: A scoping review on global evidence. PLoS One. 2025;20(1):e0315592.

24. Tricco AC, Lillie E, Zarin W, O'Brien KK, Colquhoun H, Levac D, et al. PRISMA extension for scoping reviews (PRISMA-ScR): checklist and explanation. Annals of internal medicine. 2018;169(7):467-73.

25. Hassan A, Akintola MA, Hassan HB. Benchmarking public service delivery in Sub-Saharan Africa: A critical evaluation. International Journal of Research and Innovation in Social Science. 2024;8(7):2248-58.

26. Wakawa IA, Musami UB, Kwairanga SH, Ogualili PN, Mahmood MY, Fugu MA, et al. Dementia in a resource-constrained sub-Saharan African setting: A comprehensive retrospective analysis of prevalence, risk factors, and management at the only neuropsychiatric facility in Northeastern Nigeria. Alzheimer's & Dementia. 2025;21(3):e14538.

27. Kelechi Wisdom E, Soyemi T, Mayowa S, Ede NS, Ubalaeze Solomon E, Iloanusi CA, et al. Building healthcare capacity for neurodegenerative disease management in Nigeria: Challenges and opportunities. Journal of Public Health Research. 2025;14(2):22799036251350957.

28. Ssonko M, Hardy A, Naganathan V, Kalula S, Combrinck M. Dementia subtypes, cognitive decline and survival among older adults attending a memory clinic in Cape Town, South Africa: a retrospective study. BMC geriatrics. 2023;23(1):829.

29. Bosire EN, Blackmon K, Kamau LW, Udeh-Momoh C, Sokhi D, Shah J, et al. Healthcare providers perspectives and perceptions of dementia diagnosis and management at the Aga Khan University Hospital, Nairobi, Kenya. Journal of Alzheimer's Disease. 2025;104(3):862-74.

30. Beyene DA, Berha AB. Management Practice and Clinical Outcomes of Dementia in Sub-Saharan Africa: A Systematic Review. Behavioural Neurology. 2023;2023(1):2307443.

31. Alemayehu ZG, Ayalew BD, Sime BL, Bonger TD, Zewdie YA, Keraga AS, et al. Dementia in Sub-Saharan Africa: Risk Factors, Public Perception, and Management Approaches. Journal of Medicine, Surgery, and Public Health. 2025:100204.

32. Oyinlola O. Addressing the challenges of dementia care in Nigeria: a call for a comprehensive national strategy. Public Health Challenges. 2024;3(2):e186.

33. Dodoo JE, Al-Samarraie H, Alsswey A. The development of telemedicine programs in Sub-Saharan Africa: Progress and associated challenges. Health and Technology. 2022;12(1):33-46.

34. Adedeji A, Dogan H, Adedoyin F, Heward M. Telemedicine and Cultural Competency in Dementia Care: Mapping Stakeholder Roles in Digital Toolkit Development in SSA: A Systematic Review. Health Expectations. 2025;28(6):e70489.

35. Musyimi CW, Muyela LA, Mutiso VN, Mutunga E, Ndetei DM. Understanding dementia care pathways for policy development and service planning in Kenya. Dementia. 2023;22(5):1027-37.

36. Mwendwa P, Mutunga E, Kroll T, De Brún A. 'It is stressful, almost every hour. . . ': Experiences of caring for people living with dementia in Kenya—An interpretive phenomenological approach. Dementia. 2021;20(8):2916-32.

37. Aballa A, Khakali L, Njoroge W, Kamau LW, Merali Z, Bosire EN. "They are perceived to be witches, and some are killed": Community perceptions of elderly people living with dementia in Kilifi, Kenya. Journal of Alzheimer's Disease. 2025;103(2):593-604.

38. Yenesew MA, Krell-Roesch J, Fekadu B, Nigatu D, Endalamaw A, Mekonnen A, et al. Prevalence of Dementia and Cognitive Impairment in East Africa Region: A Scoping Review of Population-Based Studies and Call for Further Research. Journal of Alzheimer's Disease. 2024;100(4):1121-31.

39. Aryee CL. A Systematic Review of Creating an Ideal Adult Day Center for People with Dementia in Ghana (Sub-Saharan Africa). Cedar Falls, IA, USA: University of Northern Iowa; 2024. Available from: https://scholarworks.uni.edu/grp/4610.

40. Mahomed A, Pretorius C. Understanding the lived experiences of family caregivers of individuals with dementia in Soweto, a South African Township. Dementia. 2022;21(7):2264-87.

41. Paddick SM, Yoseph M, Gray WK, Andrea D, Barber R, Colgan A, et al. Effectiveness of app-based cognitive screening for dementia by lay health workers in low resource settings. A validation and feasibility study in rural Tanzania. Journal of Geriatric Psychiatry and Neurology. 2021;34(6):613-21.

42. Mwendwa P, Lawlor B, Kroll T, De Brún A. A systematic review and narrative synthesis of the experiences with caring for older people living with dementia in Sub-Saharan Africa. BMC geriatrics. 2022;22(1):961.

43. Ayisi-Boateng NK, Opoku DA, Tawiah P, Owusu-Antwi R, Konadu E, Apenteng GT, et al. Carers' needs assessment for patients with dementia in Ghana. African Journal of Primary Health Care & Family Medicine. 2022;14(1):3595.

44. Kamoga R, Mubangizi V, Owokuhaisa J, Muwanguzi M, Natakunda S, Rukundo GZ. Behavioral and psychological symptoms of dementia: prevalence, symptom severity, and caregiver distress in South-Western Uganda—a quantitative cross-sectional study. International Journal of Environmental Research and Public Health. 2023;20(3):2336.

45. Gumikiriza-Onoria JL, Nakigudde J, Mayega RW, Giordani B, Sajatovic M, Mukasa MK, et al. Psychological distress among family caregivers of persons with Alzheimer's disease and related dementias in Uganda. BMC geriatrics. 2024;24(1):602.

46. Ikanga J, Reyes A, Zhao L, Hill-Jarrett TG, Hammers D, Epenge E, et al. Exploring factors contributing to caregiver burden in family caregivers of congolese adults with suspected dementia. International journal of geriatric psychiatry. 2023;38(9):e6004.

47. Owokuhaisa J, Kamoga R, Musinguzi P, Muwanguzi M, Natukunda S, Mubangizi V, et al. Burden of care and coping strategies among informal caregivers of people with behavioral and psychological symptoms of dementia in rural south-western Uganda. BMC geriatrics. 2023;23(1):475.

48. Th´ebaut C, Houinato D, Gansou G, Gnonlonfoun D, Adoukonou T, Preux P, et al. Informal care time and costs of dementia care in Benin (West Africa). Neuroepidemiology. 2024.

49. Pokala S, Wandera SO, Kawooya S, Victoria AE, Odo LW, Nakasujja N, et al. Dementia knowledge and caregiver burden among carers of older adults in rural eastern Uganda. Alzheimer's & Dementia. 2025;21(8):e70587.

50. Musyimi CW, Ndetei DM, Evans-Lacko S, Oliveira D, Mutunga E, Farina N. Perceptions and experiences of dementia and its care in rural Kenya. Dementia. 2021;20(8):2802-19.

51. Okwaraji FE, Onyebueke GC, Nwakaego OE. Burn Out and Psychological Distress among Care Givers of Dementia Patients Attending the Out Patient Geriatric Clinics of Two Nigerian Tertiary Health Institutions. International Neuropsychiatric Disease Journal. 2023;20(4):15-23.

52. Jacobs R, Schneider M, Farina N, du Toit P, Docrat S, Comas-Herrera A, et al. Dementia in South Africa: a situational analysis. Dementia. 2024;23(3):452-75.

53. Walker J, Dotchin C, Breckons M, Fisher E, Lyimo G, Mkenda S, et al. Patient and caregiver experiences of living with dementia in Tanzania. Dementia. 2023;22(8):1900-20.

54. Ainamani HE, Bamwerinde WM, Rukundo GZ, Tumwesigire S, Kalibwani RM, Bikaitwaho EM, et al. Participation in gardening activity and its association with improved mental health among family caregivers of people with dementia in rural Uganda. Preventive medicine reports. 2021;23:101412.

55. Stuart-Röhm K, Clark IN, Baker FA. 'Caregivers' Experiences of a Singing Training Program to Support Person-Centered Dementia Care. Journal of Music Therapy. 2024;61(3):244-62.

56. Musyimi CW, Muyela LA, Ndetei DM, Evans-Lacko S, Farina N. Acceptability and feasibility of a community dementia stigma reduction program in kenya. Journal of Alzheimer's Disease. 2024;100(2):699-711.

57. Chikumbu EF, Bunn C, Kasenda S, Dube A, Phiri-Makwakwa E, Jani BD, et al. Experiences of multimorbidity in urban and rural Malawi: An interview study of burdens of treatment and lack of treatment. PLOS Global Public Health. 2022;2(3):e0000139.

58. Pedroza P, Miller-Petrie MK, Chen C, Chakrabarti S, Chapin A, Hay S, et al. Global and regional spending on dementia care from 2000–2019 and expected future health spending scenarios from 2020–2050: an economic modelling exercise. EClinicalMedicine. 2022;45.

59. Chen S, Cao Z, Nandi A, Counts N, Jiao L, Prettner K, et al. The global macroeconomic burden of Alzheimer's disease and other dementias: estimates and projections for 152 countries or territories. The Lancet Global Health. 2024;12(9):e1534-43.

60. Akinyemi RO, Yaria J, Ojagbemi A, Guerchet M, Okubadejo N, Njamnshi AK, et al. Dementia in Africa: current evidence, knowledge gaps, and future directions. Alzheimer's & Dementia. 2022;18(4):790-809.

61. Reid E, Ghoshal A, Khalil A, Jiang J, Normand C, Brackett A, et al. Out-of-pocket costs near end of life in low-and middle-income countries: a systematic review. PLOS Global Public Health. 2022;2(1):e0000005.

62. Miranda JJ, Lŭcar-Flores M, Bernabe-Ortiz A, Calvo RA, Cardenas MK, Custodio N, et al. Rethinking and transforming health systems for dementia care in low-and middle-income country settings. PLOS Global Public Health. 2025;5(12):e0005419.

63. Daliri DB, Afaya A, Laari TT, Abagye N, Aninanya GA. Exploring the burden on family caregivers in providing care for their mentally ill relatives in the Upper East Region of Ghana. PLOS Global Public Health. 2024;4(4):e0003075.

PRISMA-Sc

Click here to access/download

Supporting Information

PRISMA-ScR-Fillable-Checklist_10Sept2019.docx
