# Supplementary material for: Dementia care gaps in Sub-Saharan Africa: A scoping review of technology, environmental, community perspectives, and financial constraints
Source: PLOS Glob Public Health. 2026 Oct 6;6(10):e0007265. doi: 10.1371/journal.pgph.0007265 (PMC13641696; doi:10.1371/journal.pgph.0007265)
Supplement: Supplementary file 2 [file pgph.0007265.s005.pdf]

Reviewer comment

Manuscript Title: **Understanding Dementia Care Gaps in SSA: An Integrated Analysis of Technology, Social Determinants, Community Perspectives, and Financial Constraints**

Title:

A title with "SSA" may mean several things, and readers may not be able to capture the essence, scope, and focus of the paper at a glance.

- It would be appropriate to first mention "Sub-Saharan Africa" instead of "SSA".

You clearly indicated your review scope as "Technology, Social Determinants, Community Perspectives, and Financial Constraints."

- However, you discussed environmental factors… you need to clearly align your four domains as you've stated in the abstract.

- Suggested Title: Dementia Care Gaps in Sub-Saharan Africa: An Integrated Analysis of Technology, Environmental and Social Determinants, Community Perspectives, and Financial Constraints

Abstract:

"This review examined recent literature from 2021 to 2025 to identify gaps in dementia care across four key domains: environmental and social factors, technological capacity, community-based caregiving, and financial and economic factors".

-You have cited references over ten years old, which defiles what you've stated in your abstract. You may stick to current literature and ensure all references fall within the scope of your study.

- What is the originality of your study? Explain how this paper differs from previous published research in the subject area

Introduction:

Fig 2. "The green shading indicates the countries represented in the included literature, while the beige shading indicates the countries where no relevant studies were identified".

- Could you confirm if no studies or dementia cases are reported in beige-shading countries? If you found any, then you may reshape your assertion or claim.

- The map indicated more studies in South Africa (383). Yet your current study has few reports from South Africa; could you explain?

-You may consider a table detailing the countries and the number of reported dementia cases, OR you may consider selected sub-Saharan African countries.

2.2 Information Sources and Search Strategy

“The literature search was limited to the period from January 2021 to December 2025”.

- Your references should fall within this timeframe as you’ve clearly indicated.

Fig. 3: “summarizes the environmental, cultural, and social factors that contribute to disparities in dementia care across the SSA”.

- The figure and description below are not clear (clearly show the correlation or how each factor is linked to the other). Readers should be able to see the linkage. You may consider using a table

- How does a lack of neuroimaging fit into environmental, cultural, and social factors? You have discussed neuroimaging under technological barriers, so how do you explain it?

- Fig3. The title can be environmental and sociocultural challenges.

Fig 4. Technology gaps and limitations affecting dementia care.

- How does the numbering correlate to the factors?

Fig 5. Community-based dementia care and informal caregiving in SSA.

-Suggestion: You may consider a table summary of community-based epidemiological studies on dementia in SSA [Author, year, criteria, sample, prevalence, limitations)
