# Supplementary material for: Dementia care gaps in Sub-Saharan Africa: A scoping review of technology, environmental, community perspectives, and financial constraints
Source: PLOS Glob Public Health. 2026 Oct 6;6(10):e0007265. doi: 10.1371/journal.pgph.0007265 (PMC13641696; doi:10.1371/journal.pgph.0007265)
Supplement: Supplementary file 3 [file pgph.0007265.s009.pdf]

Response to Editor and Reviewers

Manuscript ID: PGPH-D-26-00901

Manuscript title: *Dementia Care Gaps in Sub-Saharan Africa: A Scoping Review of Technology, Environmental, Community Perspectives, and Financial Constraints*

Dear Reviewers and Editorial Team,

We sincerely thank the Academic Editor and all reviewers for their careful evaluation of our manuscript and for the constructive comments. We have revised the manuscript substantially to improve its methodological transparency, scoping-review framing, citation alignment, data availability, and presentation of included evidence. We have also updated the supplementary file to provide a complete evidence-extraction matrix for the 33 included studies. Below, we provide a point-by-point response to each journal requirement and reviewer comment.

Response to Journal Requirements

Comment: Please confirm whether the wording in the title is correct and add "scoping review" to the manuscript title.

Response: Thank you. We have revised the title to clearly identify the article as a scoping review. The revised title is: *Dementia Care Gaps in Sub-Saharan Africa: A Scoping Review of Technology, Environmental and Social Determinants, Community Perspectives, and Financial Constraints*. We will also ensure that the metadata for online submissions exactly matches the revised manuscript title.

Comment: Please provide a manuscript source file at revision.

Response: We have prepared the revised manuscript source file and will upload it in the required format as part of the revision package.

Comment: Please provide separate figure files in .tif or .eps format.

Response: Thank you for this instruction. We have prepared the revised figures as separate figure files in the required format. We will upload these files separately during resubmission, in accordance with PLOS figure-file requirements.

Comment: Supporting information files were uploaded, but a full list of legends was not included.

Response: We have added a Supporting Information legend for the supplementary evidence extraction file. The legend now reads: *S1 File. Evidence extraction matrix of included studies on dementia care gaps in Sub-Saharan Africa.* The supplementary file lists all 33 included studies and includes reference number, study author, year, country or region, study design, sample size or data scope, population type, thematic domain, key dementia care gaps or findings, and barrier or gap category.

Comment: Potential copyright issues were raised for the map figure and for clip-art or icons in other figures.

Response: Thank you for highlighting this important issue. To avoid inconsistency with the PRISMA flow diagram and to remove any uncertainty about the map base layer and map copyright permissions, we removed the previous geographic-distribution map from the revised manuscript. The remaining figures have been renumbered accordingly. For the revised conceptual figures, we have ensured that the graphical elements are either author-created or replaced with elements that are compatible with publication under the PLOS CC BY 4.0 license. We will provide the revised figure files separately with the resubmission.

Response to General Reviewer Questionnaire Items

Comment: Some reviewers indicated that the manuscript only partly met PLOS Global Public Health's publication criteria or required additional rigor and clarity.

Response: We appreciate this assessment and have revised the manuscript to more clearly meet the journal's expectations. We strengthened the scoping review framing, revised the title and abstract, expanded the Methods section, clarified the search strategy and eligibility criteria, added the study selection process, and included a detailed evidence extraction matrix as S1 File. We also revised the Results sections so that each thematic domain is grounded in the included studies rather than reading only as a narrative essay.

Comment: The statistical analysis was questioned by some reviewers.

Response: Because this manuscript is a scoping review, we did not conduct a meta-analysis or pooled statistical analysis. We have clarified that the synthesis was descriptive and thematic. We also clarified the screening process and added information on independent screening by reviewers and resolution of disagreements. Since the purpose of the review was to map evidence and identify care gaps, the revised manuscript avoids implying that quantitative effect-size synthesis was performed.

Comment: One reviewer indicated that the underlying data were not fully available.

Response: Thank you. We have addressed this by providing a complete supplementary evidence extraction matrix as S1 File. The file identifies all 33 included studies and summarizes the extracted information used to support the review findings. Since this is a review of published literature, the underlying evidence consists of publicly available published studies, and the extracted dataset is now provided in the supplementary file for transparency.

Comment: Reviewers generally indicated that the manuscript was intelligible and written in standard English, but some language and clarity issues were noted.

Response: We revised the manuscript for clarity, precision, and consistency. We reduced overgeneralized wording, improved alignment between claims and citations, clarified the Methods section,

and revised the abstract, introduction, discussion, and conclusion to improve flow and reduce redundancy.

Response to Reviewer 1

Comment: Figure 1 states that 33 studies were included, but the manuscript did not include a complete table of the 33 studies. The reviewer requested a comprehensive data extraction table listing all included studies.

Response: Thank you for this important suggestion. We have added a complete supplementary evidence extraction matrix as an S1 File. This file lists all 33 included studies, including the reference number, author, year, country or region, study design, sample size or data scope, population type, thematic domain, key dementia care gaps or findings, and barrier or gap category. This addition allows readers to independently verify the dataset, assess geographic coverage, and understand the types of evidence that informed the thematic synthesis.

Comment: The previous map figure contradicted the PRISMA diagram because the map appeared to show broad country-level numbers that did not correspond to the 33 included studies.

Response: We agree with this concern. The previous map figure created confusion because the country counts could be interpreted as representing the 33 included studies in the review. To avoid this inconsistency, we removed the map from the revised manuscript. The revised manuscript now relies on the PRISMA flow diagram, Table 1, and S1 File to describe the included evidence. The remaining figures have been renumbered accordingly.

Comment: The Methods section did not explain whether screening was independently conducted by two or more reviewers, or how disagreements were resolved.

Response: Thank you. We revised the Methods section to clarify the study-selection process. The revised manuscript now states that all records were imported into a reference management file, duplicates were removed, and study selection was conducted in two stages. First, four reviewers independently screened titles and abstracts against the eligibility criteria. Second, full texts were independently assessed by the same reviewers. Disagreements were resolved through discussion and consensus, and a fifth reviewer was available for final decision-making if consensus could not be reached.

Comment: Sections 3 through 6 read more like a narrative essay than a scoping review synthesis. The reviewer suggested quantifying findings at the beginning of each thematic section.

Response: We agree and have revised the Results sections to better ground the thematic synthesis in the included dataset. We added Table 1, which summarizes the number of studies represented in each thematic domain and the main countries or regions represented. We also added opening sentences to Sections 3 through 6 that report the number of included studies contributing to each domain. Specifically, the revised manuscript states that 5 studies addressed environmental and social challenges, 12 addressed technology-related challenges, 15 addressed community-based de-

mentia care and informal caregiving challenges, and 5 addressed financial and economic challenges. We also clarified that studies could contribute to more than one thematic domain, so the domain totals exceed 33.

Comment: The data availability statement was insufficient without a supplementary table detailing the 33 publications.

Response: Thank you. We have addressed this by providing S1 File, a full evidence extraction matrix of the 33 included studies. This file makes the review dataset transparent and allows readers to verify which publications were included in the synthesis.

Comment: The sentence in Section 2.5 stating that the synthesis differs from quantitative synthesis was unnecessary.

Response: We removed this unnecessary sentence and revised the synthesis section to more directly describe the descriptive and thematic approach used in the scoping review. The revised text now focuses on how extracted data were categorized into health-system capacity gaps, caregiving support gaps, service delivery and access gaps, and technology and innovation gaps.

Response to Reviewer 2

Comment: The reviewer recognized the scholarly value of the manuscript but noted that the manuscript needed stronger methodological transparency, clearer search strategy, better inclusion protocols, and stronger reproducibility safeguards.

Response: We sincerely thank the reviewer for this constructive assessment. We have substantially revised the Methods section to improve transparency and reproducibility. The revised manuscript now clearly identifies the study as a scoping review, reports that the review followed PRISMA-ScR guidance, lists the databases searched, specifies the search period from January 2021 to December 2025, and provides the main Boolean search string. We also clarified that Google Scholar, website searches, and citation tracking were used as supplementary sources. In addition, we expanded the eligibility criteria and study selection process, including independent title/abstract and full-text screening by four reviewers and consensus-based resolution of disagreements.

Comment: The reviewer noted that some arguments were presented without adequate empirical support and that citations needed stronger alignment with claims.

Response: We agree and have revised the manuscript to improve citation alignment. In the Introduction, we revised broad statements about dementia facilities and care gaps so that the citations now support the claims. We also replaced or repositioned citations where needed, particularly in the early background section. Throughout the manuscript, we attempted to avoid unsupported generalizations and to connect claims more directly to the included studies or to broader contextual sources.

Comment: The reviewer noted redundancy across the abstract, discussion, and conclusion.

Response: Thank you. We revised these sections to reduce repetition and improve focus. The abstract now clearly states the scoping review purpose and the four domains. The Discussion now synthesizes the implications of the findings across technology, community care, financing, and health-system capacity. The Conclusion was rewritten to emphasize key implications, including national dementia policies, environmental and social determinants, primary-care integration, caregiver training, affordable digital health solutions, financial protection, and region-specific dementia data infrastructure.

Comment: The reviewer noted that the manuscript should be more technically consistent and precise.

Response: We revised terminology throughout the manuscript to improve consistency. We now consistently refer to the work as a scoping review, use the four thematic domains throughout the manuscript, and align the domains with the WHO health-system framework. We also added Table 1 and the S1 File to connect the thematic findings to the extracted evidence.

Response to Reviewer 3

Comment: The reviewer recommended major revision and emphasized three main issues: the conflict between Figure 1 and the map figure, the need for a comprehensive table of included studies, and clarification of the narrow temporal scope of the search strategy.

Response: We thank the reviewer for these clear recommendations. First, we removed the previous map figure because it conflicted with the PRISMA diagram and could be misinterpreted as showing the distribution of the 33 included studies. Second, we added the S1 File, which provides a comprehensive evidence-extraction matrix for all 33 included studies. Third, we clarified the temporal scope of the review in the Methods section. The revised manuscript now states that the search focused on studies published between January 2021 and December 2025. The five-year window was selected to capture the most recent evidence during a period of growing awareness of population aging and dementia-care needs in SSA, which has led to increased scholarly attention to this topic in recent years. We also clarified in the Introduction that earlier landmark reports and analyses were cited only for epidemiological context and conceptual framing, not as part of the recently included study dataset.

Response to Reviewer 4

Comment: The reviewer expressed concern about the scope used in the study, how it was reflected in the map and data, and how linguistic diversity across SSA was considered during the identification of literature.

Response: Thank you for this important point. We have clarified the scope of the review and removed the previous map figure to avoid giving the impression of inaccurate geographic coverage.

The revised manuscript now uses the PRISMA flow diagram, Table 1, and S1 File to show how the included evidence was identified and categorized. We also clarified the temporal scope of the review as January 2021 to December 2025. We acknowledge that SSA is linguistically diverse and that relevant work may appear in English, French, Portuguese, and other languages. We have therefore revised the manuscript to clarify the search scope and acknowledge that the findings are based on studies retrieved through the selected databases and search strategy. This limitation is important because language and indexing differences may affect which SSA evidence is visible in international databases.

Response to Reviewer 5

Comment: The reviewer noted that some in-text citations did not fully support the claims to which they were attached, especially in the Introduction.

Response: Thank you for identifying this issue. We reviewed and revised the Introduction to align citations. In particular, we revised the sentence that previously suggested that citations 14–18 directly discussed dementia facilities. The revised text now more accurately states that recent studies from Ethiopia, Ghana, Kenya, Nigeria, South Africa, and Tanzania examined dementia-related care gaps in SSA. We also revised citation 19 to better support the relevant discussion of socioeconomic and financial burdens.

Comment: The reviewer suggested that the manuscript should acknowledge the need to understand local beliefs about aging and avoid assuming that Western biomedical concepts of dementia can be directly transferred into SSA contexts.

Response: We agree fully and have added this point to the technology-related challenges section. The revised manuscript now states that efforts to improve dementia screening and diagnosis in SSA should not assume that dementia screening tools and care pathways can be transferred without local adaptation. We added discussion of local beliefs about aging, memory loss, family responsibility, and what constitutes normal versus pathological cognitive decline. We also emphasized that culturally grounded approaches, developed with communities, caregivers, and frontline health workers, may reduce stigma, improve acceptability, and ensure that screening tools and care pathways are locally appropriate.

Response to Reviewer 6

Comment: The reviewer stated that dementia is becoming a major public health problem in SSA and that the manuscript highlights four critical dementia care gaps.

Response: We thank the reviewer for the positive assessment. We have retained the four-domain structure of the manuscript while strengthening the scoping-review framing, methods, evidence table, and discussion of policy implications.

Response to Reviewer 7

Comment: The reviewer noted that a statistical section was not included in the manuscript, although the manuscript was otherwise well written and satisfied publication criteria.

Response: Thank you. We clarified that this manuscript is a scoping review and does not conduct a meta-analysis or pooled statistical analysis. Therefore, a conventional statistical analysis section was not appropriate. Instead, we strengthened the Methods section by describing the descriptive and thematic synthesis approach and clarifying the study selection process. We also added Table 1 and S1 File to make the evidence base and thematic categorization transparent.

Closing Statement

We again thank the Academic Editor and reviewers for their valuable feedback. The revised manuscript has been strengthened by a clearer scoping-review title, expanded methodology, improved citation alignment, removal of the inconsistent map figure, addition of Table 1, addition of an S1 File containing all 33 included studies, clarification of the temporal scope, and revisions to the discussion and conclusion. We believe these changes substantially improve the rigor, transparency, and readability of the manuscript.

Sincerely,
The Authors
