# Supplementary material for: Dementia care gaps in Sub-Saharan Africa: A scoping review of technology, environmental, community perspectives, and financial constraints
Source: PLOS Glob Public Health. 2026 Oct 6;6(10):e0007265. doi: 10.1371/journal.pgph.0007265 (PMC13641696; doi:10.1371/journal.pgph.0007265)
Supplement: Supplementary file 4 [file pgph.0007265.s001.docx]

# **S1 Checklist. PRISMA-ScR Checklist**

*Dementia Care Gaps in Sub-Saharan Africa: A Scoping Review of Technology, Environmental, Community Perspectives, and Financial Constraints*

This checklist is derived from the PRISMA-ScR (Preferred Reporting Items for Systematic Reviews and Meta-Analyses extension for Scoping Reviews) checklist, adapted from the PRISMA 2020 Statement (Tricco AC, Lillie E, Zarin W, et al. PRISMA Extension for Scoping Reviews (PRISMA-ScR): Checklist and Explanation. Ann Intern Med. 2018;169(7):467–473). The PRISMA 2020 Statement is distributed under the terms of the Creative Commons Attribution License (CC BY 4.0); see https://www.prisma-statement.org/citing-prisma-2020 for citation guidance.

| **Section** | **Item** | **PRISMA-ScR checklist item** | **Reported on page / section** |
| --- | --- | --- | --- |
| **TITLE** | | | |
| Title | 1 | Identify the report as a scoping review. | Title page and manuscript title: “Dementia Care Gaps in Sub-Saharan Africa: A Scoping Review of Technology, Environmental, Community Perspectives, and Financial Constraints.” |
| **ABSTRACT** | | | |
| Structured summary | 2 | Provide a structured summary that includes background, objectives, eligibility criteria, sources of evidence, charting methods, results, and conclusions. | Abstract (p. 1). Note: currently written as a single narrative paragraph rather than with explicit subheadings; content covers background, objective, methods, and conclusions. |
| **INTRODUCTION** | | | |
| Rationale | 3 | Describe the rationale for the review in the context of what is already known. | Introduction, paragraphs 1–2. |
| Objectives | 4 | Provide an explicit statement of the questions and objectives being addressed. | Introduction, final paragraph (p. 2): states the four thematic domains and the review's objectives. |
| **METHODS** | | | |
| Protocol and registration | 5 | Indicate whether a review protocol exists, if and where it can be accessed, and if available, provide registration information. | Not reported. No protocol registration (e.g., OSF) is described in the manuscript. |
| Eligibility criteria | 6 | Specify characteristics of the sources of evidence used as eligibility criteria, and rationale. | Methods, Section 2.3 “Eligibility Criteria” (inclusion/exclusion criteria listed). |
| Information sources | 7 | Describe all information sources in the search and where and when the last search was executed. | Methods, Section 2.2 “Information Sources and Search Strategy.” Search date range (Jan 2021–Dec 2025) given; exact search execution date not stated. |
| Search | 8 | Present the full electronic search strategy for at least one database. | Methods, Section 2.2: full Boolean search string provided. |
| Selection of sources of evidence | 9 | State the process for selecting sources of evidence. | Methods, Section 2.4 “Study Selection Process” (two-stage screening, four independent reviewers, Fleiss’ kappa, consensus/fifth-reviewer arbitration). |
| Data charting process | 10 | Describe the methods of charting data and processes for obtaining/confirming data from investigators. | Methods, Section 2.5 “Synthesis of Results” references the extraction/charting process; the underlying extraction matrix is provided as S1 Table. Consider adding whether charting was piloted and independently duplicated. |
| Data items | 11 | List and define all variables for which data were sought. | See column headers of S1 Table (study design, sample, population, thematic domain, key findings, barrier category); not separately enumerated in Methods text. |
| Critical appraisal of individual sources of evidence | 12 | If done, provide a rationale for conducting a critical appraisal, and describe the methods used. | Not performed (optional item for scoping reviews; no formal risk-of-bias/quality appraisal was conducted). |
| Synthesis of results | 13 | Describe the methods of handling and summarizing the data that were charted. | Methods, Section 2.5 (descriptive and thematic synthesis across four domains). |
| **RESULTS** | | | |
| Selection of sources of evidence | 14 | Give numbers of sources of evidence screened, assessed for eligibility, and included, with reasons for exclusions, ideally with a flow diagram. | Fig 1 (PRISMA flow diagram) and Methods, Section 2.4. |
| Characteristics of sources of evidence | 15 | For each source of evidence, present characteristics for which data were charted. | Table 1 (summary by thematic domain/country) and S1 Table (full study-level characteristics). |
| Critical appraisal within sources of evidence | 16 | If done, present data on critical appraisal of included sources of evidence. | Not applicable — no critical appraisal conducted (see item 12). |
| Results of individual sources of evidence | 17 | For each included source of evidence, present the relevant data extracted. | Results sections 3–6 (narrative synthesis by domain) and S1 Table. |
| Synthesis of results | 18 | Summarize and/or present the charting results as they relate to the review question(s) and objectives. | Results sections 3–6, each closing with a synthesis paragraph. |
| **DISCUSSION** | | | |
| Summary of evidence | 19 | Summarize the main results, link to the review questions/objectives, and consider the relevance to key groups. | Discussion, paragraphs 1–3. |
| Limitations | 20 | Discuss the limitations of the scoping review process. | Discussion, final paragraph. |
| Conclusions | 21 | Provide a general interpretation of the results with respect to the review questions and objectives, and suggest next steps. | Conclusion section. |
| **FUNDING** | | | |
| Funding | 22 | Describe sources of funding for the included sources of evidence, as well as sources of funding for the scoping review; describe the role of the funders. | Addressed in the separate Financial Disclosure statement submitted with this manuscript (“The authors received no specific funding for this work”). |
