# Supplementary material for: Dementia care gaps in Sub-Saharan Africa: A scoping review of technology, environmental, community perspectives, and financial constraints
Source: PLOS Glob Public Health. 2026 Oct 6;6(10):e0007265. doi: 10.1371/journal.pgph.0007265 (PMC13641696; doi:10.1371/journal.pgph.0007265)
Supplement: Supplementary file 5 [file pgph.0007265.s002.docx]

**S1 Table. Evidence extraction matrix of the 33 included studies on dementia care gaps in Sub-Saharan Africa.**

| **Study ID** | **Reference number** | **Study** | **Year** | **Country/Region** | **Study Design** | **Sample Size / Data Scope** | **Population Type** | **Thematic domain** | **Key Dementia Care Gaps / Findings** | **Barrier / Gap Category** |
| --- | --- | --- | --- | --- | --- | --- | --- | --- | --- | --- |
| 1 | [25] | Wakawa et al. | 2025 | Northeastern Nigeria | Retrospective hospital-based analysis | 1,216 dementia cases aged 60 years and older | Older adults diagnosed with dementia | Environmental and social challenges; Technology-related challenges | Documented dementia burden at the only neuropsychiatric facility in Northeastern Nigeria. Findings show dependence on limited specialized facilities and the need for broader diagnostic and service capacity. | Specialist-service gap; diagnostic delay; facility concentration; data gap |
| 2 | [26] | Wisdom et al. | 2025 | Nigeria | Literature-based policy analysis | Literature and policy synthesis | People with neurodegenerative diseases, including dementia | Environmental and social challenges; Technology-related challenges | Identified inadequate infrastructure, few specialists, high care costs, cultural misconceptions, limited rural access, insufficient research funding, and lack of epidemiological data. | Infrastructure gap; workforce shortage; cost barrier; research funding gap |
| 3 | [27] | Ssonko et al. | 2023 | South Africa | Retrospective memory-clinic cohort study | 165 memory-clinic patients aged 60 years and older | Older adults with dementia | Environmental and social challenges | Reported dementia subtypes, cognitive decline, and survival among memory-clinic patients. Findings indicate the need for longitudinal clinic data and earlier diagnosis in African memory-clinic settings. | Memory-clinic capacity; late diagnosis; longitudinal data gap |
| 4 | [28] | Bosire et al. | 2025 | Kenya | Ethnographic qualitative study | 21 healthcare providers | Healthcare providers involved in AD/ADRD diagnosis and care | Environmental and social challenges; Community-based dementia care and informal caregiving challenges | Identified gaps in early detection due to lack of equipment, inadequate or untrained providers, high diagnosis and care costs, limited tools, stigma, specialist shortages, and lack of clear care pathways. | Workforce gap; diagnostic-equipment gap; cost barrier; stigma; policy gap |
| 5 | [29] | Beyene and Berha | 2023 | Sub-Saharan Africa | Systematic review | 8 studies; 2,781 patients | People with dementia and HIV-associated dementia | Environmental and social challenges | Reviewed management practice and clinical outcomes. Cognitive stimulation therapy and early antiretroviral therapy showed benefits, but available intervention evidence remained limited. | Treatment-evidence gap; intervention gap; low-resource care model |
| 6 | [30] | Alemayehu et al. | 2025 | Sub-Saharan Africa | Narrative literature review | Literature synthesis | People living with dementia in SSA | Technology-related challenges; Financial and economic challenges | Discussed risk factors, public perception, and management approaches. Highlighted myths, stigma, data drought, limited diagnosis, and the need for national dementia plans and culturally adapted interventions. | Data gap; stigma; diagnostic gap; policy gap; culturally adapted care |
| 7 | [31] | Oyinlola | 2024 | Nigeria | Policy analysis and literature-based review | Policy and literature synthesis | People living with dementia and caregivers | Technology-related challenges | Argued that Nigeria lacks a coherent national dementia strategy. Highlighted stigma, weak caregiver support, limited information systems, and the need to integrate dementia into health and social care systems. | Policy gap; national-strategy gap; stigma; caregiver support; information systems |
| 8 | [32] | Musyimi et al. | 2023 | Kenya | Qualitative vignette and stakeholder care-pathway study | 29 stakeholders | Stakeholders and community dementia-care pathway actors | Technology-related challenges | Identified four initial care responses: perceiving symptoms as normal aging, consulting spiritual or traditional healers, visiting private or primary care, or taking no action. | Care-pathway gap; cultural belief; delayed care; awareness gap |
| 9 | [33] | Mwendwa et al. | 2021 | Kenya | Interpretive phenomenological qualitative study | 10 caregivers | Family caregivers of people living with dementia | Technology-related challenges | Caregivers described dementia care as stressful and continuous, with emotional, financial, practical, and knowledge-related challenges. | Caregiver burden; financial stress; knowledge gap; support-service gap |
| 10 | [34] | Aballa et al. | 2025 | Kenya | Ethnographic qualitative study | 5 focus-group discussions with 35 participants; additional interviews NR | Rural community members | Technology-related challenges | Found that older people with dementia may be perceived as witches and exposed to stigma or harm. Highlights the need for public education and protection. | Stigma; cultural misconception; community safety; awareness gap |
| 11 | [35] | Yenesew et al. | 2024 | East Africa | Scoping review of population-based studies | 7 included studies after screening 445 publications | Older adults in East Africa | Technology-related challenges | Found very limited population-based evidence, including four studies on dementia prevalence and three on cognitive impairment. Called for more longitudinal and population-based research and stronger regional evidence generation. | Epidemiological data gap; research-capacity gap; regional evidence gap |
| 12 | [36] | Aryee | 2024 | Ghana | Systematic review of adult day-center literature | Literature on adult day-center models | People with dementia in Ghana | Technology-related challenges | Reviewed the need for culturally appropriate adult day centers. Identified stigma, lack of infrastructure, caregiver unpreparedness, and lack of respite services. | Formal-care infrastructure gap; respite-care gap; stigma; caregiver training |
| 13 | [37] | Mahomed and Pretorius | 2022 | South Africa | Qualitative study | NR | Family caregivers of individuals with dementia | Technology-related challenges | Explored lived experiences of dementia caregiving in a township context. Findings highlight emotional, social, practical, and support-service challenges for caregivers. | Caregiver burden; township service gap; psychosocial support gap |
| 14 | [38] | Paddick et al. | 2021 | Tanzania | Validation and feasibility study | 3,011 screened; 610 clinically assessed | Older adults aged 60 years and older in rural Tanzania | Technology-related challenges | Tested app-based dementia screening by lay health workers. Demonstrated the potential of digital screening to address specialist shortages in low-resource settings. | Technology gap; workforce shortage; screening accessibility |
| 15 | [39] | Mwendwa et al. | 2022 | Sub-Saharan Africa | Systematic review and narrative synthesis | 19 studies; 378 caregivers | Family and informal caregivers | Technology-related challenges | Synthesized six themes: conceptualizing dementia, caregiving arrangements, caregiving impact, caregiver identity, managing care, and unmet needs. Called for better information campaigns and support programs. | Informal-care burden; unmet support needs; caregiver training gap |
| 16 | [40] | Ayisi-Boateng et al. | 2022 | Ghana: Kumasi | Multicentre cross-sectional needs-assessment study | 50 carers | Adult carers of people previously diagnosed with dementia | Community-based dementia care and informal caregiving challenges | Identified substantial unmet caregiver needs, particularly for clear information about dementia, treatment, and crisis management. Findings support accessible education, counselling, and practical caregiver assistance. | Caregiver-information gap; unmet support needs; caregiver-training gap |
| 17 | [41] | Kamoga et al. | 2023 | Uganda: rural Southwestern Uganda | Population-based quantitative cross-sectional study | 175 caregivers of people living with dementia | Family caregivers of people living with dementia | Community-based dementia care and informal caregiving challenges | Almost all caregivers reported at least one behavioral or psychological symptom of dementia. Frequent and severe symptoms caused substantial caregiver distress, demonstrating the need for early BPSD screening and caregiver support. | BPSD-management gap; caregiver distress; early-screening gap; psychosocial-support gap |
| 18 | [42] | Gumikiriza-Onoria et al. | 2024 | Uganda: Wakiso District | Cross-sectional caregiver mental-health study using purposive sampling | 90 family caregivers selected from a pre-existing cohort of 500 individuals | Family caregivers of people with Alzheimer’s disease and related dementias | Community-based dementia care and informal caregiving challenges | Psychological distress and depression each affected 64.4% of caregivers. Findings demonstrate the need for structured mental-health services, caregiver-support systems, and gender-responsive interventions. | Caregiver mental-health burden; depression; formal-support gap; gender-responsive care gap |
| 19 | [43] | Ikanga et al. | 2023 | Democratic Republic of the Congo | Pilot cross-sectional caregiver-burden study | 30 patient-caregiver dyads | Family caregivers of adults with suspected dementia | Community-based dementia care and informal caregiving challenges | Caregiver burden was associated with care-recipient cognitive impairment, functional decline, frailty, depression, agitation, and other neuropsychiatric symptoms. Findings highlight the need for family support and symptom-management services. | Caregiver burden; neuropsychiatric-symptom management gap; family-support gap |
| 20 | [44] | Owokuhaisa et al. | 2023 | Uganda | Cross-sectional qualitative study using purposive sampling, in-depth interviews, and thematic content analysis | 27 informal caregivers | Informal caregivers of older people with behavioral and psychological symptoms of dementia (BPSD) | Community-based dementia care and informal caregiving challenges | Caregivers experienced substantial financial, physical, psychological, and social burdens. Common coping strategies included religious coping, acceptance, emotional and social support, planning, persistence, and self-distraction. | Caregiver burden; financial strain; psychosocial burden; limited caregiver and health-system support |
| 21 | [45] | Thébaut et al. | 2024 | Benin | Cost-of-illness study using hospital and community recruitment | 135 older individuals with varying dementia stages | Older adults with dementia and informal caregivers | Community-based dementia care and informal caregiving challenges | Caregivers provided up to eight hours of care daily. Mean annual dementia-care costs were approximately PPP$2,399.66, and informal care represented up to 92% of total costs. | Informal-care burden; time burden; financial burden; economic-data gap |
| 22 | [46] | Pokala et al. | 2025 | Uganda: rural Eastern Uganda | Cross-sectional caregiver-older-adult dyadic survey | 602 caregiver-older-adult dyads interviewed; 598 matched dyads included in the final analysis | Carers of older adults in rural communities | Community-based dementia care and informal caregiving challenges | Caregiver burden prevalence was 28.9%. In adjusted analyses, greater dementia knowledge and caregiver depression were associated with caregiver burden; among caregivers of older adults with dementia, caregiver depression and older caregiver age were associated with burden. | Caregiver burden; caregiver mental-health gap; dementia-education gap; rural-support gap |
| 23 | [47] | Musyimi et al. | 2021 | Kenya: rural communities | Qualitative study using focus-group discussions and individual interviews | 38 key stakeholders in focus groups plus 5 individual caregiver interviews | Carers, healthcare providers, and members of the general public | Community-based dementia care and informal caregiving challenges | Identified negative stereotypes, limited dementia knowledge, fragmented diagnostic pathways, neglect, and abuse. Poor awareness and inadequately equipped healthcare systems reinforced stigma and delayed care. | Cultural misconception; stigma; knowledge gap; diagnostic-pathway gap |
| 24 | [48] | Okwaraji et al. | 2023 | Nigeria: Enugu, Southeast Nigeria | Descriptive cross-sectional study | 460 caregivers | Caregivers of dementia patients attending two tertiary geriatric clinics | Community-based dementia care and informal caregiving challenges | Psychological distress affected 39.6% of caregivers, while 23.9% experienced emotional exhaustion. Findings support institutional assistance, caregiver incentives, and community caregiving centres. | Caregiver burnout; psychological distress; institutional-support gap; financial support need |
| 25 | [49] | Jacobs et al. | 2024 | South Africa | Three-phase national situational analysis | Desk review; multisectoral stakeholder interviews; and SWOT analysis | People living with dementia, families, and service providers | Community-based dementia care and informal caregiving challenges | Identified structural barriers to dementia diagnosis, care, and support, along with gaps and opportunities in existing service provision. The study emphasized the need for coordinated intersectoral policy responses. | Health-system fragmentation; policy gap; diagnostic gap; service-access gap |
| 26 | [50] | Walker et al. | 2023 | Tanzania | Qualitative study using semi-structured interviews and framework analysis | 14 people with dementia and 12 caregivers; 26 participants total | People living with dementia and their caregivers | Community-based dementia care and informal caregiving challenges | Participants described social isolation, stigma, cultural misconceptions, limited formal healthcare, and insufficient caregiver knowledge and support. These challenges affected the physical and mental health of people with dementia. | Stigma; caregiver-knowledge gap; formal-care gap; service-access gap |
| 27 | [51] | Ainamani et al. | 2021 | Uganda | Quantitative cross-sectional survey study | 242 family caregivers; 131 participated in gardening and 111 did not | Family caregivers of older people living with dementia who had provided care for more than six months | Community-based dementia care and informal caregiving challenges | Caregivers reported substantial burden and high levels of depression, anxiety, and stress. Participation in gardening was associated with lower symptoms of depression, anxiety, and stress. | Caregiver mental-health burden; psychosocial-support gap; community-based intervention gap |
| 28 | [52] | Stuart-Röhm et al. | 2024 | South Africa | Participatory action-research study using iterative training cycles and qualitative interviews | 8 caregivers across 2 care homes; 4 iterative participatory cycles | Formal caregivers of people living with dementia | Community-based dementia care and informal caregiving challenges | Person-centered caregiver-singing training increased dementia knowledge, emotional well-being, confidence, self-awareness, and self-efficacy. Findings highlight the need for sustainable and scalable caregiver training. | Caregiver-training gap; psychosocial-intervention gap; person-centered-care gap |
| 29 | [53] | Musyimi et al. | 2024 | Kenya | Community intervention feasibility study | NR | Community members and dementia-care stakeholders | Community-based dementia care and informal caregiving challenges | Examined acceptability and feasibility of a community dementia stigma-reduction program. Highlights the importance of community-level education to reduce stigma and improve care-seeking. | Stigma; awareness gap; community intervention gap |
| 30 | [54] | Pedroza et al. | 2022 | Sub-Saharan Africa (regional estimates within a global analysis) | Global and regional economic modelling study based on a systematic review of dementia-care spending | Dementia spending estimates for 2000-2019; projected health-spending scenarios for 2020-2050 | People living with dementia and health-financing systems | Financial and economic challenges | Estimated dementia-attributable spending in SSA at US$552 million in 2019, projected to rise to US$6,955 million by 2050. Reliance on modelled estimates highlights the scarcity of primary dementia-cost data from SSA. | Economic-data gap; primary-cost-data gap; health-financing gap; rising expenditure burden |
| 31 | [55] | Chen et al. | 2024 | Sub-Saharan Africa (regional estimates within a global analysis) | Health-augmented macroeconomic modelling study | 152 countries or territories; economic-burden estimates and projections for 2020-2050 | People living with dementia, informal caregivers, and national economies | Financial and economic challenges | Estimated the economic burden of Alzheimer’s disease and other dementias in SSA at approximately 158 billion international dollars during 2020-2050. The model incorporated morbidity, mortality, formal-care costs, and reductions in caregivers’ labor supply. | Macroeconomic burden; informal-care productivity loss; indirect-cost data gap; financing and planning gap |
| 32 | [56] | Akinyemi et al. | 2022 | Africa | Narrative evidence review | Review of African dementia evidence and knowledge gaps | People living with dementia in Africa | Financial and economic challenges | Reported wide variation in dementia prevalence and major knowledge gaps. Highlighted the need for longitudinal studies, culturally sensitive cognitive tools, biomarkers, genomics, coordinated African dementia research capacity, and better cost evidence. | Research gap; diagnostic-tool gap; biomarker gap; data gap; economic-data gap |
| 33 | [57] | Reid et al. | 2022 | Low- and middle-income countries; included Kenya and Tanzania among country examples | Systematic review | 12 included studies after screening 9,343 records | Adults near end of life or with serious life-limiting illness, including advanced Alzheimer’s disease and related dementias; patients, families, and caregivers | Financial and economic challenges | Reported that out-of-pocket costs increase with advanced illness and disease severity. Main cost drivers included medications and hospitalizations. High costs were associated with financial catastrophe, lost income, debt/borrowing, foregone education, and pressure on households. The review highlights the need for financial protection policies and further research on palliative and end-of-life care economics in LMICs. | Out-of-pocket financing; catastrophic health expenditure; financial-protection gap; palliative/end-of-life care financing gap; household economic burden |

*Note. NR = not reported or not clearly available in the extracted material. Reference numbers correspond to the manuscript reference list.*
