# Supplementary material for: Dementia care gaps in Sub-Saharan Africa: A scoping review of technology, environmental, community perspectives, and financial constraints
Source: PLOS Glob Public Health. 2026 Oct 6;6(10):e0007265. doi: 10.1371/journal.pgph.0007265 (PMC13641696; doi:10.1371/journal.pgph.0007265)
Supplement: Supplementary file 6 [file pgph.0007265.s004.docx]

**Reviewer Report**

**Journal:** PLOS Global Public Health

**Manuscript Number:** PGPH-D-26-00901

**Title:** Understanding Dementia Care Gaps in SSA: An Integrated Analysis of Technology, Social Determinants, Community Perspectives, and Financial Constraints

**1. Does this manuscript meet PLOS Global Public Health’s publication criteria? Is the manuscript technically sound, and do the data support the conclusions?**

No, not in its current form. While the topic is highly relevant and the conceptual framework (WHO Health Systems Building Blocks) is appropriate, there are major technical inconsistencies that undermine the manuscript’s rigor:

Inconsistency in Study Selection (Critical Flaw): There is a massive discrepancy between the PRISMA flow diagram (Figure 1) and the Geographic Distribution map (Figure 2). Figure 1 states that only 33 studies were included in the final review. However, Figure 2 shows 383 studies for South Africa, 31 for Kenya, and 11 for Namibia, among others. If the numbers in Figure 2 represent "included studies" (as the caption states), the total would exceed 450, contradicting the PRISMA results. This suggests either an error in data reporting or the use of an incorrect figure from a different study.

**Temporal Scope Issues:** The manuscript claims to review literature from 2021 to 2025. Given that we are currently in the early part of this range (or looking at a "future-dated" footer of March 2026), the inclusion of 2025 data is speculative. Furthermore, for a scoping review, a 4-year window is exceptionally narrow and likely excludes foundational evidence necessary to define "gaps."

**Technical Soundness:** Because of the discrepancy in the number of studies, it is impossible to verify if the "gaps" identified are truly representative of the 33 studies or if the authors are over-generalizing from a larger, unlisted dataset.

**2. Has the statistical analysis been performed appropriately and rigorously?**

Not Applicable / No. As this is a scoping review, traditional primary statistical analysis is not expected. However, thematic and descriptive synthesis must be rigorous.

The synthesis lacks a Summary of Evidence Table (typically "Table 1" in reviews), which should list each of the 33 included studies, their methodology, country of origin, and key findings. Without this, the synthesis is purely narrative and lacks the transparency required for a systematic scoping review.

The "Synthesis of Results" (Section 2.5) mentions descriptive analysis, but the manuscript does not provide any quantitative breakdown (e.g., "X% of studies focused on mHealth," "Y% identified stigma as a primary barrier").

**3. Have the authors made all data underlying the findings in their manuscript fully available?**

Partially.

The authors state that all data are publicly available as they are based on peer-reviewed publications.

However, to comply with PLOS Data Policy, authors should provide the Data Extraction Table (the spreadsheet used to categorize the 33 studies). This allows other researchers to see exactly how each study was coded into the four domains (Social, Technological, Community, Financial). This table is currently missing from the manuscript and supporting information.

**4. Is the manuscript presented in an intelligible fashion and written in standard English?**

Yes.

The manuscript is written in high-quality academic English. The arguments are clear, the structure is logical, and the terminology is generally consistent with global health standards.

**Minor Presentation Errors:**

The footer date "March 4, 2026" is likely a template error and should be corrected to the actual date of submission.

The "Manuscript Number" and "Manuscript Draft" headers indicate this is a direct export from a submission system, which is acceptable, but the internal inconsistencies in the figures (as noted in Question 1) significantly hinder the "intelligibility" of the research findings.

**Summary Recommendation:**

The manuscript requires Major Revision. The authors must resolve the conflict between Figure 1 and Figure 2, provide a comprehensive table of included studies, and clarify the narrow temporal scope of their search strategy to ensure the "gaps" identified are methodologically grounded.
