# Supplementary material for: Dementia care gaps in Sub-Saharan Africa: A scoping review of technology, environmental, community perspectives, and financial constraints
Source: PLOS Glob Public Health. 2026 Oct 6;6(10):e0007265. doi: 10.1371/journal.pgph.0007265 (PMC13641696; doi:10.1371/journal.pgph.0007265)
Supplement: Supplementary file 7 [file pgph.0007265.s006.docx]

General Assessment

This manuscript addresses a significant and timely issue, namely dementia care gaps in Sub-Saharan Africa (SSA), through a multi-domain framework encompassing social, technological, caregiving, and financial aspects. This topic holds considerable relevance to global public health, particularly in light of demographic transitions and the increasing burden of non-communicable diseases in SSA. However, it currently lacks the methodological rigor and reporting standards expected of a scoping review.

**Major Comments**

1. Methodological Rigor and Reporting (Critical Issue):

- Recommendation:

- Clarify whether this is a true scoping review (mapping only) or a systematic review

2. Conceptual Clarity and Framework Use

- Explicitly map each domain to WHO building blocks in a table or figure- Avoid conceptual overlap (e.g., workforce vs technology constraints)

3. Overinterpretation and Lack of Evidence Weighting

The manuscript frequently:

Recommendation:

- Acknowledge heterogeneity more explicitly- Avoid overgeneralization- Distinguish between evidence-based findings and author interpretation

4. Geographic Representation Bias.

Recommendation:

- Provide a clearer breakdown of country representation- Explicitly discuss regional evidence gaps

The abstract:

- Lacks detailed methodology- Omits the number of included studies- Resembles a narrative summary

Recommendation: Revise to include

- Study design (scoping review)- Databases searched- Number of included studies- Clear objective

6. Data Availability Statement (Needs Revision)

The statement “All data are publicly available as this is a review” is insufficient.

Recommendation:

- Specify that extracted data are derived from published studies- Consider providing a data extraction table as supplementary material

Minor Comments

5. Limitations Section

Present but underdeveloped

Should include:

- Search limitations- Publication bias- Lack of quality appraisal

Strengths of the Paper

- Addresses a critically under-researched topic- Uses a multidimensional systems perspective- Strong policy relevance- Inclusion of recent literature (2021–2025)- Good integration of social and health systems perspectives

**Confidential Comments to the Editor**

The manuscript has strong conceptual value but currently lacks the methodological rigor expected of scoping reviews. With substantial revisions; it could be suitable for publication.

No obvious competing interests or ethical concerns were identified.
