# Supplementary material for: Dementia care gaps in Sub-Saharan Africa: A scoping review of technology, environmental, community perspectives, and financial constraints
Source: PLOS Glob Public Health. 2026 Oct 6;6(10):e0007265. doi: 10.1371/journal.pgph.0007265 (PMC13641696; doi:10.1371/journal.pgph.0007265)
Supplement: Supplementary file 8 [file pgph.0007265.s008.docx]

**PLOS Global Public Health**

**Dementia Care Gaps in Sub-Saharan Africa: A Scoping Review of Technology, Environmental, Community Perspectives, and Financial Constraints**

--Manuscript Draft--

| **Manuscript Number:** | PGPH-D-26-00901R1 |
| --- | --- |
| **Article Type:** | Research Article |
| **Full Title:** | Dementia Care Gaps in Sub-Saharan Africa: A Scoping Review of Technology, Environmental, Community Perspectives, and Financial Constraints |
| **Short Title:** | SSA Dementia Review |
| **Corresponding Author:** | Maiya G. Block Ngaybe  The University of Arizona College of Architecture Planning and Landscape Architecture Tucson, AZ UNITED STATES OF AMERICA |
| **Order of Authors:** | Soarov Chakra Borty |
|  | Maiya G. Block Ngaybe, PhD, MPH |
|  | Angela E Apeagyei, PhD |
|  | Divya Bhagianadh, PhD, MPH |
|  | Monica H Swahn, PhD |
|  | Nazmus Sakib, PhD, MS |
| **Keywords:** | Dementia; Aging; Global Health; sub-Saharan Africa; health systems |
| **Abstract:** | Dementia is a growing public health challenge in Sub-Saharan Africa (SSA), where population aging is expected to substantially increase the regional burden of dementia in the coming decades. Rapidly changing demographics, underdeveloped health infrastructure, and limited awareness of dementia symptoms and care pathways contribute to delayed diagnosis, treatment, and care. This scoping review examined literature published from 2021 to 2025 to identify gaps in dementia care across four key  domains: environmental and social factors, technological capacity, community-based caregiving, and financial and economic factors. First, social and ecological factors, including low health literacy, rural residence, and weak primary care infrastructure, influence disease diagnosis and access to care. Second, technological capacity remains  limited, including scarce access to neuroimaging and diagnostic tools, limited telemedicine coverage, weak electronic health information systems, and insufficient digital health solutions for early detection, management, and monitoring. Third, evidence from community-based care highlights heavy reliance on informal caregivers who often lack training and institutional support, resulting in substantial emotional, physical, and financial burden. Finally, inadequate public financing for dementia care, coupled with limited insurance coverage, further restricts access to dementia care services and support. Overall, this review highlights critical gaps in policy, evidence generation, cost-effective technology investment, caregiver support, and health systems  strengthening across all levels of care. Addressing these multidimensional challenges is  crucial to improving the quality of life for people living with dementia and their caregivers across SSA. |
| **Additional Information:** |  |
| **Question** | **Response** |
| **Financial Disclosure** | The authors received no specific funding for this work. |

| Enter a financial disclosure statement that describes the sources of funding for the work included in this submission and the role the funder(s) played. This includes grants and any commercial funding of the work or authors.  This statement will be typeset if the manuscript is accepted for publication.  *Please review the* [*submission guidelines*](http://journals.plos.org/globalpublichealth/s/submission-guidelines#loc-financial-disclosure-statement) *and the instructions link below for detailed requirements and guidance.* |  |
| --- | --- |
| **Competing Interests**  On behalf of all authors, disclose any competing interests that could be perceived to bias this work.  This statement will be typeset if the manuscript is accepted for publication.  *Please review the instructions link below and PLOS Global Public Health’s* [*competing interests*](https://journals.plos.org/globalpublichealth/s/competing-interests) *policy to determine what information must be disclosed at submission.* | The authors have declared that no competing interests exist. |
| **Data Availability**  Before publication, Authors are required to make fully available and without restriction all data underlying their findings. Sharing the data underlying your findings increases the visibility, credibility, and impact of your work, while supporting transparency and future scientific discovery. Please see our [PLOS Data](https://journals.plos.org/globalpublichealth/s/data-availability) [Policy page](https://journals.plos.org/globalpublichealth/s/data-availability) for detailed information on this policy.  A **Data Availability Statement**, detailing where the data can be accessed, is required at first submission. Insert your | All data are publicly available as this is a review of peer-reviewed publications. |

| Data Availability Statement in the box below.  Please see the [data reporting](https://journals.plos.org/plosone/s/submission-guidelines#loc-data-reporting) section of our submission guidelines for instructions on what you need to include in your Data Availability Statement. Examples of acceptable Data Availability Statements are included here for reference:   1. The minimal data set [and accompanying code, where generated] is available at [repository name] via [DOI hyperlink] 2. No data was generated by this study. The following existing data sources were used: [dataset name] from [dataset location] available via [web address or DOI hyperlink]. 3. For Study Protocols: No datasets were generated or analysed during the current study. All relevant data from this study will be made available upon study completion.   *PLOS allows rare exemptions to address legal and ethical concerns. If you have legal or ethical restrictions, please use the box below to detail these in full sentences for the Journal team to consider.*  This statement will be typeset if the manuscript is accepted for publication. |  |
| --- | --- |

Manuscript	[Click here to access/download;Manuscript;main manuscript.pdf](https://www2.cloud.editorialmanager.com/pgph/download.aspx?id=836927&guid=7c6b1681-0184-4f17-8fe2-a88e3b4b5a5c&scheme=1)[<https://www2.cloud.editorialmanager.com/pgph/download.aspx?id=836927&guid=7c6b1681-0184-4f17-8fe2-a88e3b4b5a5c&scheme=1>](https://www2.cloud.editorialmanager.com/pgph/download.aspx?id=836927&guid=7c6b1681-0184-4f17-8fe2-a88e3b4b5a5c&scheme=1)

## Dementia Care Gaps in Sub-Saharan Africa: A Scoping Review of Technology, Environmental, Community Perspectives, and Financial Constraints

Soarov Chakra Borty^1^, Maiya G. Block Ngaybe^2^, Angela E Apeagyei^3^, Divya Bhagianadh^4^, Monica H Swahn^5^, Nazmus Sakib^1^

1 Department of Information Technology, Kennesaw State University, Kennesaw, Georgia, United States
2 School of Landscape Architecture, College of Architectural Planning and Landscape Architecture, University of Arizona, Tucson, Arizona, United States
3 Department of Health Metrics Sciences, School of Medicine, University of Washington, Seattle, Washington, United States
4 Department of Health, Human Performance, & Recreation, University of Arkansas, Fayetteville, Arkansas, United States
5 School of Public Health, Virginia Commonwealth University, Richmond, Virginia, United States

*[mgblock@arizona.edu](mailto:mgblock@arizona.edu)

## Abstract

Dementia is a growing public health challenge in Sub-Saharan Africa (SSA), where population aging is expected to substantially increase the regional burden of dementia in the coming decades. Rapidly changing demographics, underdeveloped health infrastructure, and limited awareness of dementia symptoms and care pathways contribute to delayed diagnosis, treatment, and care. This scoping review examined literature published from 2021 to 2025 to identify gaps in dementia care across four key domains: environmental and social factors, technological capacity, community-based caregiving, and financial and economic factors. First, social and ecological factors, including low health literacy, rural residence, and weak primary care infrastructure, influence disease diagnosis and access to care. Second, technological capacity remains limited, including scarce access to neuroimaging and diagnostic tools, limited telemedicine coverage, weak electronic health information systems, and insufficient digital health solutions for early detection, management, and monitoring. Third, evidence from community-based care highlights heavy reliance on informal caregivers who often lack training and institutional support, resulting in substantial emotional, physical, and financial burden. Finally, inadequate public financing for dementia care, coupled with limited insurance coverage, further restricts access to dementia care services and support. Overall, this review highlights critical gaps in policy, evidence generation, cost-effective technology investment, caregiver support, and health systems strengthening across all levels of care. Addressing these multidimensional challenges is crucial to improving the quality of life for people living with dementia and their caregivers across SSA.

## 1 Introduction

Dementia is an emerging public health concern in SSA due to epidemiological and demographic transitions, as well as significant inequities in access to diagnostic services

and long-term care services [1–6]. People living with dementia (PLWD) is projected to increase from 2.13 million in 2015 to 3.48 million by 2030 and 7.62 million by 2050 in SSA, although they currently account for a smaller share of global dementia cases than in high-income countries [7–9]. This relatively low reported case count is likely due to a high number of missed diagnoses, limited access to neuroimaging and cognitive assessment tools, shortages of trained dementia specialists, and weak referral pathways. Dementia in SSA remains broadly overlooked across international research, surveillance efforts, and policy agendas, despite these trends [10, 11]. Caregiving for PLWD is almost entirely the responsibility of informal caregivers (primarily women) in these settings, as in other low and middle-income countries (LMIC). Resource constraints and minimal healthcare infrastructure exacerbate the burden of dementia and limit access to formal care services. While health expenditure remains generally low at the national level across all LMICs, SSA bears the lowest health spending of any region in the world. In 2021, countries in SSA spent an estimated USD 92 per capita on health [12], compared with USD 379 per capita in North Africa and the Middle East, which ranked next lowest at the time. This disparity partly explains the strong reliance on informal caregivers seen in LMIC settings. Unpaid informal caregiving (65% of total costs) is the most significant contributor to the overall financial burden of dementia in LMICs, and most countries fail to offer any support to informal carers [11].

Since the 1990s, many SSA countries have experienced increasing life expectancy, with a focus on maternal and child health, infectious disease control, and primary health care [13]. As a result, the phenomenon of population aging has appeared more quickly than in previous generations, which has brought attention to the problem of dementia and other age-related health issues. In this regard, the problem of dementia and research on aging can be considered an increasingly important public health issue, which has brought a timely opportunity to develop and expand models of dementia care and research tailored to the aging population of SSA. Dementia in sub-Saharan Africa has historically received limited attention within health systems and policy agendas, though recent national and international efforts point to increasing engagement with dementia-related challenges. Recent studies from Ethiopia, Ghana, Kenya, Nigeria, South Africa, and Tanzania have examined dementia-related care gaps in SSA [14–18]. Together, these studies show that dementia care in SSA is shaped by sociocultural beliefs, limited awareness, reliance on family caregiving, broader health-system constraints, and gaps in diagnostic and financial support. Socioeconomic deprivation is also relevant to dementia in SSA, as evidence from South Africa shows an association between multidimensional poverty and dementia among adults aged 50 years or

older [19]. Families often face numerous challenges, including out-of-pocket costs, time spent on healthcare visits, transportation costs, assistive services availability, loss of leisure time, and psychosocial strain among caregivers [17, 20]. Coupled with limited health insurance coverage and weak social welfare systems, these pressures often lead to catastrophic health expenditures [18, 21], where households spend more than 10% of their household income on health care. Despite their importance, comprehensive and systematically quantified assessments of these technological, financial, and economic burdens remain limited in SSA [19–21].

Dementia care in SSA is characterized by intersecting challenges in research capacity, workforce development, and policy environments. This review examines the existing evidence on technological gaps, financial and economic barriers, and structural, clinical, and policy-related barriers to early diagnosis in SSA. Comprehensive dementia care encompasses numerous layers of the healthcare system, including technology, workforce capacity, community support, and financial protection. The four thematic domains used in this review are environmental and social challenges, technology-related challenges, community-based dementia care and informal caregiving challenges, and financial and

economic challenges, allowing gaps to be examined across prevention, diagnosis, care delivery, and long-term support. Together, these classifications are utilized as a systems-based organizing structure to frame the gap analysis, consistent with the WHO health system framework [22]. This systems-based structure supports cross-domain integration. This review focuses on studies/papers published between 2021 and 2025; earlier landmark analyses and global reports are cited to provide epidemiological context and conceptual framing.

In line with the World Health Organization (WHO) health system building blocks framework that views healthcare systems through the lens of six interrelated components: service delivery, health workforce, health information systems, medical products and technologies, financing, and leadership and governance, this review takes on the systems-based approach to provide insights on gaps in dementia care provision in SSA [22, 23]. This paper adds to the literature by providing a comprehensive review across multiple domains relevant to dementia care in SSA, with a focus on more recent studies. This review synthesizes the existing literature to identify research gaps and explore reported health care technologies to address issues across various aspects of the health system, including delayed diagnosis, inadequate care coordination, and

family-based caregiving.

## 2 Methods

### 2.1 Study Design and Reporting Framework

This study was conducted as a scoping review to map recent evidence on dementia care gaps for people living with dementia (PLWD) in SSA. A scoping review was appropriate because the evidence includes diverse clinical, health-system, policy, caregiving, and technology-related studies. The review followed established scoping review methodology and was reported according to the Preferred Reporting Items for Systematic Reviews and Meta-Analyses extension for Scoping Reviews (PRISMA-ScR) [24].

### 2.2 Information Sources and Search Strategy

A literature search was conducted in PubMed, Scopus, ScienceDirect, IEEE Xplore, and Google Scholar to identify studies on dementia care gaps in SSA. These databases were selected to capture multidisciplinary evidence from biomedical, public health, social science, health systems, and technology-related research. The search was limited to studies published between January 2021 and December 2025. The main Boolean search string combined terms related to dementia, geography, and care gaps: (”dementia” OR ”Alzheimer’s disease” OR ”cognitive impairment”) AND (”Sub-Saharan Africa” OR ”SSA” OR ”Africa”) AND (”care” OR ”caregiving” OR ”health system” OR ”healthcare access” OR ”diagnosis” OR ”treatment” OR ”technology” OR ”digital health” OR ”cost” OR ”financing”). Search terms were adapted as needed to match each database’s search functionality.

Google Scholar was used as a supplementary source to identify relevant grey literature and articles not indexed in the main databases. In addition, website searches were conducted using relevant organizational and public health sources, including the World Health Organization, Alzheimer’s Disease International, and dementia or public health websites relevant to SSA. Citation tracking of key included articles was also performed to identify additional eligible studies.

### 2.3 Eligibility Criteria

The studies were selected based on predetermined inclusion and exclusion criteria.

#### Inclusion criteria:

i. Studies related to dementia, Alzheimer’s disease, and other cognitive impairments.
ii. Studies conducted in, or relevant to, countries within SSA.
iii. Studies relevant to dementia care, caregiving, healthcare access, workforce issues, policy, health-system factors, or technology-related interventions.
iv. Original research articles, review articles, and studies published within the period 2021–2025.

#### Exclusion criteria:

i. Studies not relevant to dementia or cognitive impairments.
ii. Studies not relevant to SSA.
iii. Studies not relevant to the scope of dementia care and healthcare system issues.

**Fig 1.** Flow diagram of literature search and screening for dementia care gaps in SSA.

iv. Editorials, commentaries, opinion articles, letters, and papers without substantive analytical or evidence-synthesis content were excluded; systematic reviews, scoping reviews, narrative reviews, and policy analyses were included if they directly addressed dementia care gaps in SSA.

### 2.4 Study Selection Process

All records retrieved from databases and other sources were imported into a reference management file, and duplicate records were removed prior to screening. Study selection was conducted in two stages. First, four reviewers independently screened the titles and abstracts of all retrieved records against the predefined eligibility criteria.

Second, the full texts of potentially eligible studies were independently assessed by the same four reviewers to determine final inclusion. Inter-reviewer agreement among the four reviewers was assessed using Fleiss’ kappa statistic. Any disagreement during title/abstract or full-text screening was resolved through discussion and consensus. If consensus could not be reached, a fifth reviewer was consulted to make the final decision. Figure 1 illustrates the PRISMA flow diagram, which outlines the study identification, screening, eligibility, and inclusion process for the recent literature on dementia care gaps in SSA.

### 2.5 Synthesis of Results

The extracted data were synthesized and categorized using descriptive and thematic analysis to establish the status of dementia care in SSA. In this case, the gaps identified in four major areas, which include health systems capacity gaps, caregiving support gaps, service delivery and access gaps, and technology and innovation gaps, are targeted. The data synthesized is categorized under thematic areas, which include health system capacity gaps such as financial, economic, environmental, and social constraints, caregiving gaps such as training and support for informal and community-based caregivers, service delivery and access gaps such as fragmented care processes and access to care, and technology and innovation gaps such as the use and availability of technology in dementia diagnosis, care, and patient monitoring. A detailed evidence extraction matrix of the included studies is provided in S1 File.

## 3 Environmental and social challenges

This section focuses on the environmental, social, and systemic drivers that shape dementia diagnosis and management across Sub-Saharan Africa. Table 1 shows that 5 of the 33 included studies examined environmental and social challenges, with evidence primarily drawn from Nigeria, Kenya, South Africa, and broader SSA contexts. Based upon a retrospective analysis from Northeastern Nigeria in which Wakawa et al. [25] examined over two decades of patient records from the region’s sole neuropsychiatric facility, 1,216 cases of dementia among adults aged 60 years and older were documented. Alzheimer’s disease (60.5%) and vascular dementia (24.5%) were the most prevalent subtypes, with hypertension (41.6%) as the leading comorbidity. There was a limited diagnostic capacity due to a lack of neuroimaging tools, biomarkers, and a specialized workforce. This reflects the broader environmental and infrastructural constraints of dementia care in resource-limited regions. The authors emphasize that both vascular comorbidities and environmental stressors, such as chronic hypertension and

conflict-related trauma, may contribute to the region’s escalating dementia rates, underscoring the need for policy reforms that strengthen neurological health infrastructure and surveillance systems.

**Table 1.** Summary of included studies by thematic domain

| **Thematic domain** | **Number of studies** | **Main countries or regions represented** |
| --- | --- | --- |
| Environmental and social challenges | 5 | Nigeria, Kenya, South Africa, and broader SSA evidence |
| Technology-related chal­lenges | 12 | Nigeria, Kenya, Tanzania, Ghana, South Africa, East Africa, and broader SSA evidence |
| Community-based dementia care and informal caregiving challenges | 15 | Kenya, Uganda, Ghana, Benin, Nigeria, South Africa, Tanzania, the Demo­cratic Republic of the Congo, and broader SSA evidence |
| Financial and economic challenges | 5 | SSA-level, Africa-level, and global/regional modeling evidence including SSA |

Note: Studies could contribute to more than one thematic domain; therefore, the total number across domains exceeds 33.

Similarly, Wisdom et al. [26] identified environmental and occupational exposures as important but often overlooked contributors to neurodegenerative disease in Nigeria, including fine particulate air pollution, pesticide exposure, and groundwater contamination with heavy metals. Their analysis estimated that dementia prevalence has risen by nearly 400% over two decades, disproportionately affecting aging and

low-income populations. The authors argue for integrating dementia prevention into environmental health policies, noting that Nigeria’s current health governance lacks coherent frameworks linking neurodegenerative disease to ecological regulation or

non-communicable disease (NCD) control. Moreover, the concentration of neurologists and diagnostic centers in urban areas amplifies rural inequities and delays in diagnosis.

In South Africa, Ssonko et al. [27] conducted the first longitudinal analysis of memory clinic data from Groote Schuur Hospital in Cape Town, revealing that Alzheimer’s disease (70.9%) and vascular dementia (14.6%) accounted for the majority of 165 dementia cases. The study found that cognitive decline averaged 2.1 Mini-Mental State Examination (MMSE) points per year for Alzheimer’s and 1.3 for vascular dementia. Educational attainment and cardiovascular comorbidities, linked to environmental and lifestyle factors such as diet and urbanization, emerged as significant predictors of both cognitive decline and mortality. The study situates these findings within broader socioeconomic and infrastructural determinants, arguing that the limited formalized dementia registries and geriatric services in SSA hinder effective governance and continuity of care.

Beyond environmental stressors, social and cultural determinants deeply influence dementia risk, diagnosis, and management across SSA. Bosire et al. [28] conducted ethnographic research at the Aga Khan University Hospital in Nairobi, documenting healthcare providers’ perspectives on dementia diagnosis and management in Kenya. Although tertiary private hospitals offered multidisciplinary, globally aligned diagnostic pathways, providers highlighted the severe inequities between the private and public sectors. Most Kenyans lack access to neuroimaging, cognitive assessments, and specialized care from trained professionals. Stigma, misconceptions linking dementia to witchcraft or punishment, and the absence of a national dementia policy were recurrent themes. These sociocultural barriers delay care-seeking, limit early detection, and increase the burden on caregivers. The authors advocate for Kenya’s first national dementia strategy to bridge clinical, cultural, and governance divides.

In Nigeria, Wisdom et al. [26] further illuminated how gender, education, and social position intersect with stigma to shape the lived experience of dementia. Older women,

**Fig 2. Environmental and social risk factors affecting dementia diagnosis in SSA.** Dementia diagnosis in SSA is shaped by interacting environmental, social, and structural constraints, including limited access to neuroimaging and trained specialists, as well as pronounced rural–urban inequities in diagnostic services.^1,2^ Sociocultural misconceptions, stigma, and the absence of formal dementia policy frameworks further delay care-seeking and hinder early detection.^3^ **Notes:** ^1^ Diagnostic capacity and specialist workforce limitations in resource-constrained settings [25, 27]. ^2^ Environmental and occupational exposures linked to neurodegenerative risk [26]. ^3^ Sociocultural barriers, stigma, and policy absence affecting dementia recognition and diagnosis [28].

in particular, face discrimination and violence due to widespread beliefs that cognitive decline results from witchcraft. Informal caregivers, predominantly women, bear over 70% of unpaid care hours, often without institutional or financial support. These findings highlight important governance and policy implications, suggesting that dementia should be framed not only as a medical condition but also as a social issue with relevance to aging and gender equity policies.

While pharmacological interventions remain limited, psychosocial and behavioral strategies have shown promise for addressing this issue. Beyene et al. [29] conducted the first systematic review of dementia management practices in SSA, analyzing randomized clinical trials and cohort studies across eight countries. They found that Cognitive Stimulation Therapy (CST), a low-cost, group-based behavioral intervention, was feasible and effective in improving cognitive and language outcomes in low-resource settings. Furthermore, early initiation of combination antiretroviral therapy was associated with improved cognitive outcomes among individuals with HIV-associated dementia (HIVAD). The review also revealed systemic gaps, including a lack of culturally validated diagnostic tools, inadequate training of community health workers, and limited support networks for caregivers. The authors call for scaling up CST and integrating dementia management into primary healthcare systems through

task-shifting and training initiatives. These findings align with Wakawa et al. [25], who advocate for decentralized care models to extend dementia services beyond tertiary facilities. The establishment of the Northern Nigeria Dementia Research Group marks a significant step toward creating a national dementia registry and promoting the translation of research into policy and practice. Both studies emphasize the importance of governance structures that facilitate early detection, community-based care, and the sharing of regional data. Figure 2 summarizes the environmental, cultural, and social factors contributing to disparities in dementia care across SSA.

Collectively, these studies demonstrate that dementia in SSA is shaped by

intersecting environmental, social, and systemic factors that demand an integrated governance response. Three broad policy imperatives emerge. First, it is essential to integrate dementia into the National Health Frameworks and plans. Dementia should be formally embedded within national aging, NCD, and environmental health policies.

Governments can align with the WHO Global Action Plan on the Public Health Response to Dementia (2017–2025) by developing country-specific dementia strategies, establishing registries, and promoting caregiver protections. Secondly, capacity building and decentralization of efforts are key. Investment in workforce training, particularly in task-shifting, primary care, and community health workers, is essential. There is also evidence that this approach, supported by findings from Ethiopia, Nigeria, and Kenya, can bridge diagnostic and treatment gaps in rural regions. Finally, cross-sectoral and international collaboration is essential. Effective governance needs to extend beyond the health sector to address the broader social and environmental determinants of dementia. Integration with ecological regulation (air quality, pesticide control), education, and social welfare systems is key. Furthermore, partnerships between African governments, research consortia, and global funding bodies can support evidence-based policymaking and the development of research infrastructure.

The growing body of dementia research in SSA provides critical insights into how environmental exposures and social inequities converge to shape cognitive aging. Across Nigeria, Kenya, South Africa, and Ethiopia, recent evidence suggests the need for urgent governance reforms that integrate environmental stewardship, social protection, and health system strengthening. By reframing dementia as both a public health and a social governance challenge, SSA can lead the development of a new, integrated, and equity-centered global health policy model for the prevention and care of neurodegenerative diseases.

## 4 Technology-related challenges

Digital health technologies can enhance dementia care in low-resource settings; however, their adoption across SSA remains minimal due to infrastructural, financial, and training limitations. As shown in Table 1, technology-related challenges were reported in 12 of the 33 included studies and were represented across Nigeria, Kenya, Tanzania, Ghana, South Africa, East Africa, and wider SSA evidence. Internet connectivity is inconsistent and often unavailable in rural areas, and many healthcare facilities lack a reliable power supply and the necessary hardware to conduct telemedicine and remote consultations [30, 31]. Telemedicine, tele-support for caregivers, has been investigated as one of the solutions for extending care for dementia patients, but the development of this type of service in SSA is uneven due to problems with information technology infrastructure. The systematic reviews of the effects of telemedicine in low- and middle-income countries, including SSA, suggest the potential of telemedicine to improve access to specialist consultation and caregiver support, while also identifying persistent barriers related to infrastructure reliability, workforce training, and integration with existing health systems. One key area that could be improved by applying technological advancements to SSA’s support structure for dementia is standardization and greater accessibility of screening. With so many different cultural settings and linguistic patterns across SSA, a lack of standardized screening methods could merely reflect a scarcity of understanding and preparation within healthcare.

Frontline health workers in many countries misclassify dementia symptoms with “normal aging,” “stress,” or general cognitive decline as a person gets older [32, 33].

Importantly, efforts to improve dementia screening and diagnosis in SSA should not assume that dementia screening tools and care pathways can be directly transferred without local adaptation. Local beliefs about aging, memory loss, family responsibility,

and what constitutes normal versus pathological cognitive decline need to be understood and incorporated into dementia policies and interventions. Culturally grounded approaches, developed with communities, caregivers, and frontline health workers, may help reduce stigma, improve acceptability, and ensure that screening tools and care pathways are locally appropriate [30, 32, 33]. Training challenges, cultural adaptation, and health system preparedness are some of the reasons for the unequal administration of cognitive assessment tests like the Mini-Mental State Examination (MMSE) and the Montreal Cognitive Assessment (MoCA), especially in cases where test content is unavailable in local languages or culturally adapted forms across SSA [30].

Another technological barrier is the limited availability of laboratory infrastructure, neuroimaging, and biomarker assays for diagnosing dementia. Positron emission tomography scans, molecular imaging, and Cerebrospinal Fluid (CSF) biomarker testing are cutting-edge technologies available in high-income countries but are limited in

SSA [25]. Moreover, in SSA, access to MRI imaging, which is essential for dementia diagnosis, is severely constrained by restricted access, high costs, and frequent equipment downtime [26]. Due to limited access to diagnostic technologies, health care practitioners are often left to diagnose based on clinical history alone, making it difficult to distinguish dementia from other causes of intellectual impairment, such as tumors, stroke, and hydrocephalus. This diagnostic uncertainty increases the risk of dementia being underdiagnosed or misdiagnosed. For instance, in rural Kenya and Nigeria, cognitive symptoms can be misattributed to depression, epilepsy, or psychiatric conditions because of the lack of appropriate screening tools and specialized diagnostic equipment [26, 34]. Moreover, Yenesew et al. [35] describe how East African research organizations lack the capacity in biomarker, genetic, and epigenetic research, further limiting region-specific understanding of dementia etiology. This also limits the infrastructure, such as screening tools, culturally adapted interventions, and predictive models of dementia risk. Furthermore, limited coordination among dementia-related data systems may create a cyclical barrier, hindering investment in technology due to insufficient data. Without technology, data cannot be collected and studied properly. Without these basic technologies, SSA countries remain constrained in producing population-level data on dementia subtypes and risk factors. Combined with limited financial resources, structural barriers linked to laboratory infrastructure, biomedical engineering capacity, and maintenance support further restrict the use of modern diagnostic technologies for dementia diagnosis and care. These limit the use of modern technologies in diagnostic equipment for proper dementia care.

The technological gap is also deeply interconnected with human resource shortages. Across SSA, there is a scarcity of individuals with expertise in dementia care, including neurologists, geriatricians, psychiatrists, and neuropsychologists [32, 36, 37]. With a population of 200 million people, Nigeria has only 90 neurologists, substantially constraining the diagnostic capacity of neurological conditions, including dementia [26]. Due to a lack of specialists, mobile and digital screening tools often yield unsatisfactory results, as these tools require confirmatory diagnosis and clinical oversight [38]. Primary healthcare workers often report low confidence in cognitive assessments and in distinguishing dementia from other neurological or psychiatric disorders because of the limited dementia content in medical and nursing curricula. This contributes to diagnostic misclassification, delayed referrals, and the perpetuation of stigmatizing opinions about dementia [33]. The specialist shortage is therefore not simply a workforce issue; it is also a structural technological hindrance that restricts the performance of diagnostic tools, reduces the usefulness of digital screening innovations, and restricts the establishment of multidisciplinary dementia clinics [38]. Access to pharmacological remedies, including acetylcholinesterase inhibitors (donepezil, rivastigmine) and memantine, is minimal due to cost, stock-outs, or absence from

**Fig 3. Technology gaps and limitations affecting dementia care.** Dementia care in SSA is constrained by fragmented and non-standardized screening tools, limited diagnostic technologies, and weak digital health infrastructure, hindering early diagnosis and continuity of care.^1,2^ Structural barriers, including scarce memory clinics, weak referral pathways, and shortages of trained specialists, further limit the effectiveness of technology-enabled interventions.^3^ Persistent system-level constraints affecting telemedicine, mHealth adoption, and dementia data systems remain evident across the region.^4^ **Notes:** ^1^ Limited cultural adaptation of cognitive screening tools [30, 32]. ^2^ Restricted access to neuroimaging and biomarker-based diagnostics [25, 26]. ^3^ Shortages of specialists and memory clinic infrastructure [36, 37]. ^4^ Infrastructure barriers affecting telemedicine and dementia data systems [31, 39].

national formularies [26, 30].

Studies from countries including Ghana, South Africa, and Tanzania reveal similar trends: healthcare providers often lack essential tools to diagnose cognitive changes and functional decline, despite observing these changes [36–38]. Mobile health (mHealth) systems play a crucial role in caregiver education, symptom monitoring, and early disease detection. However, studies indicate that increased dropout rates, usability challenges, and poor integration with existing health care strategies are issues in

SSA [30, 39]. App-based screening tools deployed in Tanzania show potential, but there are some limitations, including the fact that digital screening cannot substitute confirmatory diagnosis, and referral pathways to specialist care remain fragile [38].

Moreover, Paddick et al. demonstrated the feasibility of using a tablet-based dementia screening app that incorporated culturally adapted cognitive assessment items and was administered by non-specialist community health workers. However, they highlighted the limited scope of technology-driven approaches in the absence of broader systemic changes to address issues such as limited specialist referral capacity and insufficient training. Further, without validation, scalable screening technology, and a robust health strategy, early detection will remain unstable. SSA countries cannot fully leverage the benefits of telemedicine or digital caregiver support without national digital health strategies, electronic health records, and stable digital networks. One of the most

pressing challenges is the absence of dedicated, interoperable dementia-specific data systems, despite the presence of broader digital health infrastructures such as electronic health records and DHIS2 platforms in countries including Nigeria, Kenya, and Ethiopia [31, 39]. Without this mechanism, it is challenging for policymakers to make a decision on the prevalence, geographic allocation, and socioeconomic effects of dementia. Figure 3 illustrates the key technology gaps and limitations that affect dementia diagnosis and care delivery in SSA.

## 5 Community-based dementia care and informal caregiving challenges

This subsection discusses community-based dementia care and informal caregiving, which aligns with the WHO’s health system building block of service delivery and its focus on people-centered, community-oriented care. Community-based dementia care and informal caregiving challenges formed the largest thematic group, appearing in 15 of the 33 included studies, with evidence from Kenya, Uganda, Ghana, Benin, Nigeria, South Africa, Tanzania, the Democratic Republic of the Congo, and broader SSA settings (Table 1). Informal care is typically provided in multigenerational households with limited resources and minimal support from the formal health system [40], and caregiving demands increase with increasing severity of behavioral and psychological symptoms of dementia (BPSD) [41]. Studies predominantly reported women as informal caregivers for PLWD, with proportions ranging from 70% to 90% across settings in Uganda, Benin, Tanzania, Ghana, and the Democratic Republic of Congo. Caregivers themselves are often older adults or middle-aged individuals, frequently spouses or adult daughters of care recipients [40, 42–45]. Studies also reveal limited knowledge about dementia, and high levels of unmet support needs among caregivers [40, 46, 47]. While explicit quantification was not available across these studies, the Benin time-use evaluation study estimated that caregivers spend 8-12 hours/day on informal caregiving activities like supervising the PLWD, assisting with activities of daily living (ADL), and managing behavioral symptoms [45].

Studies reported higher caregiving burden across the different country settings.

Three studies specifically reported caregiving burden using a standardized Zarit Burden Interview (ZBI) that assesses subjective caregiver burden and reported moderate to severe burden among informal caregivers of PLWD [42–44]. Studies also reported psychological distress among caregivers with reports of high prevalence of depression, anxiety, and emotional exhaustion. Okwaraji et al. [48] reported a strong association between BPSD severity and burnout and psychological stress among caregivers in Nigeria. They provide the most detailed quantification of caregiver burden, with 40% of caregivers experiencing psychological distress and nearly 25% experiencing emotional exhaustion. Functional impairment and higher BPSD severity were also associated with greater supervision needs and frequent nighttime caregiving requirements [41, 44].

Studies also showed a significant economic burden associated with dementia caregiving in the SSA setting. Caregiving can disrupt employment, as caregivers often struggle to balance their work and caregiving responsibilities. Caregivers often report reducing work hours, leaving jobs entirely, or shifting to informal labor to accommodate caregiving responsibilities [45, 48, 49]. Th´ebaut et al. [45] provided a direct economic quantification of dementia care costs in Benin, estimating the annual dementia care cost in 2021 to be purchasing power parity dollars (PPP$) 2,399. An estimated 92% of this cost was attributed to informal care. This high number underscores the substantial economic burden borne by families when caring for people with learning disabilities. A national situational analysis from South Africa linked dementia-related care

**Fig 4. Community-based dementia care and informal caregiving in SSA.** Evidence indicates that dementia care in SSA is predominantly home and community-based, with informal caregiving largely undertaken by women and characterized by substantial time demands and psychological burden.^1,2^ Limited caregiver training, delayed diagnosis, and weak health-system support further shift

responsibility to households, contributing to economic strain and unmet care needs.^3,4^ **Notes:** ^1^ Gendered informal caregiving roles and caregiver burden [40, 43, 44]. ^2^ Associations between behavioral and psychological symptoms of dementia and caregiver distress [41, 42, 48]. ^3^ Time-intensive caregiving and economic impacts of informal

care [45, 49]. ^4^ Health-system constraints, delayed diagnosis, and limited service availability [28, 49, 50].

expenditures (not just limited to informal caregiving-related expenses) to chronic financial instability [49].

Information on interventions and their effectiveness is limited by study design and lack of generalizability. A study in Uganda found positive associations between interventions such as gardening and improved mental health scores, as well as lower psychological distress among caregivers [51]. A singing training program in South Africa showed associations with improved dementia caregiver confidence [52]. A community intervention focused on stigma reduction demonstrated high rates of acceptance and feasibility, with marked improvements in caregivers’ attitudes towards dementia [53].

Health system resource constraints severely impact PLWD and their caregivers in SSA, both directly and indirectly. Sparse dementia specific services that remain fragmented, underdeveloped, or absent place a heavy burden on informal caregivers [49]. Underdiagnosis or delayed diagnosis of dementia due to a lack of resources creates a cycle of unmanaged symptoms and delayed care with implications for caregivers.

Workforce shortages and gaps, including low provider knowledge, may impact

care-seeking behavior [49, 50]. The responsibility of managing undiagnosed PLWD falls squarely on family members. Informal caregivers themselves receive little to no training on managing dementia symptoms. Unreliable access to medicines, coupled with a lack of information and stigma, may drive families to seek traditional healers, further delaying medical diagnosis and treatment. A lack of financial protection and dependence on out-of-pocket household spending lead families into catastrophic health spending. This financial instability is compounded by the loss of caregiver income [45, 49].

The evidence from SSA highlights substantial caregiver burden, limited or

non-existent caregiver support, and gendered caregiving norms, all of which are further compounded by weakened health system infrastructure. Figure 4 depicts informal caregiving dynamics and locally rooted support systems for dementia care in SSA.

## 6 Financial and economic challenges

Financial and economic barriers remain central determinants of access, quality, and sustainability of dementia care across SSA. Financial and economic challenges were addressed in 5 of the 33 included studies, drawing on SSA, Africa-wide, and global or regional modeling evidence, as summarized in Table 1. Three recent studies included estimates of dementia costs in SSA. Two of these studies were global dementia cost studies that reported estimates of dementia costs in SSA [54, 55]. The remaining

one [56] highlighted results from an earlier study, Guerchet et al. [8]. In Pedroza et al. [54], the methodology relied on a systematic review of the published literature to generate estimates of costs associated with dementia care. These studies mainly

captured costs related to spending on direct health services or monetary costs. Because there are very few cost studies based on primary data published for people living with dementia in SSA to inform modeling, their estimates relied on modeling costs based on covariates and assumptions. The results from the modelling study show that in 2019, for SSA, the spending attributable to dementia was $552 million (Uncertainty interval:

$325-$816 million), 0.7% of total health spending. This estimate was projected to rise to $6,955 million ($3,401 - $12,001 million) by 2050. The second study, Chen et al. [55], reported data on the economic costs (including both monetary and non-monetary costs) of dementia in SSA, based on a macroeconomic model simulation. Specifically, the

authors used a health-augmented macroeconomic model to estimate the macroeconomic burden of Alzheimer’s disease and other related dementias (ADODs). Their model accounted for labor supply effects for the person living with dementia (morbidity and mortality) as well as for their caregivers (reduction in hours of work), as well as age-sex differences, education, work experience, treatment, and formal care costs, diverting from investments and savings. The authors estimate that between 2020 and 2050, the economic burden of ADODs in SSA will be approximately $158 billion (126-199) in international dollars. This translates to 0.108 as a proportion of total economic output (gross domestic product) between 2020 and 2050. Given the rising proportion of the population above the age of 60, these costs could potentially increase rapidly if the appropriate policies and interventions are not implemented to manage them.

While there exists anecdotal and qualitative evidence that the indirect costs, such as informal caregiving, constitute substantial aspects of the economic burden of dementia care for families with people living with dementia, there is limited observed or documented data on these costs. The few existing estimates are modelled approximations from earlier years [56]. It is reported that in 2015, dementia cost SSA $

6.2 billion, of which more than two-thirds (70%) was attributable to informal care provided by the families of people living with dementia. Furthermore, the existing structure of care for people living with dementia in most SSA countries relies predominantly on families providing most of the non-medical support required by people living with dementia. The high proportion of carers signals a potentially enormous cost to national output that is unaccounted for. This is an unaccounted cost because the economies of several SSA countries have significant informal sectors; therefore, such support provided informally is likely under-reported in official records. As such, quantifying non-financial and informal costs will require innovative documentation and analysis.

The existing literature mostly reports modeling costs based on limited data. Hence,

the evidence base has yet to focus on the sources and mechanisms that people living with dementia in SSA use to pay for care. However, there is some evidence on how older adults generally pay for care in SSA. This broader literature base suggests a heavy reliance on household out-of-pocket spending [57]. Additionally, given the general disease burden in SSA countries, we observe that even in countries with national health insurance systems, the benefits plan tends to prioritize infectious diseases over

non-communicable diseases and may not always cover medications. Nonetheless, older populations may be exempt from paying enrollment or premium fees to access benefits under national health insurance programs. Typically, the burden of financing falls on the individual and their family. These include not only direct spending on consultation visits, medication procurement, and diagnostics, but also the bulk of the responsibility for assisting with activities of daily living when necessary, as part of the care plan. In most SSA countries, this responsibility falls predominantly on women within families.

The potential cost drivers in SSA include a rising burden of stroke and other cardiovascular diseases, an aging population, and a population with a relatively lower education level [30]. Understanding the drivers of these financial and economic costs may help shed light on the broader societal implications for the future and highlight potential pathways for addressing, managing, and responding appropriately. While the nascent stage of this research topic means that there are limited studies that primarily explore the determinants of financial and economic costs borne by people living with dementia, there are descriptive suggestions of potential cost drivers in the limited epidemiological studies that do exist. The cost drivers highlighted above suggest that a

**Fig 5. Financial and economic challenges shaping dementia care systems in SSA.** The literature indicates a reliance on modeled cost estimates due to limited primary cost data^1^, a predominant dependence on household out-of-pocket financing^2^, and largely informal, family-based caregiving responsibilities that disproportionately fall on women^3^. **Notes:** ^1^ Synthesized evidence from cost modeling studies in SSA. ^2^ [57]

^3^ [30]

response strategy focused on a life-course and prevention-oriented approach might be appropriate to keep both financial and non-financial costs within manageable limits.

Leveraging the World Health Organization’s six building blocks of a health system framework, the financing principles could identify potential pathways for countries in SSA to comprehensively cover the financial and economic costs of dementia care for people living with dementia and their families. The financing block proposes three functions of a financing system: mobilizing revenue, pooling resources, and purchasing services. Mobilizing revenue refers to the process of raising funds to cover the costs of health services. Pooling addresses the importance of sharing risks across groups by aggregating individuals and resources into groups, enabling them to benefit from economies of scale. Lastly, purchasing refers to the collective mobilization of resources to procure the necessary health goods and services. Countries in SSA vary in their macroeconomic contexts, fiscal space, and potential for revenue mobilization; as such, each country’s approach to developing a comprehensive financing plan for dementia will need to take local constraints into account. Nonetheless, there are global examples of approaches to revenue mobilization, pooling, and purchasing that countries can draw on to develop contextually appropriate responses. For SSA in particular, a key consideration is the relatively young population and the opportunities it presents to develop a financing strategy that prioritizes a prevention-oriented approach to harness gains from a preemptive stance. In terms of specific examples, South Korea and Brazil offer models for pursuing a preventive approach and leveraging a universal health care platform to provide comprehensive coverage for dementia treatment, as well as the formal caregiving support required in later stages through long-term care coverage.

Figure 5 illustrates key financial and economic barriers influencing the organization and sustainability of dementia care systems in SSA.

In summary, the financial and economic costs of dementia in SSA are an area that is underexplored and underrepresented in the current literature. More primary data on costs by severity, age, sex, and caregiver-related factors across the different countries in SSA are needed. While SSA is currently considered a relatively young population, its older population is set to increase substantially by 2050. Understanding the potential financial and economic ramifications fully will be essential for planning appropriately on the continent.

## 7 Discussion

This review highlights the challenges in dementia care in SSA and addresses both social and economic issues. The literature reviewed identifies technology gaps as a significant barrier to early diagnosis, effective monitoring, and coordinated care. Many countries continue to face limited cognitive screening capacity, digital health infrastructure, and trained personnel to deploy telemedicine-based support tools. At the same time, several emerging technologies, including mobile screening applications and community-based digital tools, show promise for improving dementia care in SSA. For example, expanding mobile phone use, growing digital health initiatives, and the increasing prioritization of non-communicable diseases in global health agendas provide a platform for developing and testing cost-effective, sustainable, and innovative approaches to dementia care.

However, challenges related to scalability, limited internet access in rural areas, and insufficient frontline provider training complicate implementation.

Misunderstandings about culture and lack of dementia literacy stop people from getting help and adhering to care pathways, and environment and social factors contribute to inequality. Rural inhabitants have greater difficulty accessing specialized care facilities due to limited service accessibility and insufficient development of the primary care network. Social aspects such as poverty, gender differences, and inadequate

education of the caregiver affect caregiver burden and patient outcomes. This review points out the importance of informal caregivers, mostly women, acting as the primary caregivers without any formal training, respite care, or psychosocial assistance.

In many SSA countries, patients lack insurance coverage and have limited access to government-funded services, including formal care, diagnostic services, and medications. Very few countries have national dementia policies, and these findings emphasize the urgent need for integrated, context-specific solutions that address structural, technological, and social barriers simultaneously. In addition to funding, the results also indicate a set of interrelated weaknesses in the other building blocks of health systems, as defined by WHO, such as service delivery, health workforce capacity, health information systems, and governance, which collectively contribute to the fragmentation and scalability challenges of dementia care in SSA.

A significant strength of this review is its synthesis of the recent literature (2021-2025), which provides an overview of the challenges and technological

opportunities in the care of individuals living with dementia in SSA. Additionally, the thematic synthesis helps capture the various aspects of the care system, caregivers, and technology that have otherwise been treated in isolation. However, the review has several limitations. First, the literature review is based on documented literature, which may not capture the undocumented community practices. Additionally, the heterogeneity of studies across countries in SSA makes direct comparisons difficult.

Finally, the review is based on a narrative synthesis, which did not allow for calculating intervention efficacy. Nonetheless, the findings of the review help guide the development of the literature, policies, and technology in the care of individuals living with dementia in SSA.

## 8 Conclusion

Nevertheless, the current policy interest in aging populations, e-health, and multisector cooperation offers valuable opportunities to improve dementia care and research in SSA. In particular, this scoping review brings together the latest scientific evidence on dementia care across four interconnected areas: environmental and social determinants, technological capabilities, community-based caregiving, and economic barriers. The analysis shows that the issue of dementia care in SSA is shaped by a combination of technological, social, community, and economic inequalities.

Based on the WHO health system framework, this review underscores the role of deficits in health financing, service delivery, human resources, information, and governance in the lack of robust dementia care systems in SSA. Overcoming these deficits is possible through the development of national dementia policy, integration of dementia services in primary healthcare facilities, caregiver training programs, public education, community-based dementia care, affordable digital health innovations, and increased financial protection for people with dementia and their caregivers.

Future studies should focus on using affordable, culturally appropriate, and scalable models of dementia care delivery. For instance, building region-specific dementia data warehouses would help build evidence by collecting harmonized data on dementia prevalence, diagnosis, service delivery, care providers’ needs, cost of care, technology use, and patient outcomes within SSA countries. A dementia data warehouse would assist policymakers in identifying underserved areas, assessing inequalities, evaluating interventions, and allocating resources using locally collected evidence. The following would be necessary for improving dementia care services within the SSA region.

## Supporting information

**S1 File. Evidence extraction matrix of included studies on dementia care gaps in Sub-Saharan Africa.**

## References

1. Prynn JE, Davey C, Davis D, Kuper H, Mugisha J, Seeley J. Cognition in older adults in Uganda: Correlates, trends over time and association with mortality in prospective population study. PLOS global public health. 2023;3(11):e0001798.
2. Brooke J, Ojo O. Contemporary views on dementia as witchcraft in sub-Saharan Africa: A systematic literature review. Journal of clinical nursing.

2020;29(1-2):20-30.

3. Adebisi AT, Salawu MA. Misconception of dementia-related disorders in Sub-Saharan Africa. Frontiers in Neurology. 2023;14:1148076.
4. Mavrodaris A, Powell J, Thorogood M. Prevalences of dementia and cognitive impairment among older people in sub-Saharan Africa: a systematic review. Bulletin of the World Health Organization. 2013;91:773-83.
5. Olayinka OO, Mbuyi NN. Epidemiology of dementia among the elderly in sub-Saharan Africa. International Journal of Alzheimer’s disease. 2014;2014(1):195750.
6. Wakawa IA, Musami UB, Kwairanga SH, Ogualili PN, Mahmood MY, Fugu MA, et al. Management of Dementia in a Resource-Constrained Sub-Saharan African Setting: Outcome of a Retrospective Survey of Clinical Practice in the Only Neuropsychiatric Facility in Northeastern Nigeria. medRxiv. 2024:2024-09.
7. Prince M, Wimo A, Guerchet M, Ali GC, Wu YT, Prina M. World Alzheimer report 2015. The global impact of dementia: an analysis of prevalence, incidence, cost and trends.; 2015.
8. Guerchet M, Mayston R, Lloyd-Sherlock P, Prince M, Akinyemi R, Paddick SM, et al. Dementia in sub-Saharan Africa: Challenges and opportunities. London, UK: Alzheimer’s Disease International; 2017.
9. Nichols E, Steinmetz JD, Vollset SE, Fukutaki K, Chalek J, Abd-Allah F, et al. Estimation of the global prevalence of dementia in 2019 and forecasted prevalence in 2050: an analysis for the Global Burden of Disease Study 2019. The Lancet Public Health. 2022;7(2):e105-25.
10. Akinyemi RO, Owolabi MO, Ihara M, Damasceno A, Ogunniyi A, Dotchin C, et al. Stroke, cerebrovascular diseases and vascular cognitive impairment in Africa. Brain research bulletin. 2019;145:97-108.
11. Organization WH, et al. Global status report on the public health response to dementia. Geneva, Switzerland: World Health Organization; 2021. Available from: <https://www.who.int/publications/i/item/9789240033245>.
12. Apeagyei AE, Lidral-Porter B, Patel N, Solorio J, Tsakalos G, Wang Y, et al. Financing health in sub-Saharan Africa 1990–2050: Donor dependence and expected domestic health spending. PLOS Global Public Health. 2024;4(8):e0003433.
13. Violante F, Collaborators GD, et al. Global age-sex-specific fertility, mortality, healthy life expectancy (HALE), and population estimates in 204 countries and territories, 1950-2019: a comprehensive demographic analysis for the Global Burden of Disease Study 2019. The Lancet. 2020;396(10258):1160-203.
14. Yoseph M, Paddick SM, Gray WK, Andrea D, Barber R, Colgan A, et al. Prevalence estimates of dementia in older adults in rural Kilimanjaro 2009–2010 and 2018–2019: is there evidence of changing prevalence? International Journal of Geriatric Psychiatry. 2021;36(6):950-9.
15. Mushi D, Rongai A, Paddick SM, Dotchin C, Mtuya C, Walker R. Social representation and practices related to dementia in Hai District of Tanzania. BMC Public Health. 2014;14(1):260.
16. Ogunniyi A, Hall K, Baiyewu O, Gureje O, Unverzagt F. Caring for individuals with dementia: the Nigerian experience. West African journal of medicine. 2005;24(3):259-62.
17. Aboderin IA, Beard JR. Older people’s health in sub-Saharan Africa. The Lancet. 2015;385(9968):e9-e11.
18. Amoakoh-Coleman M. Tackling Ghana’s dementia underdiagnosis needs investment in workforce, diagnostics, education and insurance. Nature Aging. 2025:1-1.
19. Trani JF, Moodley J, Maw MTT, Babulal GM. Association of multidimensional poverty with dementia in adults aged 50 years or older in South Africa. JAMA Network Open. 2022;5(3):e224160.
20. Wimo A, Guerchet M, Ali GC, Wu YT, Prina AM, Winblad B, et al. The worldwide costs of dementia 2015 and comparisons with 2010. Alzheimer’s & Dementia. 2017;13(1):1-7.
21. Eze P, Lawani LO, Agu UJ, Acharya Y. Catastrophic health expenditure in sub-Saharan Africa: systematic review and meta-analysis. Bulletin of the World Health Organization. 2022;100(5):337.
22. Indicators AHO. Monitoring the building blocks of health systems. Geneva, Switzerland: WHO Document Production Services. 2010.
23. Doshmangir L, Sanadghol A, Kakemam E, Majdzadeh R. The involvement of non-governmental organisations in achieving health system goals based on the WHO six building blocks: A scoping review on global evidence. PLoS One. 2025;20(1):e0315592.
24. Tricco AC, Lillie E, Zarin W, O’Brien KK, Colquhoun H, Levac D, et al. PRISMA extension for scoping reviews (PRISMA-ScR): checklist and explanation. Annals of internal medicine. 2018;169(7):467-73.
25. Wakawa IA, Musami UB, Kwairanga SH, Ogualili PN, Mahmood MY, Fugu MA, et al. Dementia in a resource-constrained sub-Saharan African setting: A comprehensive retrospective analysis of prevalence, risk factors, and management at the only neuropsychiatric facility in Northeastern Nigeria. Alzheimer’s & Dementia. 2025;21(3):e14538.
26. Kelechi Wisdom E, Soyemi T, Mayowa S, Ede NS, Ubalaeze Solomon E, Iloanusi CA, et al. Building healthcare capacity for neurodegenerative disease management in Nigeria: Challenges and opportunities. Journal of Public Health Research. 2025;14(2):22799036251350957.
27. Ssonko M, Hardy A, Naganathan V, Kalula S, Combrinck M. Dementia subtypes, cognitive decline and survival among older adults attending a memory clinic in Cape Town, South Africa: a retrospective study. BMC geriatrics. 2023;23(1):829.
28. Bosire EN, Blackmon K, Kamau LW, Udeh-Momoh C, Sokhi D, Shah J, et al. Healthcare providers perspectives and perceptions of dementia diagnosis and management at the Aga Khan University Hospital, Nairobi, Kenya. Journal of Alzheimer’s Disease. 2025;104(3):862-74.
29. Beyene DA, Berha AB. Management Practice and Clinical Outcomes of Dementia in Sub-Saharan Africa: A Systematic Review. Behavioural Neurology. 2023;2023(1):2307443.
30. Alemayehu ZG, Ayalew BD, Sime BL, Bonger TD, Zewdie YA, Keraga AS, et al. Dementia in Sub-Saharan Africa: Risk Factors, Public Perception, and Management Approaches. Journal of Medicine, Surgery, and Public Health. 2025:100204.
31. Oyinlola O. Addressing the challenges of dementia care in Nigeria: a call for a comprehensive national strategy. Public Health Challenges. 2024;3(2):e186.
32. Musyimi CW, Muyela LA, Mutiso VN, Mutunga E, Ndetei DM. Understanding dementia care pathways for policy development and service planning in Kenya. Dementia. 2023;22(5):1027-37.
33. Mwendwa P, Mutunga E, Kroll T, De Bru´n A. ‘It is stressful, almost every hour. . . ’: Experiences of caring for people living with dementia in Kenya—An interpretive phenomenological approach. Dementia. 2021;20(8):2916-32.
34. Aballa A, Khakali L, Njoroge W, Kamau LW, Merali Z, Bosire EN. “They are perceived to be witches, and some are killed”: Community perceptions of elderly people living with dementia in Kilifi, Kenya. Journal of Alzheimer’s Disease. 2025;103(2):593-604.
35. Yenesew MA, Krell-Roesch J, Fekadu B, Nigatu D, Endalamaw A, Mekonnen A, et al. Prevalence of Dementia and Cognitive Impairment in East Africa Region: A Scoping Review of Population-Based Studies and Call for Further Research. Journal of Alzheimer’s Disease. 2024;100(4):1121-31.
36. Aryee CL. A Systematic Review of Creating an Ideal Adult Day Center for People with Dementia in Ghana (Sub-Saharan Africa). Cedar Falls, IA, USA: University of Northern Iowa; 2024. Available from: <https://scholarworks.uni.edu/grp/4610>.
37. Mahomed A, Pretorius C. Understanding the lived experiences of family caregivers of individuals with dementia in Soweto, a South African Township. Dementia. 2022;21(7):2264-87.
38. Paddick SM, Yoseph M, Gray WK, Andrea D, Barber R, Colgan A, et al. Effectiveness of app-based cognitive screening for dementia by lay health workers in low resource settings. A validation and feasibility study in rural Tanzania. Journal of Geriatric Psychiatry and Neurology. 2021;34(6):613-21.
39. Mwendwa P, Lawlor B, Kroll T, De Bru´n A. A systematic review and narrative synthesis of the experiences with caring for older people living with dementia in Sub-Saharan Africa. BMC geriatrics. 2022;22(1):961.
40. Ayisi-Boateng NK, Opoku DA, Tawiah P, Owusu-Antwi R, Konadu E, Apenteng GT, et al. Carers’ needs assessment for patients with dementia in Ghana. African Journal of Primary Health Care & Family Medicine. 2022;14(1):3595.
41. Kamoga R, Mubangizi V, Owokuhaisa J, Muwanguzi M, Natakunda S, Rukundo GZ. Behavioral and psychological symptoms of dementia: prevalence, symptom severity, and caregiver distress in South-Western Uganda—a quantitative

cross-sectional study. International Journal of Environmental Research and Public Health. 2023;20(3):2336.

42. Gumikiriza-Onoria JL, Nakigudde J, Mayega RW, Giordani B, Sajatovic M, Mukasa MK, et al. Psychological distress among family caregivers of persons with Alzheimer’s disease and related dementias in Uganda. BMC geriatrics. 2024;24(1):602.
43. Ikanga J, Reyes A, Zhao L, Hill-Jarrett TG, Hammers D, Epenge E, et al. Exploring factors contributing to caregiver burden in family caregivers of congolese adults with suspected dementia. International journal of geriatric psychiatry. 2023;38(9):e6004.
44. Owokuhaisa J, Kamoga R, Musinguzi P, Muwanguzi M, Natukunda S, Mubangizi V, et al. Burden of care and coping strategies among informal caregivers of people with behavioral and psychological symptoms of dementia in rural

south-western Uganda. BMC geriatrics. 2023;23(1):475.

45. Th´ebaut C, Houinato D, Gansou G, Gnonlonfoun D, Adoukonou T, Preux P, et al. Informal care time and costs of dementia care in Benin (West Africa). Neuroepidemiology. 2024.
46. Pokala S, Wandera SO, Kawooya S, Victoria AE, Odo LW, Nakasujja N, et al. Dementia knowledge and caregiver burden among carers of older adults in rural eastern Uganda. Alzheimer’s & Dementia. 2025;21(8):e70587.
47. Musyimi CW, Ndetei DM, Evans-Lacko S, Oliveira D, Mutunga E, Farina N. Perceptions and experiences of dementia and its care in rural Kenya. Dementia. 2021;20(8):2802-19.
48. Okwaraji FE, Onyebueke GC, Nwakaego OE. Burn Out and Psychological Distress among Care Givers of Dementia Patients Attending the Out Patient Geriatric Clinics of Two Nigerian Tertiary Health Institutions. International Neuropsychiatric Disease Journal. 2023;20(4):15-23.
49. Jacobs R, Schneider M, Farina N, du Toit P, Docrat S, Comas-Herrera A, et al. Dementia in South Africa: a situational analysis. Dementia. 2024;23(3):452-75.
50. Walker J, Dotchin C, Breckons M, Fisher E, Lyimo G, Mkenda S, et al. Patient and caregiver experiences of living with dementia in Tanzania. Dementia. 2023;22(8):1900-20.
51. Ainamani HE, Bamwerinde WM, Rukundo GZ, Tumwesigire S, Kalibwani RM, Bikaitwaho EM, et al. Participation in gardening activity and its association with improved mental health among family caregivers of people with dementia in rural Uganda. Preventive medicine reports. 2021;23:101412.
52. Stuart-R¨ohm K, Clark IN, Baker FA. ‘Caregivers’ Experiences of a Singing Training Program to Support Person-Centered Dementia Care. Journal of Music Therapy. 2024;61(3):244-62.
53. Musyimi CW, Muyela LA, Ndetei DM, Evans-Lacko S, Farina N. Acceptability and feasibility of a community dementia stigma reduction program in kenya. Journal of Alzheimer’s Disease. 2024;100(2):699-711.
54. Pedroza P, Miller-Petrie MK, Chen C, Chakrabarti S, Chapin A, Hay S, et al. Global and regional spending on dementia care from 2000–2019 and expected future health spending scenarios from 2020–2050: an economic modelling exercise. EClinicalMedicine. 2022;45.
55. Chen S, Cao Z, Nandi A, Counts N, Jiao L, Prettner K, et al. The global macroeconomic burden of Alzheimer’s disease and other dementias: estimates and projections for 152 countries or territories. The Lancet Global Health. 2024;12(9):e1534-43.
56. Akinyemi RO, Yaria J, Ojagbemi A, Guerchet M, Okubadejo N, Njamnshi AK, et al. Dementia in Africa: current evidence, knowledge gaps, and future directions. Alzheimer’s & Dementia. 2022;18(4):790-809.
57. Reid E, Ghoshal A, Khalil A, Jiang J, Normand C, Brackett A, et al. Out-of-pocket costs near end of life in low-and middle-income countries: a systematic review. PLOS Global Public Health. 2022;2(1):e0000005.

[Click here to access/download;Figure;Figure2.png](https://www2.cloud.editorialmanager.com/pgph/download.aspx?id=836930&guid=28f5e584-8c83-4234-b6da-ce1a22503b0e&scheme=1)[<https://www2.cloud.editorialmanager.com/pgph/download.aspx?id=836930&guid=28f5e584-8c83-4234-b6da-ce1a22503b0e&scheme=1>](https://www2.cloud.editorialmanager.com/pgph/download.aspx?id=836930&guid=28f5e584-8c83-4234-b6da-ce1a22503b0e&scheme=1)

[Click here to access/download;Figure;Figure3.png](https://www2.cloud.editorialmanager.com/pgph/download.aspx?id=836931&guid=7ebf55b1-fc94-498e-80db-4789b26ab19b&scheme=1)[<https://www2.cloud.editorialmanager.com/pgph/download.aspx?id=836931&guid=7ebf55b1-fc94-498e-80db-4789b26ab19b&scheme=1>](https://www2.cloud.editorialmanager.com/pgph/download.aspx?id=836931&guid=7ebf55b1-fc94-498e-80db-4789b26ab19b&scheme=1)

[Click here to access/download;Figure;Figure4.png](https://www2.cloud.editorialmanager.com/pgph/download.aspx?id=836932&guid=0872474f-e671-4645-a286-32557e3b0e96&scheme=1)[<https://www2.cloud.editorialmanager.com/pgph/download.aspx?id=836932&guid=0872474f-e671-4645-a286-32557e3b0e96&scheme=1>](https://www2.cloud.editorialmanager.com/pgph/download.aspx?id=836932&guid=0872474f-e671-4645-a286-32557e3b0e96&scheme=1)

[Click here to access/download;Figure;Figure5.png](https://www2.cloud.editorialmanager.com/pgph/download.aspx?id=836933&guid=b461d090-128c-41da-92cf-6e40199b8997&scheme=1)[<https://www2.cloud.editorialmanager.com/pgph/download.aspx?id=836933&guid=b461d090-128c-41da-92cf-6e40199b8997&scheme=1>](https://www2.cloud.editorialmanager.com/pgph/download.aspx?id=836933&guid=b461d090-128c-41da-92cf-6e40199b8997&scheme=1)

[Click here to access/download;Figure;map.png](https://www2.cloud.editorialmanager.com/pgph/download.aspx?id=836935&guid=1e03b065-5361-4593-abae-d70d25d633e6&scheme=1)[<https://www2.cloud.editorialmanager.com/pgph/download.aspx?id=836935&guid=1e03b065-5361-4593-abae-d70d25d633e6&scheme=1>](https://www2.cloud.editorialmanager.com/pgph/download.aspx?id=836935&guid=1e03b065-5361-4593-abae-d70d25d633e6&scheme=1)

[Click here to access/download;Figure;S1_Table.docx](https://www2.cloud.editorialmanager.com/pgph/download.aspx?id=836939&guid=e2da288d-15b3-46e4-ad01-1ef71b9c1af9&scheme=1)[<https://www2.cloud.editorialmanager.com/pgph/download.aspx?id=836939&guid=e2da288d-15b3-46e4-ad01-1ef71b9c1af9&scheme=1>](https://www2.cloud.editorialmanager.com/pgph/download.aspx?id=836939&guid=e2da288d-15b3-46e4-ad01-1ef71b9c1af9&scheme=1)

**S1 Table. Evidence extraction matrix of the 33 included studies on dementia care gaps in Sub-Saharan Africa.**

| **Study ID** | **Reference number** | **Study** | **Year** | **Country/Region** | **Study Design** | **Sample Size / Data Scope** | **Population Type** | **Thematic domain** | **Key Dementia Care Gaps / Findings** | **Barrier / Gap Category** |
| --- | --- | --- | --- | --- | --- | --- | --- | --- | --- | --- |
| 1 | [25] | Wakawa et al. | 2025 | Northeastern Nigeria | Retrospective hospital-based analysis | 1,216 dementia cases aged 60 years and older | Older adults diagnosed with dementia | Environmental and social challenges; Technology-related challenges | Documented dementia burden at the only neuropsychiatric facility in Northeastern Nigeria. Findings show dependence on limited specialized facilities and the need for  broader diagnostic and service capacity. | Specialist-service gap; diagnostic delay; facility concentration; data gap |
| 2 | [26] | Wisdom et al. | 2025 | Nigeria | Literature-based policy analysis | Literature and policy synthesis | People with neurodegenerative diseases, including dementia | Environmental and social challenges; Technology-related challenges | Identified inadequate infrastructure, few specialists, high care costs, cultural misconceptions, limited rural access, insufficient research funding, and lack of  epidemiological data. | Infrastructure gap; workforce shortage; cost barrier; research funding gap |
| 3 | [27] | Ssonko et al. | 2023 | South Africa | Retrospective memory-clinic cohort study | 165 memory-clinic patients aged 60 years and older | Older adults with dementia | Environmental and social challenges | Reported dementia subtypes, cognitive decline, and survival among memory-clinic patients. Findings indicate the need for longitudinal clinic data and earlier diagnosis  in African memory-clinic settings. | Memory-clinic capacity; late diagnosis; longitudinal data gap |
| 4 | [28] | Bosire et al. | 2025 | Kenya | Ethnographic qualitative study | 21 healthcare providers | Healthcare providers involved in AD/ADRD diagnosis and care | Environmental and social challenges; Community-based dementia care and informal caregiving challenges | Identified gaps in early detection due to lack of equipment, inadequate or untrained providers, high diagnosis and care costs, limited tools, stigma, specialist shortages,  and lack of clear care pathways. | Workforce gap; diagnostic-equipment gap; cost barrier; stigma; policy gap |
| 5 | [29] | Beyene and Berha | 2023 | Sub-Saharan Africa | Systematic review | 8 studies; 2,781 patients | People with dementia and HIV-associated dementia | Environmental and social challenges | Reviewed management practice and clinical outcomes. Cognitive stimulation therapy and early antiretroviral therapy showed benefits, but available intervention evidence remained  limited. | Treatment-evidence gap; intervention gap; low-resource care model |
| 6 | [30] | Alemayehu et al. | 2025 | Sub-Saharan Africa | Narrative literature review | Literature synthesis | People living with dementia in SSA | Technology-related challenges; Financial and economic challenges | Discussed risk factors, public perception, and management approaches. Highlighted myths, stigma, data drought, limited diagnosis, and the need for national dementia plans and  culturally adapted interventions. | Data gap; stigma; diagnostic gap; policy gap; culturally adapted care |
| 7 | [31] | Oyinlola | 2024 | Nigeria | Policy analysis and literature-based review | Policy and literature synthesis | People living with dementia and caregivers | Technology-related challenges | Argued that Nigeria lacks a coherent national dementia strategy. Highlighted stigma, weak caregiver support, limited information  systems, and the need to integrate dementia into health and social care systems. | Policy gap; national-strategy gap; stigma; caregiver support; information systems |
| 8 | [32] | Musyimi et al. | 2023 | Kenya | Qualitative vignette and stakeholder care-pathway study | 29 stakeholders | Stakeholders and community dementia-care pathway actors | Technology-related challenges | Identified four initial care responses: perceiving symptoms as normal aging, consulting spiritual or traditional healers,  visiting private or primary care, or taking no action. | Care-pathway gap; cultural belief; delayed care; awareness gap |
| 9 | [33] | Mwendwa et al. | 2021 | Kenya | Interpretive phenomenological qualitative study | 10 caregivers | Family caregivers of people living with dementia | Technology-related challenges | Caregivers described dementia care as stressful and continuous, with emotional,  financial, practical, and knowledge-related challenges. | Caregiver burden; financial stress; knowledge gap; support-service gap |
| 10 | [34] | Aballa et al. | 2025 | Kenya | Ethnographic qualitative study | 5 focus-group discussions  with 35 participants; additional interviews NR | Rural community members | Technology-related challenges | Found that older people with dementia may be perceived as witches and exposed to stigma or harm. Highlights the need for  public education and protection. | Stigma; cultural misconception; community safety; awareness gap |
| 11 | [35] | Yenesew et al. | 2024 | East Africa | Scoping review of population-based studies | 7 included studies after screening 445 publications | Older adults in East Africa | Technology-related challenges | Found very limited population-based evidence, including four studies on dementia prevalence and three on cognitive impairment. Called for more longitudinal and population-based research and stronger  regional evidence generation. | Epidemiological data gap; research-capacity gap; regional evidence gap |
| 12 | [36] | Aryee | 2024 | Ghana | Systematic review of adult day-center literature | Literature on adult day-center models | People with dementia in Ghana | Technology-related challenges | Reviewed the need for culturally appropriate adult day centers. Identified stigma, lack of infrastructure, caregiver unpreparedness, and  lack of respite services. | Formal-care infrastructure gap; respite-care gap; stigma; caregiver training |
| 13 | [37] | Mahomed and Pretorius | 2022 | South Africa | Qualitative study | NR | Family caregivers of individuals with dementia | Technology-related challenges | Explored lived experiences of dementia caregiving in a township context. Findings  highlight emotional, social, practical, and support-service challenges for caregivers. | Caregiver burden; township service gap; psychosocial support gap |
| 14 | [38] | Paddick et al. | 2021 | Tanzania | Validation and feasibility  study | 3,011 screened; 610  clinically assessed | Older adults aged 60  years and older in rural | Technology-related challenges | Tested app-based dementia screening by lay  health workers. Demonstrated the potential of | Technology gap; workforce  shortage; screening accessibility |

| **Study ID** | **Reference number** | **Study** | **Year** | **Country/Region** | **Study Design** | **Sample Size / Data Scope** | **Population Type** | **Thematic domain** | **Key Dementia Care Gaps / Findings** | **Barrier / Gap Category** |
| --- | --- | --- | --- | --- | --- | --- | --- | --- | --- | --- |
|  |  |  |  |  |  |  | Tanzania |  | digital screening to address specialist  shortages in low-resource settings. |  |
| 15 | [39] | Mwendwa et al. | 2022 | Sub-Saharan Africa | Systematic review and narrative synthesis | 19 studies; 378 caregivers | Family and informal caregivers | Technology-related challenges | Synthesized six themes: conceptualizing dementia, caregiving arrangements, caregiving impact, caregiver identity, managing care, and unmet needs. Called for better information campaigns and support  programs. | Informal-care burden; unmet support needs; caregiver training gap |
| 16 | [40] | Ayisi-Boateng et al. | 2022 | Ghana: Kumasi | Multicentre cross-sectional needs-assessment study | 50 carers | Adult carers of people previously diagnosed with dementia | Community-based dementia care and informal caregiving challenges | Identified substantial unmet caregiver needs, particularly for clear information about dementia, treatment, and crisis management. Findings support accessible education, counselling, and practical caregiver  assistance. | Caregiver-information gap; unmet support needs; caregiver-training gap |
| 17 | [41] | Kamoga et al. | 2023 | Uganda: rural Southwestern Uganda | Population-based quantitative cross-sectional study | 175 caregivers of people living with dementia | Family caregivers of people living with dementia | Community-based dementia care and informal caregiving challenges | Almost all caregivers reported at least one behavioral or psychological symptom of dementia. Frequent and severe symptoms caused substantial caregiver distress, demonstrating the need for early BPSD  screening and caregiver support. | BPSD-management gap; caregiver distress; early-screening gap; psychosocial-support gap |
| 18 | [42] | Gumikiriza-Onoria et al. | 2024 | Uganda: Wakiso District | Cross-sectional caregiver mental-health study using purposive sampling | 90 family caregivers selected from a pre­existing cohort of 500 individuals | Family caregivers of people with Alzheimer’s disease and related dementias | Community-based dementia care and informal caregiving challenges | Psychological distress and depression each affected 64.4% of caregivers. Findings demonstrate the need for structured mental-health services, caregiver-support systems,  and gender-responsive interventions. | Caregiver mental-health burden; depression; formal-support gap; gender-responsive care gap |
| 19 | [43] | Ikanga et al. | 2023 | Democratic Republic of the Congo | Pilot cross-sectional caregiver-burden study | 30 patient-caregiver dyads | Family caregivers of adults with suspected dementia | Community-based dementia care and informal caregiving challenges | Caregiver burden was associated with care-recipient cognitive impairment, functional decline, frailty, depression, agitation, and other neuropsychiatric symptoms. Findings  highlight the need for family support and symptom-management services. | Caregiver burden; neuropsychiatric-symptom management gap; family-support gap |
| 20 | [44] | Owokuhaisa et al. | 2023 | Uganda | Cross-sectional qualitative study using purposive sampling, in-depth interviews, and thematic content analysis | 27 informal caregivers | Informal caregivers of older people with behavioral and psychological symptoms of dementia (BPSD) | Community-based dementia care and informal caregiving challenges | Caregivers experienced substantial financial, physical, psychological, and social burdens. Common coping strategies included religious coping, acceptance, emotional and social  support, planning, persistence, and self-distraction. | Caregiver burden; financial strain; psychosocial burden; limited caregiver and health-system support |
| 21 | [45] | Thébaut et al. | 2024 | Benin | Cost-of-illness study using hospital and community recruitment | 135 older individuals with varying dementia stages | Older adults with dementia and informal caregivers | Community-based dementia care and informal caregiving challenges | Caregivers provided up to eight hours of care daily. Mean annual dementia-care costs were approximately PPP$2,399.66, and informal  care represented up to 92% of total costs. | Informal-care burden; time burden; financial burden; economic-data gap |
| 22 | [46] | Pokala et al. | 2025 | Uganda: rural Eastern Uganda | Cross-sectional caregiver-older-adult dyadic survey | 602 caregiver-older-adult  dyads interviewed; 598 matched dyads included in the final analysis | Carers of older adults in rural communities | Community-based dementia care and informal caregiving challenges | Caregiver burden prevalence was 28.9%. In adjusted analyses, greater dementia knowledge and caregiver depression were associated with caregiver burden; among caregivers of older adults with dementia,  caregiver depression and older caregiver age were associated with burden. | Caregiver burden; caregiver mental-health gap; dementia-education gap; rural-support gap |
| 23 | [47] | Musyimi et al. | 2021 | Kenya: rural communities | Qualitative study using focus-group discussions and individual interviews | 38 key stakeholders in focus groups plus 5 individual caregiver interviews | Carers, healthcare providers, and members of the general public | Community-based dementia care and informal caregiving challenges | Identified negative stereotypes, limited dementia knowledge, fragmented diagnostic pathways, neglect, and abuse. Poor awareness and inadequately equipped healthcare systems reinforced stigma and  delayed care. | Cultural misconception; stigma; knowledge gap; diagnostic-pathway gap |
| 24 | [48] | Okwaraji et al. | 2023 | Nigeria: Enugu, Southeast Nigeria | Descriptive cross-sectional study | 460 caregivers | Caregivers of dementia patients attending two tertiary geriatric clinics | Community-based dementia care and informal caregiving challenges | Psychological distress affected 39.6% of caregivers, while 23.9% experienced emotional exhaustion. Findings support  institutional assistance, caregiver incentives, and community caregiving centres. | Caregiver burnout; psychological distress; institutional-support gap; financial support need |
| 25 | [49] | Jacobs et al. | 2024 | South Africa | Three-phase national situational analysis | Desk review; multisectoral stakeholder interviews; and SWOT analysis | People living with dementia, families, and service providers | Community-based dementia care and informal caregiving challenges | Identified structural barriers to dementia diagnosis, care, and support, along with gaps and opportunities in existing service provision. The study emphasized the need for  coordinated intersectoral policy responses. | Health-system fragmentation; policy gap; diagnostic gap; service-access gap |
| 26 | [50] | Walker et al. | 2023 | Tanzania | Qualitative study using semi-structured | 14 people with dementia and 12 caregivers; 26 | People living with dementia and their | Community-based dementia care and informal caregiving challenges | Participants described social isolation, stigma, cultural misconceptions, limited | Stigma; caregiver-knowledge gap; formal-care gap; service- |

| **Study ID** | **Reference number** | **Study** | **Year** | **Country/Region** | **Study Design** | **Sample Size / Data Scope** | **Population Type** | **Thematic domain** | **Key Dementia Care Gaps / Findings** | **Barrier / Gap Category** |
| --- | --- | --- | --- | --- | --- | --- | --- | --- | --- | --- |
|  |  |  |  |  | interviews and framework analysis | participants total | caregivers |  | formal healthcare, and insufficient caregiver knowledge and support. These challenges affected the physical and mental health of  people with dementia. | access gap |
| 27 | [51] | Ainamani et al. | 2021 | Uganda | Quantitative cross-sectional survey study | 242 family caregivers; 131 participated in gardening and 111 did not | Family caregivers of older people living with dementia who had provided care for more  than six months | Community-based dementia care and informal caregiving challenges | Caregivers reported substantial burden and high levels of depression, anxiety, and stress. Participation in gardening was associated with lower symptoms of depression, anxiety,  and stress. | Caregiver mental-health burden; psychosocial-support gap; community-based intervention gap |
| 28 | [52] | Stuart-Röhm et al. | 2024 | South Africa | Participatory action-research study using iterative training cycles and qualitative interviews | 8 caregivers across 2 care  homes; 4 iterative participatory cycles | Formal caregivers of people living with dementia | Community-based dementia care and informal caregiving challenges | Person-centered caregiver-singing training increased dementia knowledge, emotional well-being, confidence, self-awareness, and self-efficacy. Findings highlight the need for  sustainable and scalable caregiver training. | Caregiver-training gap; psychosocial-intervention gap; person-centered-care gap |
| 29 | [53] | Musyimi et al. | 2024 | Kenya | Community intervention feasibility study | NR | Community members and dementia-care stakeholders | Community-based dementia care and informal caregiving challenges | Examined acceptability and feasibility of a community dementia stigma-reduction program. Highlights the importance of community-level education to reduce stigma  and improve care-seeking. | Stigma; awareness gap; community intervention gap |
| 30 | [54] | Pedroza et al. | 2022 | Sub-Saharan Africa (regional estimates within a global analysis) | Global and regional economic modelling study based on a  systematic review of dementia-care spending | Dementia spending estimates for 2000-2019; projected health-spending scenarios for 2020-2050 | People living with dementia and health-financing systems | Financial and economic challenges | Estimated dementia-attributable spending in SSA at US$552 million in 2019, projected to rise to US$6,955 million by 2050. Reliance  on modelled estimates highlights the scarcity of primary dementia-cost data from SSA. | Economic-data gap; primary-cost-data gap; health-financing gap; rising expenditure burden |
| 31 | [55] | Chen et al. | 2024 | Sub-Saharan Africa (regional estimates within a global analysis) | Health-augmented macroeconomic modelling study | 152 countries or territories; economic-burden estimates and projections for 2020-2050 | People living with dementia, informal caregivers, and national economies | Financial and economic challenges | Estimated the economic burden of  Alzheimer’s disease and other dementias in SSA at approximately 158 billion international dollars during 2020-2050. The model incorporated morbidity, mortality, formal-care costs, and reductions in  caregivers’ labor supply. | Macroeconomic burden; informal-care productivity loss; indirect-cost data gap; financing and planning gap |
| 32 | [56] | Akinyemi et al. | 2022 | Africa | Narrative evidence review | Review of African dementia evidence and knowledge gaps | People living with dementia in Africa | Financial and economic challenges | Reported wide variation in dementia prevalence and major knowledge gaps. Highlighted the need for longitudinal studies, culturally sensitive cognitive tools, biomarkers, genomics, coordinated African  dementia research capacity, and better cost evidence. | Research gap; diagnostic-tool gap; biomarker gap; data gap; economic-data gap |
| 33 | [57] | Reid et al. | 2022 | Low- and middle-income countries; included Kenya and Tanzania among country examples | Systematic review | 12 included studies after screening 9,343 records | Adults near end of life or with serious life-limiting illness, including advanced  Alzheimer’s disease and related dementias; patients, families, and caregivers | Financial and economic challenges | Reported that out-of-pocket costs increase with advanced illness and disease severity. Main cost drivers included medications and hospitalizations. High costs were associated with financial catastrophe, lost income, debt/borrowing, foregone education, and pressure on households. The review highlights the need for financial protection  policies and further research on palliative and end-of-life care economics in LMICs. | Out-of-pocket financing; catastrophic health expenditure; financial-protection gap; palliative/end-of-life care financing gap; household economic burden |

*Note. NR = not reported or not clearly available in the extracted material. Reference numbers correspond to the manuscript reference list.*

[Click here to access/download;Figure;Figure1.png](https://www2.cloud.editorialmanager.com/pgph/download.aspx?id=836929&guid=bf1a327b-6d28-4b2a-95de-3a7150b0f3c0&scheme=1)[<https://www2.cloud.editorialmanager.com/pgph/download.aspx?id=836929&guid=bf1a327b-6d28-4b2a-95de-3a7150b0f3c0&scheme=1>](https://www2.cloud.editorialmanager.com/pgph/download.aspx?id=836929&guid=bf1a327b-6d28-4b2a-95de-3a7150b0f3c0&scheme=1)

PRISMA-Sc

[Click here to access/download](https://www2.cloud.editorialmanager.com/pgph/download.aspx?id=836945&guid=7968083b-08f2-441e-ae39-5df014fc3916&scheme=1)

# [Supporting Information](https://www2.cloud.editorialmanager.com/pgph/download.aspx?id=836945&guid=7968083b-08f2-441e-ae39-5df014fc3916&scheme=1)

[PRISMA-ScR-Fillable-Checklist_10Sept2019.docx](https://www2.cloud.editorialmanager.com/pgph/download.aspx?id=836945&guid=7968083b-08f2-441e-ae39-5df014fc3916&scheme=1)

[Click here to access/download](https://www2.cloud.editorialmanager.com/pgph/download.aspx?id=836936&guid=dcaac6e7-bb7b-41f6-b1e6-0c2cd83abe69&scheme=1)

[**Supporting Information** plos2025.bst](https://www2.cloud.editorialmanager.com/pgph/download.aspx?id=836936&guid=dcaac6e7-bb7b-41f6-b1e6-0c2cd83abe69&scheme=1)

[Click here to access/download](https://www2.cloud.editorialmanager.com/pgph/download.aspx?id=836937&guid=7f1853f3-ed29-4020-9883-0e7397760423&scheme=1)

[**Supporting Information** plos_bibtex_sample.bib](https://www2.cloud.editorialmanager.com/pgph/download.aspx?id=836937&guid=7f1853f3-ed29-4020-9883-0e7397760423&scheme=1)

[Click here to access/download](https://www2.cloud.editorialmanager.com/pgph/download.aspx?id=836938&guid=f91cc5d4-a91d-4171-ba68-bf4715922918&scheme=1)

[**Supporting Information** plos_latex_template.pdf](https://www2.cloud.editorialmanager.com/pgph/download.aspx?id=836938&guid=f91cc5d4-a91d-4171-ba68-bf4715922918&scheme=1)

Revised Manuscript with Track Changes

## Dementia Care Gaps in Sub-Saharan Africa: A Scoping Review of Technology, Environmental, Community Perspectives, and Financial Constraints

Soarov Chakra Borty^1^, Maiya G. Block Ngaybe^2^, Angela E Apeagyei^3^, Divya Bhagianadh^4^, Monica H Swahn^5^, Nazmus Sakib^1^

1 Department of Information Technology, Kennesaw State University, Kennesaw, Georgia, United States
2 School of Landscape Architecture, College of Architectural Planning and Landscape Architecture, University of Arizona, Tucson, Arizona, United States
3 Department of Health Metrics Sciences, School of Medicine, University of Washington, Seattle, Washington, United States
4 Department of Health, Human Performance, & Recreation, University of Arkansas, Fayetteville, Arkansas, United States
5 School of Public Health, Virginia Commonwealth University, Richmond, Virginia, United States

*[mgblock@arizona.edu](mailto:mgblock@arizona.edu)

## Abstract

Dementia is a growing public health challenge in Sub-Saharan Africa (SSA), where population aging is expected to substantially increase the regional burden of dementia in the coming decades. Rapidly changing demographics, underdeveloped health infrastructure, and limited awareness of dementia symptoms and care pathways contribute to delayed diagnosis, treatment, and care. This scoping review examined literature published from 2021 to 2025 to identify gaps in dementia care across four key domains: environmental and social factors, technological capacity, community-based caregiving, and financial and economic factors. First, social and ecological factors, including low health literacy, rural residence, and weak primary care infrastructure, influence disease diagnosis and access to care. Second, technological capacity remains limited, including scarce access to neuroimaging and diagnostic tools, limited telemedicine coverage, weak electronic health information systems, and insufficient digital health solutions for early detection, management, and monitoring. Third, evidence from community-based care highlights heavy reliance on informal caregivers who often lack training and institutional support, resulting in substantial emotional, physical, and financial burden. Finally, inadequate public financing for dementia care, coupled with limited insurance coverage, further restricts access to dementia care services and support. Overall, this review highlights critical gaps in policy, evidence generation, cost-effective technology investment, caregiver support, and health systems strengthening across all levels of care. Addressing these multidimensional challenges is crucial to improving the quality of life for people living with dementia and their caregivers across SSA.

## 1 Introduction

Dementia is an emerging public health concern in SSA due to epidemiological and demographic transitions, as well as significant inequities in access to diagnostic services

and long-term care services [1–6]. People living with dementia (PLWD) is projected to increase from 2.13 million in 2015 to 3.48 million by 2030 and 7.62 million by 2050 in SSA, although they currently account for a smaller share of global dementia cases than in high-income countries [7–9]. This relatively low reported case count is likely due to a high number of missed diagnoses, limited access to neuroimaging and cognitive assessment tools, shortages of trained dementia specialists, and weak referral pathways. Dementia in SSA remains broadly overlooked across international research, surveillance efforts, and policy agendas, despite these trends [10, 11]. Caregiving for PLWD is almost entirely the responsibility of informal caregivers (primarily women) in these settings, as in other low and middle-income countries (LMIC). Resource constraints and minimal healthcare infrastructure exacerbate the burden of dementia and limit access to formal care services. While health expenditure remains generally low at the national level across all LMICs, SSA bears the lowest health spending of any region in the world. In 2021, countries in SSA spent an estimated USD 92 per capita on health [12], compared with USD 379 per capita in North Africa and the Middle East, which ranked next lowest at the time. This disparity partly explains the strong reliance on informal caregivers seen in LMIC settings. Unpaid informal caregiving (65% of total costs) is the most significant contributor to the overall financial burden of dementia in LMICs, and most countries fail to offer any support to informal carers [11].

Since the 1990s, many SSA countries have experienced increasing life expectancy, with a focus on maternal and child health, infectious disease control, and primary health care [13]. As a result, the phenomenon of population aging has appeared more quickly than in previous generations, which has brought attention to the problem of dementia and other age-related health issues. In this regard, the problem of dementia and research on aging can be considered an increasingly important public health issue, which has brought a timely opportunity to develop and expand models of dementia care and research tailored to the aging population of SSA. Dementia in sub-Saharan Africa has historically received limited attention within health systems and policy agendas, though recent national and international efforts point to increasing engagement with dementia-related challenges. Recent studies from Ethiopia, Ghana, Kenya, Nigeria, South Africa, and Tanzania have examined dementia-related care gaps in SSA [14–18]. Together, these studies show that dementia care in SSA is shaped by sociocultural beliefs, limited awareness, reliance on family caregiving, broader health-system constraints, and gaps in diagnostic and financial support. Socioeconomic deprivation is also relevant to dementia in SSA, as evidence from South Africa shows an association between multidimensional poverty and dementia among adults aged 50 years or

older [19]. Families often face numerous challenges, including out-of-pocket costs, time spent on healthcare visits, transportation costs, assistive services availability, loss of leisure time, and psychosocial strain among caregivers [17, 20]. Coupled with limited health insurance coverage and weak social welfare systems, these pressures often lead to catastrophic health expenditures [18, 21], where households spend more than 10% of their household income on health care. Despite their importance, comprehensive and systematically quantified assessments of these technological, financial, and economic burdens remain limited in SSA [19–21].

Dementia care in SSA is characterized by intersecting challenges in research capacity, workforce development, and policy environments. This review examines the existing evidence on technological gaps, financial and economic barriers, and structural, clinical, and policy-related barriers to early diagnosis in SSA. Comprehensive dementia care encompasses numerous layers of the healthcare system, including technology, workforce capacity, community support, and financial protection. The four thematic domains used in this review are environmental and social challenges, technology-related challenges, community-based dementia care and informal caregiving challenges, and financial and

economic challenges, allowing gaps to be examined across prevention, diagnosis, care delivery, and long-term support. Together, these classifications are utilized as a systems-based organizing structure to frame the gap analysis, consistent with the WHO health system framework [22]. This systems-based structure supports cross-domain integration. This review focuses on studies/papers published between 2021 and 2025; earlier landmark analyses and global reports are cited to provide epidemiological context and conceptual framing.

In line with the World Health Organization (WHO) health system building blocks framework that views healthcare systems through the lens of six interrelated components: service delivery, health workforce, health information systems, medical products and technologies, financing, and leadership and governance, this review takes on the systems-based approach to provide insights on gaps in dementia care provision in SSA [22, 23]. This paper adds to the literature by providing a comprehensive review across multiple domains relevant to dementia care in SSA, with a focus on more recent studies. This review synthesizes the existing literature to identify research gaps and explore reported health care technologies to address issues across various aspects of the health system, including delayed diagnosis, inadequate care coordination, and

family-based caregiving.

## 2 Methods

### 2.1 Study Design and Reporting Framework

This study was conducted as a scoping review to map recent evidence on dementia care gaps for people living with dementia (PLWD) in SSA. A scoping review was appropriate because the evidence includes diverse clinical, health-system, policy, caregiving, and technology-related studies. The review followed established scoping review methodology and was reported according to the Preferred Reporting Items for Systematic Reviews and Meta-Analyses extension for Scoping Reviews (PRISMA-ScR) [24].

### 2.2 Information Sources and Search Strategy

A literature search was conducted in PubMed, Scopus, ScienceDirect, IEEE Xplore, and Google Scholar to identify studies on dementia care gaps in SSA. These databases were selected to capture multidisciplinary evidence from biomedical, public health, social science, health systems, and technology-related research. The search was limited to studies published between January 2021 and December 2025. The main Boolean search string combined terms related to dementia, geography, and care gaps: (”dementia” OR ”Alzheimer’s disease” OR ”cognitive impairment”) AND (”Sub-Saharan Africa” OR ”SSA” OR ”Africa”) AND (”care” OR ”caregiving” OR ”health system” OR ”healthcare access” OR ”diagnosis” OR ”treatment” OR ”technology” OR ”digital health” OR ”cost” OR ”financing”). Search terms were adapted as needed to match each database’s search functionality.

Google Scholar was used as a supplementary source to identify relevant grey literature and articles not indexed in the main databases. In addition, website searches were conducted using relevant organizational and public health sources, including the World Health Organization, Alzheimer’s Disease International, and dementia or public health websites relevant to SSA. Citation tracking of key included articles was also performed to identify additional eligible studies.

### 2.3 Eligibility Criteria

The studies were selected based on predetermined inclusion and exclusion criteria.

#### Inclusion criteria:

i. Studies related to dementia, Alzheimer’s disease, and other cognitive impairments.
ii. Studies conducted in, or relevant to, countries within SSA.
iii. Studies relevant to dementia care, caregiving, healthcare access, workforce issues, policy, health-system factors, or technology-related interventions.
iv. Original research articles, review articles, and studies published within the period 2021–2025.

#### Exclusion criteria:

i. Studies not relevant to dementia or cognitive impairments.
ii. Studies not relevant to SSA.
iii. Studies not relevant to the scope of dementia care and healthcare system issues.

**Fig 1.** Flow diagram of literature search and screening for dementia care gaps in SSA.

iv. Editorials, commentaries, opinion articles, letters, and papers without substantive analytical or evidence-synthesis content were excluded; systematic reviews, scoping reviews, narrative reviews, and policy analyses were included if they directly addressed dementia care gaps in SSA.

### 2.4 Study Selection Process

All records retrieved from databases and other sources were imported into a reference management file, and duplicate records were removed prior to screening. Study selection was conducted in two stages. First, four reviewers independently screened the titles and abstracts of all retrieved records against the predefined eligibility criteria.

Second, the full texts of potentially eligible studies were independently assessed by the same four reviewers to determine final inclusion. Inter-reviewer agreement among the four reviewers was assessed using Fleiss’ kappa statistic. Any disagreement during title/abstract or full-text screening was resolved through discussion and consensus. If consensus could not be reached, a fifth reviewer was consulted to make the final decision. Figure 1 illustrates the PRISMA flow diagram, which outlines the study identification, screening, eligibility, and inclusion process for the recent literature on dementia care gaps in SSA.

### 2.5 Synthesis of Results

The extracted data were synthesized and categorized using descriptive and thematic analysis to establish the status of dementia care in SSA. In this case, the gaps identified in four major areas, which include health systems capacity gaps, caregiving support gaps, service delivery and access gaps, and technology and innovation gaps, are targeted. The data synthesized is categorized under thematic areas, which include health system capacity gaps such as financial, economic, environmental, and social constraints, caregiving gaps such as training and support for informal and community-based caregivers, service delivery and access gaps such as fragmented care processes and access to care, and technology and innovation gaps such as the use and availability of technology in dementia diagnosis, care, and patient monitoring. A detailed evidence extraction matrix of the included studies is provided in S1 File.

## 3 Environmental and social challenges

This section focuses on the environmental, social, and systemic drivers that shape dementia diagnosis and management across Sub-Saharan Africa. Table 1 shows that 5 of the 33 included studies examined environmental and social challenges, with evidence primarily drawn from Nigeria, Kenya, South Africa, and broader SSA contexts. Based upon a retrospective analysis from Northeastern Nigeria in which Wakawa et al. [25] examined over two decades of patient records from the region’s sole neuropsychiatric facility, 1,216 cases of dementia among adults aged 60 years and older were documented. Alzheimer’s disease (60.5%) and vascular dementia (24.5%) were the most prevalent subtypes, with hypertension (41.6%) as the leading comorbidity. There was a limited diagnostic capacity due to a lack of neuroimaging tools, biomarkers, and a specialized workforce. This reflects the broader environmental and infrastructural constraints of dementia care in resource-limited regions. The authors emphasize that both vascular comorbidities and environmental stressors, such as chronic hypertension and

conflict-related trauma, may contribute to the region’s escalating dementia rates, underscoring the need for policy reforms that strengthen neurological health infrastructure and surveillance systems.

**Table 1.** Summary of included studies by thematic domain

| **Thematic domain** | **Number of studies** | **Main countries or regions represented** |
| --- | --- | --- |
| Environmental and social challenges | 5 | Nigeria, Kenya, South Africa, and broader SSA evidence |
| Technology-related chal­lenges | 12 | Nigeria, Kenya, Tanzania, Ghana, South Africa, East Africa, and broader SSA evidence |
| Community-based dementia care and informal caregiving challenges | 15 | Kenya, Uganda, Ghana, Benin, Nigeria, South Africa, Tanzania, the Demo­cratic Republic of the Congo, and broader SSA evidence |
| Financial and economic challenges | 5 | SSA-level, Africa-level, and global/regional modeling evidence including SSA |

Note: Studies could contribute to more than one thematic domain; therefore, the total number across domains exceeds 33.

Similarly, Wisdom et al. [26] identified environmental and occupational exposures as important but often overlooked contributors to neurodegenerative disease in Nigeria, including fine particulate air pollution, pesticide exposure, and groundwater contamination with heavy metals. Their analysis estimated that dementia prevalence has risen by nearly 400% over two decades, disproportionately affecting aging and

low-income populations. The authors argue for integrating dementia prevention into environmental health policies, noting that Nigeria’s current health governance lacks coherent frameworks linking neurodegenerative disease to ecological regulation or

non-communicable disease (NCD) control. Moreover, the concentration of neurologists and diagnostic centers in urban areas amplifies rural inequities and delays in diagnosis.

In South Africa, Ssonko et al. [27] conducted the first longitudinal analysis of memory clinic data from Groote Schuur Hospital in Cape Town, revealing that Alzheimer’s disease (70.9%) and vascular dementia (14.6%) accounted for the majority of 165 dementia cases. The study found that cognitive decline averaged 2.1 Mini-Mental State Examination (MMSE) points per year for Alzheimer’s and 1.3 for vascular dementia. Educational attainment and cardiovascular comorbidities, linked to environmental and lifestyle factors such as diet and urbanization, emerged as significant predictors of both cognitive decline and mortality. The study situates these findings within broader socioeconomic and infrastructural determinants, arguing that the limited formalized dementia registries and geriatric services in SSA hinder effective governance and continuity of care.

Beyond environmental stressors, social and cultural determinants deeply influence dementia risk, diagnosis, and management across SSA. Bosire et al. [28] conducted ethnographic research at the Aga Khan University Hospital in Nairobi, documenting healthcare providers’ perspectives on dementia diagnosis and management in Kenya. Although tertiary private hospitals offered multidisciplinary, globally aligned diagnostic pathways, providers highlighted the severe inequities between the private and public sectors. Most Kenyans lack access to neuroimaging, cognitive assessments, and specialized care from trained professionals. Stigma, misconceptions linking dementia to witchcraft or punishment, and the absence of a national dementia policy were recurrent themes. These sociocultural barriers delay care-seeking, limit early detection, and increase the burden on caregivers. The authors advocate for Kenya’s first national dementia strategy to bridge clinical, cultural, and governance divides.

In Nigeria, Wisdom et al. [26] further illuminated how gender, education, and social position intersect with stigma to shape the lived experience of dementia. Older women,

**Fig 2. Environmental and social risk factors affecting dementia diagnosis in SSA.** Dementia diagnosis in SSA is shaped by interacting environmental, social, and structural constraints, including limited access to neuroimaging and trained specialists, as well as pronounced rural–urban inequities in diagnostic services.^1,2^ Sociocultural misconceptions, stigma, and the absence of formal dementia policy frameworks further delay care-seeking and hinder early detection.^3^ **Notes:** ^1^ Diagnostic capacity and specialist workforce limitations in resource-constrained settings [25, 27]. ^2^ Environmental and occupational exposures linked to neurodegenerative risk [26]. ^3^ Sociocultural barriers, stigma, and policy absence affecting dementia recognition and diagnosis [28].

in particular, face discrimination and violence due to widespread beliefs that cognitive decline results from witchcraft. Informal caregivers, predominantly women, bear over 70% of unpaid care hours, often without institutional or financial support. These findings highlight important governance and policy implications, suggesting that dementia should be framed not only as a medical condition but also as a social issue with relevance to aging and gender equity policies.

While pharmacological interventions remain limited, psychosocial and behavioral strategies have shown promise for addressing this issue. Beyene et al. [29] conducted the first systematic review of dementia management practices in SSA, analyzing randomized clinical trials and cohort studies across eight countries. They found that Cognitive Stimulation Therapy (CST), a low-cost, group-based behavioral intervention, was feasible and effective in improving cognitive and language outcomes in low-resource settings. Furthermore, early initiation of combination antiretroviral therapy was associated with improved cognitive outcomes among individuals with HIV-associated dementia (HIVAD). The review also revealed systemic gaps, including a lack of culturally validated diagnostic tools, inadequate training of community health workers, and limited support networks for caregivers. The authors call for scaling up CST and integrating dementia management into primary healthcare systems through

task-shifting and training initiatives. These findings align with Wakawa et al. [25], who advocate for decentralized care models to extend dementia services beyond tertiary facilities. The establishment of the Northern Nigeria Dementia Research Group marks a significant step toward creating a national dementia registry and promoting the translation of research into policy and practice. Both studies emphasize the importance of governance structures that facilitate early detection, community-based care, and the sharing of regional data. Figure 2 summarizes the environmental, cultural, and social factors contributing to disparities in dementia care across SSA.

Collectively, these studies demonstrate that dementia in SSA is shaped by

intersecting environmental, social, and systemic factors that demand an integrated governance response. Three broad policy imperatives emerge. First, it is essential to integrate dementia into the National Health Frameworks and plans. Dementia should be formally embedded within national aging, NCD, and environmental health policies.

Governments can align with the WHO Global Action Plan on the Public Health Response to Dementia (2017–2025) by developing country-specific dementia strategies, establishing registries, and promoting caregiver protections. Secondly, capacity building and decentralization of efforts are key. Investment in workforce training, particularly in task-shifting, primary care, and community health workers, is essential. There is also evidence that this approach, supported by findings from Ethiopia, Nigeria, and Kenya, can bridge diagnostic and treatment gaps in rural regions. Finally, cross-sectoral and international collaboration is essential. Effective governance needs to extend beyond the health sector to address the broader social and environmental determinants of dementia. Integration with ecological regulation (air quality, pesticide control), education, and social welfare systems is key. Furthermore, partnerships between African governments, research consortia, and global funding bodies can support evidence-based policymaking and the development of research infrastructure.

The growing body of dementia research in SSA provides critical insights into how environmental exposures and social inequities converge to shape cognitive aging. Across Nigeria, Kenya, South Africa, and Ethiopia, recent evidence suggests the need for urgent governance reforms that integrate environmental stewardship, social protection, and health system strengthening. By reframing dementia as both a public health and a social governance challenge, SSA can lead the development of a new, integrated, and equity-centered global health policy model for the prevention and care of neurodegenerative diseases.

## 4 Technology-related challenges

Digital health technologies can enhance dementia care in low-resource settings; however, their adoption across SSA remains minimal due to infrastructural, financial, and training limitations. As shown in Table 1, technology-related challenges were reported in 12 of the 33 included studies and were represented across Nigeria, Kenya, Tanzania, Ghana, South Africa, East Africa, and wider SSA evidence. Internet connectivity is inconsistent and often unavailable in rural areas, and many healthcare facilities lack a reliable power supply and the necessary hardware to conduct telemedicine and remote consultations [30, 31]. Telemedicine, tele-support for caregivers, has been investigated as one of the solutions for extending care for dementia patients, but the development of this type of service in SSA is uneven due to problems with information technology infrastructure. The systematic reviews of the effects of telemedicine in low- and middle-income countries, including SSA, suggest the potential of telemedicine to improve access to specialist consultation and caregiver support, while also identifying persistent barriers related to infrastructure reliability, workforce training, and integration with existing health systems. One key area that could be improved by applying technological advancements to SSA’s support structure for dementia is standardization and greater accessibility of screening. With so many different cultural settings and linguistic patterns across SSA, a lack of standardized screening methods could merely reflect a scarcity of understanding and preparation within healthcare.

Frontline health workers in many countries misclassify dementia symptoms with “normal aging,” “stress,” or general cognitive decline as a person gets older [32, 33].

Importantly, efforts to improve dementia screening and diagnosis in SSA should not assume that dementia screening tools and care pathways can be directly transferred without local adaptation. Local beliefs about aging, memory loss, family responsibility,

and what constitutes normal versus pathological cognitive decline need to be understood and incorporated into dementia policies and interventions. Culturally grounded approaches, developed with communities, caregivers, and frontline health workers, may help reduce stigma, improve acceptability, and ensure that screening tools and care pathways are locally appropriate [30, 32, 33]. Training challenges, cultural adaptation, and health system preparedness are some of the reasons for the unequal administration of cognitive assessment tests like the Mini-Mental State Examination (MMSE) and the Montreal Cognitive Assessment (MoCA), especially in cases where test content is unavailable in local languages or culturally adapted forms across SSA [30].

Another technological barrier is the limited availability of laboratory infrastructure, neuroimaging, and biomarker assays for diagnosing dementia. Positron emission tomography scans, molecular imaging, and Cerebrospinal Fluid (CSF) biomarker testing are cutting-edge technologies available in high-income countries but are limited in

SSA [25]. Moreover, in SSA, access to MRI imaging, which is essential for dementia diagnosis, is severely constrained by restricted access, high costs, and frequent equipment downtime [26]. Due to limited access to diagnostic technologies, health care practitioners are often left to diagnose based on clinical history alone, making it difficult to distinguish dementia from other causes of intellectual impairment, such as tumors, stroke, and hydrocephalus. This diagnostic uncertainty increases the risk of dementia being underdiagnosed or misdiagnosed. For instance, in rural Kenya and Nigeria, cognitive symptoms can be misattributed to depression, epilepsy, or psychiatric conditions because of the lack of appropriate screening tools and specialized diagnostic equipment [26, 34]. Moreover, Yenesew et al. [35] describe how East African research organizations lack the capacity in biomarker, genetic, and epigenetic research, further limiting region-specific understanding of dementia etiology. This also limits the infrastructure, such as screening tools, culturally adapted interventions, and predictive models of dementia risk. Furthermore, limited coordination among dementia-related data systems may create a cyclical barrier, hindering investment in technology due to insufficient data. Without technology, data cannot be collected and studied properly. Without these basic technologies, SSA countries remain constrained in producing population-level data on dementia subtypes and risk factors. Combined with limited financial resources, structural barriers linked to laboratory infrastructure, biomedical engineering capacity, and maintenance support further restrict the use of modern diagnostic technologies for dementia diagnosis and care. These limit the use of modern technologies in diagnostic equipment for proper dementia care.

The technological gap is also deeply interconnected with human resource shortages. Across SSA, there is a scarcity of individuals with expertise in dementia care, including neurologists, geriatricians, psychiatrists, and neuropsychologists [32, 36, 37]. With a population of 200 million people, Nigeria has only 90 neurologists, substantially constraining the diagnostic capacity of neurological conditions, including dementia [26]. Due to a lack of specialists, mobile and digital screening tools often yield unsatisfactory results, as these tools require confirmatory diagnosis and clinical oversight [38]. Primary healthcare workers often report low confidence in cognitive assessments and in distinguishing dementia from other neurological or psychiatric disorders because of the limited dementia content in medical and nursing curricula. This contributes to diagnostic misclassification, delayed referrals, and the perpetuation of stigmatizing opinions about dementia [33]. The specialist shortage is therefore not simply a workforce issue; it is also a structural technological hindrance that restricts the performance of diagnostic tools, reduces the usefulness of digital screening innovations, and restricts the establishment of multidisciplinary dementia clinics [38]. Access to pharmacological remedies, including acetylcholinesterase inhibitors (donepezil, rivastigmine) and memantine, is minimal due to cost, stock-outs, or absence from

**Fig 3. Technology gaps and limitations affecting dementia care.** Dementia care in SSA is constrained by fragmented and non-standardized screening tools, limited diagnostic technologies, and weak digital health infrastructure, hindering early diagnosis and continuity of care.^1,2^ Structural barriers, including scarce memory clinics, weak referral pathways, and shortages of trained specialists, further limit the effectiveness of technology-enabled interventions.^3^ Persistent system-level constraints affecting telemedicine, mHealth adoption, and dementia data systems remain evident across the region.^4^ **Notes:** ^1^ Limited cultural adaptation of cognitive screening tools [30, 32]. ^2^ Restricted access to neuroimaging and biomarker-based diagnostics [25, 26]. ^3^ Shortages of specialists and memory clinic infrastructure [36, 37]. ^4^ Infrastructure barriers affecting telemedicine and dementia data systems [31, 39].

national formularies [26, 30].

Studies from countries including Ghana, South Africa, and Tanzania reveal similar trends: healthcare providers often lack essential tools to diagnose cognitive changes and functional decline, despite observing these changes [36–38]. Mobile health (mHealth) systems play a crucial role in caregiver education, symptom monitoring, and early disease detection. However, studies indicate that increased dropout rates, usability challenges, and poor integration with existing health care strategies are issues in

SSA [30, 39]. App-based screening tools deployed in Tanzania show potential, but there are some limitations, including the fact that digital screening cannot substitute confirmatory diagnosis, and referral pathways to specialist care remain fragile [38].

Moreover, Paddick et al. demonstrated the feasibility of using a tablet-based dementia screening app that incorporated culturally adapted cognitive assessment items and was administered by non-specialist community health workers. However, they highlighted the limited scope of technology-driven approaches in the absence of broader systemic changes to address issues such as limited specialist referral capacity and insufficient training. Further, without validation, scalable screening technology, and a robust health strategy, early detection will remain unstable. SSA countries cannot fully leverage the benefits of telemedicine or digital caregiver support without national digital health strategies, electronic health records, and stable digital networks. One of the most

pressing challenges is the absence of dedicated, interoperable dementia-specific data systems, despite the presence of broader digital health infrastructures such as electronic health records and DHIS2 platforms in countries including Nigeria, Kenya, and Ethiopia [31, 39]. Without this mechanism, it is challenging for policymakers to make a decision on the prevalence, geographic allocation, and socioeconomic effects of dementia. Figure 3 illustrates the key technology gaps and limitations that affect dementia diagnosis and care delivery in SSA.

## 5 Community-based dementia care and informal caregiving challenges

This subsection discusses community-based dementia care and informal caregiving, which aligns with the WHO’s health system building block of service delivery and its focus on people-centered, community-oriented care. Community-based dementia care and informal caregiving challenges formed the largest thematic group, appearing in 15 of the 33 included studies, with evidence from Kenya, Uganda, Ghana, Benin, Nigeria, South Africa, Tanzania, the Democratic Republic of the Congo, and broader SSA settings (Table 1). Informal care is typically provided in multigenerational households with limited resources and minimal support from the formal health system [40], and caregiving demands increase with increasing severity of behavioral and psychological symptoms of dementia (BPSD) [41]. Studies predominantly reported women as informal caregivers for PLWD, with proportions ranging from 70% to 90% across settings in Uganda, Benin, Tanzania, Ghana, and the Democratic Republic of Congo. Caregivers themselves are often older adults or middle-aged individuals, frequently spouses or adult daughters of care recipients [40, 42–45]. Studies also reveal limited knowledge about dementia, and high levels of unmet support needs among caregivers [40, 46, 47]. While explicit quantification was not available across these studies, the Benin time-use evaluation study estimated that caregivers spend 8-12 hours/day on informal caregiving activities like supervising the PLWD, assisting with activities of daily living (ADL), and managing behavioral symptoms [45].

Studies reported higher caregiving burden across the different country settings.

Three studies specifically reported caregiving burden using a standardized Zarit Burden Interview (ZBI) that assesses subjective caregiver burden and reported moderate to severe burden among informal caregivers of PLWD [42–44]. Studies also reported psychological distress among caregivers with reports of high prevalence of depression, anxiety, and emotional exhaustion. Okwaraji et al. [48] reported a strong association between BPSD severity and burnout and psychological stress among caregivers in Nigeria. They provide the most detailed quantification of caregiver burden, with 40% of caregivers experiencing psychological distress and nearly 25% experiencing emotional exhaustion. Functional impairment and higher BPSD severity were also associated with greater supervision needs and frequent nighttime caregiving requirements [41, 44].

Studies also showed a significant economic burden associated with dementia caregiving in the SSA setting. Caregiving can disrupt employment, as caregivers often struggle to balance their work and caregiving responsibilities. Caregivers often report reducing work hours, leaving jobs entirely, or shifting to informal labor to accommodate caregiving responsibilities [45, 48, 49]. Th´ebaut et al. [45] provided a direct economic quantification of dementia care costs in Benin, estimating the annual dementia care cost in 2021 to be purchasing power parity dollars (PPP$) 2,399. An estimated 92% of this cost was attributed to informal care. This high number underscores the substantial economic burden borne by families when caring for people with learning disabilities. A national situational analysis from South Africa linked dementia-related care

**Fig 4. Community-based dementia care and informal caregiving in SSA.** Evidence indicates that dementia care in SSA is predominantly home and community-based, with informal caregiving largely undertaken by women and characterized by substantial time demands and psychological burden.^1,2^ Limited caregiver training, delayed diagnosis, and weak health-system support further shift

responsibility to households, contributing to economic strain and unmet care needs.^3,4^ **Notes:** ^1^ Gendered informal caregiving roles and caregiver burden [40, 43, 44]. ^2^ Associations between behavioral and psychological symptoms of dementia and caregiver distress [41, 42, 48]. ^3^ Time-intensive caregiving and economic impacts of informal

care [45, 49]. ^4^ Health-system constraints, delayed diagnosis, and limited service availability [28, 49, 50].

expenditures (not just limited to informal caregiving-related expenses) to chronic financial instability [49].

Information on interventions and their effectiveness is limited by study design and lack of generalizability. A study in Uganda found positive associations between interventions such as gardening and improved mental health scores, as well as lower psychological distress among caregivers [51]. A singing training program in South Africa showed associations with improved dementia caregiver confidence [52]. A community intervention focused on stigma reduction demonstrated high rates of acceptance and feasibility, with marked improvements in caregivers’ attitudes towards dementia [53].

Health system resource constraints severely impact PLWD and their caregivers in SSA, both directly and indirectly. Sparse dementia specific services that remain fragmented, underdeveloped, or absent place a heavy burden on informal caregivers [49]. Underdiagnosis or delayed diagnosis of dementia due to a lack of resources creates a cycle of unmanaged symptoms and delayed care with implications for caregivers.

Workforce shortages and gaps, including low provider knowledge, may impact

care-seeking behavior [49, 50]. The responsibility of managing undiagnosed PLWD falls squarely on family members. Informal caregivers themselves receive little to no training on managing dementia symptoms. Unreliable access to medicines, coupled with a lack of information and stigma, may drive families to seek traditional healers, further delaying medical diagnosis and treatment. A lack of financial protection and dependence on out-of-pocket household spending lead families into catastrophic health spending. This financial instability is compounded by the loss of caregiver income [45, 49].

The evidence from SSA highlights substantial caregiver burden, limited or

non-existent caregiver support, and gendered caregiving norms, all of which are further compounded by weakened health system infrastructure. Figure 4 depicts informal caregiving dynamics and locally rooted support systems for dementia care in SSA.

## 6 Financial and economic challenges

Financial and economic barriers remain central determinants of access, quality, and sustainability of dementia care across SSA. Financial and economic challenges were addressed in 5 of the 33 included studies, drawing on SSA, Africa-wide, and global or regional modeling evidence, as summarized in Table 1. Three recent studies included estimates of dementia costs in SSA. Two of these studies were global dementia cost studies that reported estimates of dementia costs in SSA [54, 55]. The remaining

one [56] highlighted results from an earlier study, Guerchet et al. [8]. In Pedroza et al. [54], the methodology relied on a systematic review of the published literature to generate estimates of costs associated with dementia care. These studies mainly

captured costs related to spending on direct health services or monetary costs. Because there are very few cost studies based on primary data published for people living with dementia in SSA to inform modeling, their estimates relied on modeling costs based on covariates and assumptions. The results from the modelling study show that in 2019, for SSA, the spending attributable to dementia was $552 million (Uncertainty interval:

$325-$816 million), 0.7% of total health spending. This estimate was projected to rise to $6,955 million ($3,401 - $12,001 million) by 2050. The second study, Chen et al. [55], reported data on the economic costs (including both monetary and non-monetary costs) of dementia in SSA, based on a macroeconomic model simulation. Specifically, the

authors used a health-augmented macroeconomic model to estimate the macroeconomic burden of Alzheimer’s disease and other related dementias (ADODs). Their model accounted for labor supply effects for the person living with dementia (morbidity and mortality) as well as for their caregivers (reduction in hours of work), as well as age-sex differences, education, work experience, treatment, and formal care costs, diverting from investments and savings. The authors estimate that between 2020 and 2050, the economic burden of ADODs in SSA will be approximately $158 billion (126-199) in international dollars. This translates to 0.108 as a proportion of total economic output (gross domestic product) between 2020 and 2050. Given the rising proportion of the population above the age of 60, these costs could potentially increase rapidly if the appropriate policies and interventions are not implemented to manage them.

While there exists anecdotal and qualitative evidence that the indirect costs, such as informal caregiving, constitute substantial aspects of the economic burden of dementia care for families with people living with dementia, there is limited observed or documented data on these costs. The few existing estimates are modelled approximations from earlier years [56]. It is reported that in 2015, dementia cost SSA $

6.2 billion, of which more than two-thirds (70%) was attributable to informal care provided by the families of people living with dementia. Furthermore, the existing structure of care for people living with dementia in most SSA countries relies predominantly on families providing most of the non-medical support required by people living with dementia. The high proportion of carers signals a potentially enormous cost to national output that is unaccounted for. This is an unaccounted cost because the economies of several SSA countries have significant informal sectors; therefore, such support provided informally is likely under-reported in official records. As such, quantifying non-financial and informal costs will require innovative documentation and analysis.

The existing literature mostly reports modeling costs based on limited data. Hence,

the evidence base has yet to focus on the sources and mechanisms that people living with dementia in SSA use to pay for care. However, there is some evidence on how older adults generally pay for care in SSA. This broader literature base suggests a heavy reliance on household out-of-pocket spending [57]. Additionally, given the general disease burden in SSA countries, we observe that even in countries with national health insurance systems, the benefits plan tends to prioritize infectious diseases over

non-communicable diseases and may not always cover medications. Nonetheless, older populations may be exempt from paying enrollment or premium fees to access benefits under national health insurance programs. Typically, the burden of financing falls on the individual and their family. These include not only direct spending on consultation visits, medication procurement, and diagnostics, but also the bulk of the responsibility for assisting with activities of daily living when necessary, as part of the care plan. In most SSA countries, this responsibility falls predominantly on women within families.

The potential cost drivers in SSA include a rising burden of stroke and other cardiovascular diseases, an aging population, and a population with a relatively lower education level [30]. Understanding the drivers of these financial and economic costs may help shed light on the broader societal implications for the future and highlight potential pathways for addressing, managing, and responding appropriately. While the nascent stage of this research topic means that there are limited studies that primarily explore the determinants of financial and economic costs borne by people living with dementia, there are descriptive suggestions of potential cost drivers in the limited epidemiological studies that do exist. The cost drivers highlighted above suggest that a

**Fig 5. Financial and economic challenges shaping dementia care systems in SSA.** The literature indicates a reliance on modeled cost estimates due to limited primary cost data^1^, a predominant dependence on household out-of-pocket financing^2^, and largely informal, family-based caregiving responsibilities that disproportionately fall on women^3^. **Notes:** ^1^ Synthesized evidence from cost modeling studies in SSA. ^2^ [57]

^3^ [30]

response strategy focused on a life-course and prevention-oriented approach might be appropriate to keep both financial and non-financial costs within manageable limits.

Leveraging the World Health Organization’s six building blocks of a health system framework, the financing principles could identify potential pathways for countries in SSA to comprehensively cover the financial and economic costs of dementia care for people living with dementia and their families. The financing block proposes three functions of a financing system: mobilizing revenue, pooling resources, and purchasing services. Mobilizing revenue refers to the process of raising funds to cover the costs of health services. Pooling addresses the importance of sharing risks across groups by aggregating individuals and resources into groups, enabling them to benefit from economies of scale. Lastly, purchasing refers to the collective mobilization of resources to procure the necessary health goods and services. Countries in SSA vary in their macroeconomic contexts, fiscal space, and potential for revenue mobilization; as such, each country’s approach to developing a comprehensive financing plan for dementia will need to take local constraints into account. Nonetheless, there are global examples of approaches to revenue mobilization, pooling, and purchasing that countries can draw on to develop contextually appropriate responses. For SSA in particular, a key consideration is the relatively young population and the opportunities it presents to develop a financing strategy that prioritizes a prevention-oriented approach to harness gains from a preemptive stance. In terms of specific examples, South Korea and Brazil offer models for pursuing a preventive approach and leveraging a universal health care platform to provide comprehensive coverage for dementia treatment, as well as the formal caregiving support required in later stages through long-term care coverage.

Figure 5 illustrates key financial and economic barriers influencing the organization and sustainability of dementia care systems in SSA.

In summary, the financial and economic costs of dementia in SSA are an area that is underexplored and underrepresented in the current literature. More primary data on costs by severity, age, sex, and caregiver-related factors across the different countries in SSA are needed. While SSA is currently considered a relatively young population, its older population is set to increase substantially by 2050. Understanding the potential financial and economic ramifications fully will be essential for planning appropriately on the continent.

## 7 Discussion

This review highlights the challenges in dementia care in SSA and addresses both social and economic issues. The literature reviewed identifies technology gaps as a significant barrier to early diagnosis, effective monitoring, and coordinated care. Many countries continue to face limited cognitive screening capacity, digital health infrastructure, and trained personnel to deploy telemedicine-based support tools. At the same time, several emerging technologies, including mobile screening applications and community-based digital tools, show promise for improving dementia care in SSA. For example, expanding mobile phone use, growing digital health initiatives, and the increasing prioritization of non-communicable diseases in global health agendas provide a platform for developing and testing cost-effective, sustainable, and innovative approaches to dementia care.

However, challenges related to scalability, limited internet access in rural areas, and insufficient frontline provider training complicate implementation.

Misunderstandings about culture and lack of dementia literacy stop people from getting help and adhering to care pathways, and environment and social factors contribute to inequality. Rural inhabitants have greater difficulty accessing specialized care facilities due to limited service accessibility and insufficient development of the primary care network. Social aspects such as poverty, gender differences, and inadequate

education of the caregiver affect caregiver burden and patient outcomes. This review points out the importance of informal caregivers, mostly women, acting as the primary caregivers without any formal training, respite care, or psychosocial assistance.

In many SSA countries, patients lack insurance coverage and have limited access to government-funded services, including formal care, diagnostic services, and medications. Very few countries have national dementia policies, and these findings emphasize the urgent need for integrated, context-specific solutions that address structural, technological, and social barriers simultaneously. In addition to funding, the results also indicate a set of interrelated weaknesses in the other building blocks of health systems, as defined by WHO, such as service delivery, health workforce capacity, health information systems, and governance, which collectively contribute to the fragmentation and scalability challenges of dementia care in SSA.

A significant strength of this review is its synthesis of the recent literature (2021-2025), which provides an overview of the challenges and technological

opportunities in the care of individuals living with dementia in SSA. Additionally, the thematic synthesis helps capture the various aspects of the care system, caregivers, and technology that have otherwise been treated in isolation. However, the review has several limitations. First, the literature review is based on documented literature, which may not capture the undocumented community practices. Additionally, the heterogeneity of studies across countries in SSA makes direct comparisons difficult.

Finally, the review is based on a narrative synthesis, which did not allow for calculating intervention efficacy. Nonetheless, the findings of the review help guide the development of the literature, policies, and technology in the care of individuals living with dementia in SSA.

## 8 Conclusion

Nevertheless, the current policy interest in aging populations, e-health, and multisector cooperation offers valuable opportunities to improve dementia care and research in SSA. In particular, this scoping review brings together the latest scientific evidence on dementia care across four interconnected areas: environmental and social determinants, technological capabilities, community-based caregiving, and economic barriers. The analysis shows that the issue of dementia care in SSA is shaped by a combination of technological, social, community, and economic inequalities.

Based on the WHO health system framework, this review underscores the role of deficits in health financing, service delivery, human resources, information, and governance in the lack of robust dementia care systems in SSA. Overcoming these deficits is possible through the development of national dementia policy, integration of dementia services in primary healthcare facilities, caregiver training programs, public education, community-based dementia care, affordable digital health innovations, and increased financial protection for people with dementia and their caregivers.

Future studies should focus on using affordable, culturally appropriate, and scalable models of dementia care delivery. For instance, building region-specific dementia data warehouses would help build evidence by collecting harmonized data on dementia prevalence, diagnosis, service delivery, care providers’ needs, cost of care, technology use, and patient outcomes within SSA countries. A dementia data warehouse would assist policymakers in identifying underserved areas, assessing inequalities, evaluating interventions, and allocating resources using locally collected evidence. The following would be necessary for improving dementia care services within the SSA region.

## Supporting information

**S1 File. Evidence extraction matrix of included studies on dementia care gaps in Sub-Saharan Africa.**

## References

1. Prynn JE, Davey C, Davis D, Kuper H, Mugisha J, Seeley J. Cognition in older adults in Uganda: Correlates, trends over time and association with mortality in prospective population study. PLOS global public health. 2023;3(11):e0001798.
2. Brooke J, Ojo O. Contemporary views on dementia as witchcraft in sub-Saharan Africa: A systematic literature review. Journal of clinical nursing.

2020;29(1-2):20-30.

3. Adebisi AT, Salawu MA. Misconception of dementia-related disorders in Sub-Saharan Africa. Frontiers in Neurology. 2023;14:1148076.
4. Mavrodaris A, Powell J, Thorogood M. Prevalences of dementia and cognitive impairment among older people in sub-Saharan Africa: a systematic review. Bulletin of the World Health Organization. 2013;91:773-83.
5. Olayinka OO, Mbuyi NN. Epidemiology of dementia among the elderly in sub-Saharan Africa. International Journal of Alzheimer’s disease. 2014;2014(1):195750.
6. Wakawa IA, Musami UB, Kwairanga SH, Ogualili PN, Mahmood MY, Fugu MA, et al. Management of Dementia in a Resource-Constrained Sub-Saharan African Setting: Outcome of a Retrospective Survey of Clinical Practice in the Only Neuropsychiatric Facility in Northeastern Nigeria. medRxiv. 2024:2024-09.
7. Prince M, Wimo A, Guerchet M, Ali GC, Wu YT, Prina M. World Alzheimer report 2015. The global impact of dementia: an analysis of prevalence, incidence, cost and trends.; 2015.
8. Guerchet M, Mayston R, Lloyd-Sherlock P, Prince M, Akinyemi R, Paddick SM, et al. Dementia in sub-Saharan Africa: Challenges and opportunities. London, UK: Alzheimer’s Disease International; 2017.
9. Nichols E, Steinmetz JD, Vollset SE, Fukutaki K, Chalek J, Abd-Allah F, et al. Estimation of the global prevalence of dementia in 2019 and forecasted prevalence in 2050: an analysis for the Global Burden of Disease Study 2019. The Lancet Public Health. 2022;7(2):e105-25.
10. Akinyemi RO, Owolabi MO, Ihara M, Damasceno A, Ogunniyi A, Dotchin C, et al. Stroke, cerebrovascular diseases and vascular cognitive impairment in Africa. Brain research bulletin. 2019;145:97-108.
11. Organization WH, et al. Global status report on the public health response to dementia. Geneva, Switzerland: World Health Organization; 2021. Available from: <https://www.who.int/publications/i/item/9789240033245>.
12. Apeagyei AE, Lidral-Porter B, Patel N, Solorio J, Tsakalos G, Wang Y, et al. Financing health in sub-Saharan Africa 1990–2050: Donor dependence and expected domestic health spending. PLOS Global Public Health. 2024;4(8):e0003433.
13. Violante F, Collaborators GD, et al. Global age-sex-specific fertility, mortality, healthy life expectancy (HALE), and population estimates in 204 countries and territories, 1950-2019: a comprehensive demographic analysis for the Global Burden of Disease Study 2019. The Lancet. 2020;396(10258):1160-203.
14. Yoseph M, Paddick SM, Gray WK, Andrea D, Barber R, Colgan A, et al. Prevalence estimates of dementia in older adults in rural Kilimanjaro 2009–2010 and 2018–2019: is there evidence of changing prevalence? International Journal of Geriatric Psychiatry. 2021;36(6):950-9.
15. Mushi D, Rongai A, Paddick SM, Dotchin C, Mtuya C, Walker R. Social representation and practices related to dementia in Hai District of Tanzania. BMC Public Health. 2014;14(1):260.
16. Ogunniyi A, Hall K, Baiyewu O, Gureje O, Unverzagt F. Caring for individuals with dementia: the Nigerian experience. West African journal of medicine. 2005;24(3):259-62.
17. Aboderin IA, Beard JR. Older people’s health in sub-Saharan Africa. The Lancet. 2015;385(9968):e9-e11.
18. Amoakoh-Coleman M. Tackling Ghana’s dementia underdiagnosis needs investment in workforce, diagnostics, education and insurance. Nature Aging. 2025:1-1.
19. Trani JF, Moodley J, Maw MTT, Babulal GM. Association of multidimensional poverty with dementia in adults aged 50 years or older in South Africa. JAMA Network Open. 2022;5(3):e224160.
20. Wimo A, Guerchet M, Ali GC, Wu YT, Prina AM, Winblad B, et al. The worldwide costs of dementia 2015 and comparisons with 2010. Alzheimer’s & Dementia. 2017;13(1):1-7.
21. Eze P, Lawani LO, Agu UJ, Acharya Y. Catastrophic health expenditure in sub-Saharan Africa: systematic review and meta-analysis. Bulletin of the World Health Organization. 2022;100(5):337.
22. Indicators AHO. Monitoring the building blocks of health systems. Geneva, Switzerland: WHO Document Production Services. 2010.
23. Doshmangir L, Sanadghol A, Kakemam E, Majdzadeh R. The involvement of non-governmental organisations in achieving health system goals based on the WHO six building blocks: A scoping review on global evidence. PLoS One. 2025;20(1):e0315592.
24. Tricco AC, Lillie E, Zarin W, O’Brien KK, Colquhoun H, Levac D, et al. PRISMA extension for scoping reviews (PRISMA-ScR): checklist and explanation. Annals of internal medicine. 2018;169(7):467-73.
25. Wakawa IA, Musami UB, Kwairanga SH, Ogualili PN, Mahmood MY, Fugu MA, et al. Dementia in a resource-constrained sub-Saharan African setting: A comprehensive retrospective analysis of prevalence, risk factors, and management at the only neuropsychiatric facility in Northeastern Nigeria. Alzheimer’s & Dementia. 2025;21(3):e14538.
26. Kelechi Wisdom E, Soyemi T, Mayowa S, Ede NS, Ubalaeze Solomon E, Iloanusi CA, et al. Building healthcare capacity for neurodegenerative disease management in Nigeria: Challenges and opportunities. Journal of Public Health Research. 2025;14(2):22799036251350957.
27. Ssonko M, Hardy A, Naganathan V, Kalula S, Combrinck M. Dementia subtypes, cognitive decline and survival among older adults attending a memory clinic in Cape Town, South Africa: a retrospective study. BMC geriatrics. 2023;23(1):829.
28. Bosire EN, Blackmon K, Kamau LW, Udeh-Momoh C, Sokhi D, Shah J, et al. Healthcare providers perspectives and perceptions of dementia diagnosis and management at the Aga Khan University Hospital, Nairobi, Kenya. Journal of Alzheimer’s Disease. 2025;104(3):862-74.
29. Beyene DA, Berha AB. Management Practice and Clinical Outcomes of Dementia in Sub-Saharan Africa: A Systematic Review. Behavioural Neurology. 2023;2023(1):2307443.
30. Alemayehu ZG, Ayalew BD, Sime BL, Bonger TD, Zewdie YA, Keraga AS, et al. Dementia in Sub-Saharan Africa: Risk Factors, Public Perception, and Management Approaches. Journal of Medicine, Surgery, and Public Health. 2025:100204.
31. Oyinlola O. Addressing the challenges of dementia care in Nigeria: a call for a comprehensive national strategy. Public Health Challenges. 2024;3(2):e186.
32. Musyimi CW, Muyela LA, Mutiso VN, Mutunga E, Ndetei DM. Understanding dementia care pathways for policy development and service planning in Kenya. Dementia. 2023;22(5):1027-37.
33. Mwendwa P, Mutunga E, Kroll T, De Bru´n A. ‘It is stressful, almost every hour. . . ’: Experiences of caring for people living with dementia in Kenya—An interpretive phenomenological approach. Dementia. 2021;20(8):2916-32.
34. Aballa A, Khakali L, Njoroge W, Kamau LW, Merali Z, Bosire EN. “They are perceived to be witches, and some are killed”: Community perceptions of elderly people living with dementia in Kilifi, Kenya. Journal of Alzheimer’s Disease. 2025;103(2):593-604.
35. Yenesew MA, Krell-Roesch J, Fekadu B, Nigatu D, Endalamaw A, Mekonnen A, et al. Prevalence of Dementia and Cognitive Impairment in East Africa Region: A Scoping Review of Population-Based Studies and Call for Further Research. Journal of Alzheimer’s Disease. 2024;100(4):1121-31.
36. Aryee CL. A Systematic Review of Creating an Ideal Adult Day Center for People with Dementia in Ghana (Sub-Saharan Africa). Cedar Falls, IA, USA: University of Northern Iowa; 2024. Available from: <https://scholarworks.uni.edu/grp/4610>.
37. Mahomed A, Pretorius C. Understanding the lived experiences of family caregivers of individuals with dementia in Soweto, a South African Township. Dementia. 2022;21(7):2264-87.
38. Paddick SM, Yoseph M, Gray WK, Andrea D, Barber R, Colgan A, et al. Effectiveness of app-based cognitive screening for dementia by lay health workers in low resource settings. A validation and feasibility study in rural Tanzania. Journal of Geriatric Psychiatry and Neurology. 2021;34(6):613-21.
39. Mwendwa P, Lawlor B, Kroll T, De Bru´n A. A systematic review and narrative synthesis of the experiences with caring for older people living with dementia in Sub-Saharan Africa. BMC geriatrics. 2022;22(1):961.
40. Ayisi-Boateng NK, Opoku DA, Tawiah P, Owusu-Antwi R, Konadu E, Apenteng GT, et al. Carers’ needs assessment for patients with dementia in Ghana. African Journal of Primary Health Care & Family Medicine. 2022;14(1):3595.
41. Kamoga R, Mubangizi V, Owokuhaisa J, Muwanguzi M, Natakunda S, Rukundo GZ. Behavioral and psychological symptoms of dementia: prevalence, symptom severity, and caregiver distress in South-Western Uganda—a quantitative

cross-sectional study. International Journal of Environmental Research and Public Health. 2023;20(3):2336.

42. Gumikiriza-Onoria JL, Nakigudde J, Mayega RW, Giordani B, Sajatovic M, Mukasa MK, et al. Psychological distress among family caregivers of persons with Alzheimer’s disease and related dementias in Uganda. BMC geriatrics. 2024;24(1):602.
43. Ikanga J, Reyes A, Zhao L, Hill-Jarrett TG, Hammers D, Epenge E, et al. Exploring factors contributing to caregiver burden in family caregivers of congolese adults with suspected dementia. International journal of geriatric psychiatry. 2023;38(9):e6004.
44. Owokuhaisa J, Kamoga R, Musinguzi P, Muwanguzi M, Natukunda S, Mubangizi V, et al. Burden of care and coping strategies among informal caregivers of people with behavioral and psychological symptoms of dementia in rural

south-western Uganda. BMC geriatrics. 2023;23(1):475.

45. Th´ebaut C, Houinato D, Gansou G, Gnonlonfoun D, Adoukonou T, Preux P, et al. Informal care time and costs of dementia care in Benin (West Africa). Neuroepidemiology. 2024.
46. Pokala S, Wandera SO, Kawooya S, Victoria AE, Odo LW, Nakasujja N, et al. Dementia knowledge and caregiver burden among carers of older adults in rural eastern Uganda. Alzheimer’s & Dementia. 2025;21(8):e70587.
47. Musyimi CW, Ndetei DM, Evans-Lacko S, Oliveira D, Mutunga E, Farina N. Perceptions and experiences of dementia and its care in rural Kenya. Dementia. 2021;20(8):2802-19.
48. Okwaraji FE, Onyebueke GC, Nwakaego OE. Burn Out and Psychological Distress among Care Givers of Dementia Patients Attending the Out Patient Geriatric Clinics of Two Nigerian Tertiary Health Institutions. International Neuropsychiatric Disease Journal. 2023;20(4):15-23.
49. Jacobs R, Schneider M, Farina N, du Toit P, Docrat S, Comas-Herrera A, et al. Dementia in South Africa: a situational analysis. Dementia. 2024;23(3):452-75.
50. Walker J, Dotchin C, Breckons M, Fisher E, Lyimo G, Mkenda S, et al. Patient and caregiver experiences of living with dementia in Tanzania. Dementia. 2023;22(8):1900-20.
51. Ainamani HE, Bamwerinde WM, Rukundo GZ, Tumwesigire S, Kalibwani RM, Bikaitwaho EM, et al. Participation in gardening activity and its association with improved mental health among family caregivers of people with dementia in rural Uganda. Preventive medicine reports. 2021;23:101412.
52. Stuart-R¨ohm K, Clark IN, Baker FA. ‘Caregivers’ Experiences of a Singing Training Program to Support Person-Centered Dementia Care. Journal of Music Therapy. 2024;61(3):244-62.
53. Musyimi CW, Muyela LA, Ndetei DM, Evans-Lacko S, Farina N. Acceptability and feasibility of a community dementia stigma reduction program in kenya. Journal of Alzheimer’s Disease. 2024;100(2):699-711.
54. Pedroza P, Miller-Petrie MK, Chen C, Chakrabarti S, Chapin A, Hay S, et al. Global and regional spending on dementia care from 2000–2019 and expected future health spending scenarios from 2020–2050: an economic modelling exercise. EClinicalMedicine. 2022;45.
55. Chen S, Cao Z, Nandi A, Counts N, Jiao L, Prettner K, et al. The global macroeconomic burden of Alzheimer’s disease and other dementias: estimates and projections for 152 countries or territories. The Lancet Global Health. 2024;12(9):e1534-43.
56. Akinyemi RO, Yaria J, Ojagbemi A, Guerchet M, Okubadejo N, Njamnshi AK, et al. Dementia in Africa: current evidence, knowledge gaps, and future directions. Alzheimer’s & Dementia. 2022;18(4):790-809.
57. Reid E, Ghoshal A, Khalil A, Jiang J, Normand C, Brackett A, et al. Out-of-pocket costs near end of life in low-and middle-income countries: a systematic review. PLOS Global Public Health. 2022;2(1):e0000005.

Response to Reviewers

## Response to Editor and Reviewers

Manuscript ID: PGPH-D-26-00901

Manuscript title: *Dementia Care Gaps in Sub-Saharan Africa: A Scoping Review of Technology, Environmental, Community Perspectives, and Financial Constraints*

Dear Reviewers and Editorial Team,

We sincerely thank the Academic Editor and all reviewers for their careful evaluation of our manuscript and for the constructive comments. We have revised the manuscript substantially to improve its methodological transparency, scoping-review framing, citation alignment, data avail­ability, and presentation of included evidence. We have also updated the supplementary file to provide a complete evidence-extraction matrix for the 33 included studies. Below, we provide a point-by-point response to each journal requirement and reviewer comment.

## Response to Journal Requirements

**Comment:** Please confirm whether the wording in the title is correct and add “scoping review” to the manuscript title.

**Response:** Thank you. We have revised the title to clearly identify the article as a scoping review. The revised title is: *Dementia Care Gaps in Sub-Saharan Africa: A Scoping Review of Technology, Environmental and Social Determinants, Community Perspectives, and Financial Constraints*. We will also ensure that the metadata for online submissions exactly matches the revised manuscript title.

**Comment:** Please provide a manuscript source file at revision.

**Response:** We have prepared the revised manuscript source file and will upload it in the required format as part of the revision package.

**Comment:** Please provide separate figure files in .tif or .eps format.

**Response:** Thank you for this instruction. We have prepared the revised figures as separate figure files in the required format. We will upload these files separately during resubmission, in accordance with PLOS figure-file requirements.

**Comment:** Supporting information files were uploaded, but a full list of legends was not included.

**Response:** We have added a Supporting Information legend for the supplementary evidence ex­traction file. The legend now reads: *S1 File. Evidence extraction matrix of included studies on dementia care gaps in Sub-Saharan Africa.* The supplementary file lists all 33 included studies and includes reference number, study author, year, country or region, study design, sample size or data scope, population type, thematic domain, key dementia care gaps or findings, and barrier or gap category.

**Comment:** Potential copyright issues were raised for the map figure and for clip-art or icons in other figures.

**Response:** Thank you for highlighting this important issue. To avoid inconsistency with the PRISMA flow diagram and to remove any uncertainty about the map base layer and map copyright permissions, we removed the previous geographic-distribution map from the revised manuscript. The remaining figures have been renumbered accordingly. For the revised conceptual figures, we have ensured that the graphical elements are either author-created or replaced with elements that are compatible with publication under the PLOS CC BY 4.0 license. We will provide the revised figure files separately with the resubmission.

## Response to General Reviewer Questionnaire Items

**Comment:** Some reviewers indicated that the manuscript only partly met PLOS Global Public Health’s publication criteria or required additional rigor and clarity.

**Response:** We appreciate this assessment and have revised the manuscript to more clearly meet the journal’s expectations. We strengthened the scoping review framing, revised the title and abstract, expanded the Methods section, clarified the search strategy and eligibility criteria, added the study selection process, and included a detailed evidence extraction matrix as S1 File. We also revised the Results sections so that each thematic domain is grounded in the included studies rather than reading only as a narrative essay.

**Comment:** The statistical analysis was questioned by some reviewers.

**Response:** Because this manuscript is a scoping review, we did not conduct a meta-analysis or pooled statistical analysis. We have clarified that the synthesis was descriptive and thematic. We also clarified the screening process and added information on independent screening by reviewers and resolution of disagreements. Since the purpose of the review was to map evidence and iden­tify care gaps, the revised manuscript avoids implying that quantitative effect-size synthesis was performed.

**Comment:** One reviewer indicated that the underlying data were not fully available.

**Response:** Thank you. We have addressed this by providing a complete supplementary evidence extraction matrix as S1 File. The file identifies all 33 included studies and summarizes the extracted information used to support the review findings. Since this is a review of published literature, the underlying evidence consists of publicly available published studies, and the extracted dataset is now provided in the supplementary file for transparency.

**Comment:** Reviewers generally indicated that the manuscript was intelligible and written in standard English, but some language and clarity issues were noted.

**Response:** We revised the manuscript for clarity, precision, and consistency. We reduced overgen-eralized wording, improved alignment between claims and citations, clarified the Methods section,

and revised the abstract, introduction, discussion, and conclusion to improve flow and reduce re­dundancy.

## Response to Reviewer 1

**Comment:** Figure 1 states that 33 studies were included, but the manuscript did not include a complete table of the 33 studies. The reviewer requested a comprehensive data extraction table listing all included studies.

**Response:** Thank you for this important suggestion. We have added a complete supplementary evidence extraction matrix as an S1 File. This file lists all 33 included studies, including the refer­ence number, author, year, country or region, study design, sample size or data scope, population type, thematic domain, key dementia care gaps or findings, and barrier or gap category. This addi­tion allows readers to independently verify the dataset, assess geographic coverage, and understand the types of evidence that informed the thematic synthesis.

**Comment:** The previous map figure contradicted the PRISMA diagram because the map appeared to show broad country-level numbers that did not correspond to the 33 included studies.

**Response:** We agree with this concern. The previous map figure created confusion because the country counts could be interpreted as representing the 33 included studies in the review. To avoid this inconsistency, we removed the map from the revised manuscript. The revised manuscript now relies on the PRISMA flow diagram, Table 1, and S1 File to describe the included evidence. The remaining figures have been renumbered accordingly.

**Comment:** The Methods section did not explain whether screening was independently conducted by two or more reviewers, or how disagreements were resolved.

**Response:** Thank you. We revised the Methods section to clarify the study-selection process. The revised manuscript now states that all records were imported into a reference management file, duplicates were removed, and study selection was conducted in two stages. First, four reviewers independently screened titles and abstracts against the eligibility criteria. Second, full texts were independently assessed by the same reviewers. Disagreements were resolved through discussion and consensus, and a fifth reviewer was available for final decision-making if consensus could not be reached.

**Comment:** Sections 3 through 6 read more like a narrative essay than a scoping review synthesis. The reviewer suggested quantifying findings at the beginning of each thematic section.

**Response:** We agree and have revised the Results sections to better ground the thematic synthesis in the included dataset. We added Table 1, which summarizes the number of studies represented in each thematic domain and the main countries or regions represented. We also added opening sentences to Sections 3 through 6 that report the number of included studies contributing to each domain. Specifically, the revised manuscript states that 5 studies addressed environmental and social challenges, 12 addressed technology-related challenges, 15 addressed community-based de-

mentia care and informal caregiving challenges, and 5 addressed financial and economic challenges. We also clarified that studies could contribute to more than one thematic domain, so the domain totals exceed 33.

**Comment:** The data availability statement was insufficient without a supplementary table detail­ing the 33 publications.

**Response:** Thank you. We have addressed this by providing S1 File, a full evidence extraction matrix of the 33 included studies. This file makes the review dataset transparent and allows readers to verify which publications were included in the synthesis.

**Comment:** The sentence in Section 2.5 stating that the synthesis differs from quantitative syn­thesis was unnecessary.

**Response:** We removed this unnecessary sentence and revised the synthesis section to more directly describe the descriptive and thematic approach used in the scoping review. The revised text now focuses on how extracted data were categorized into health-system capacity gaps, caregiving support gaps, service delivery and access gaps, and technology and innovation gaps.

## Response to Reviewer 2

**Comment:** The reviewer recognized the scholarly value of the manuscript but noted that the manuscript needed stronger methodological transparency, clearer search strategy, better inclusion protocols, and stronger reproducibility safeguards.

**Response:** We sincerely thank the reviewer for this constructive assessment. We have substantially revised the Methods section to improve transparency and reproducibility. The revised manuscript now clearly identifies the study as a scoping review, reports that the review followed PRISMA-ScR guidance, lists the databases searched, specifies the search period from January 2021 to December 2025, and provides the main Boolean search string. We also clarified that Google Scholar, website searches, and citation tracking were used as supplementary sources. In addition, we expanded the eligibility criteria and study selection process, including independent title/abstract and full-text screening by four reviewers and consensus-based resolution of disagreements.

**Comment:** The reviewer noted that some arguments were presented without adequate empirical support and that citations needed stronger alignment with claims.

**Response:** We agree and have revised the manuscript to improve citation alignment. In the Intro­duction, we revised broad statements about dementia facilities and care gaps so that the citations now support the claims. We also replaced or repositioned citations where needed, particularly in the early background section. Throughout the manuscript, we attempted to avoid unsupported generalizations and to connect claims more directly to the included studies or to broader contextual sources.

**Comment:** The reviewer noted redundancy across the abstract, discussion, and conclusion.

**Response:** Thank you. We revised these sections to reduce repetition and improve focus. The abstract now clearly states the scoping review purpose and the four domains. The Discussion now synthesizes the implications of the findings across technology, community care, financing, and health-system capacity. The Conclusion was rewritten to emphasize key implications, including national dementia policies, environmental and social determinants, primary-care integration, care­giver training, affordable digital health solutions, financial protection, and region-specific dementia data infrastructure.

**Comment:** The reviewer noted that the manuscript should be more technically consistent and precise.

**Response:** We revised terminology throughout the manuscript to improve consistency. We now consistently refer to the work as a scoping review, use the four thematic domains throughout the manuscript, and align the domains with the WHO health-system framework. We also added Table 1 and the S1 File to connect the thematic findings to the extracted evidence.

## Response to Reviewer 3

**Comment:** The reviewer recommended major revision and emphasized three main issues: the conflict between Figure 1 and the map figure, the need for a comprehensive table of included studies, and clarification of the narrow temporal scope of the search strategy.

**Response:** We thank the reviewer for these clear recommendations. First, we removed the pre­vious map figure because it conflicted with the PRISMA diagram and could be misinterpreted as showing the distribution of the 33 included studies. Second, we added the S1 File, which provides a comprehensive evidence-extraction matrix for all 33 included studies. Third, we clarified the temporal scope of the review in the Methods section. The revised manuscript now states that the search focused on studies published between January 2021 and December 2025. The five-year window was selected to capture the most recent evidence during a period of growing awareness of population aging and dementia-care needs in SSA, which has led to increased scholarly attention to this topic in recent years. We also clarified in the Introduction that earlier landmark reports and analyses were cited only for epidemiological context and conceptual framing, not as part of the recently included study dataset.

## Response to Reviewer 4

**Comment:** The reviewer expressed concern about the scope used in the study, how it was reflected in the map and data, and how linguistic diversity across SSA was considered during the identification of literature.

**Response:** Thank you for this important point. We have clarified the scope of the review and removed the previous map figure to avoid giving the impression of inaccurate geographic coverage.

The revised manuscript now uses the PRISMA flow diagram, Table 1, and S1 File to show how the included evidence was identified and categorized. We also clarified the temporal scope of the review as January 2021 to December 2025. We acknowledge that SSA is linguistically diverse and that relevant work may appear in English, French, Portuguese, and other languages. We have therefore revised the manuscript to clarify the search scope and acknowledge that the findings are based on studies retrieved through the selected databases and search strategy. This limitation is important because language and indexing differences may affect which SSA evidence is visible in international databases.

## Response to Reviewer 5

**Comment:** The reviewer noted that some in-text citations did not fully support the claims to which they were attached, especially in the Introduction.

**Response:** Thank you for identifying this issue. We reviewed and revised the Introduction to align citations. In particular, we revised the sentence that previously suggested that citations 14–18 directly discussed dementia facilities. The revised text now more accurately states that recent studies from Ethiopia, Ghana, Kenya, Nigeria, South Africa, and Tanzania examined dementia-related care gaps in SSA. We also revised citation 19 to better support the relevant discussion of socioeconomic and financial burdens.

**Comment:** The reviewer suggested that the manuscript should acknowledge the need to under­stand local beliefs about aging and avoid assuming that Western biomedical concepts of dementia can be directly transferred into SSA contexts.

**Response:** We agree fully and have added this point to the technology-related challenges section. The revised manuscript now states that efforts to improve dementia screening and diagnosis in SSA should not assume that dementia screening tools and care pathways can be transferred without local adaptation. We added discussion of local beliefs about aging, memory loss, family responsibility, and what constitutes normal versus pathological cognitive decline. We also emphasized that cultur­ally grounded approaches, developed with communities, caregivers, and frontline health workers, may reduce stigma, improve acceptability, and ensure that screening tools and care pathways are locally appropriate.

## Response to Reviewer 6

**Comment:** The reviewer stated that dementia is becoming a major public health problem in SSA and that the manuscript highlights four critical dementia care gaps.

**Response:** We thank the reviewer for the positive assessment. We have retained the four-domain structure of the manuscript while strengthening the scoping-review framing, methods, evidence table, and discussion of policy implications.

## Response to Reviewer 7

**Comment:** The reviewer noted that a statistical section was not included in the manuscript, although the manuscript was otherwise well written and satisfied publication criteria.

**Response:** Thank you. We clarified that this manuscript is a scoping review and does not conduct a meta-analysis or pooled statistical analysis. Therefore, a conventional statistical analysis section was not appropriate. Instead, we strengthened the Methods section by describing the descriptive and thematic synthesis approach and clarifying the study selection process. We also added Table 1 and S1 File to make the evidence base and thematic categorization transparent.

## Closing Statement

We again thank the Academic Editor and reviewers for their valuable feedback. The revised manuscript has been strengthened by a clearer scoping-review title, expanded methodology, im­proved citation alignment, removal of the inconsistent map figure, addition of Table 1, addition of an S1 File containing all 33 included studies, clarification of the temporal scope, and revisions to the discussion and conclusion. We believe these changes substantially improve the rigor, transparency, and readability of the manuscript.

Sincerely, The Authors
