# Supplementary material for: Dementia care gaps in Sub-Saharan Africa: A scoping review of technology, environmental, community perspectives, and financial constraints
Source: PLOS Glob Public Health. 2026 Oct 6;6(10):e0007265. doi: 10.1371/journal.pgph.0007265 (PMC13641696; doi:10.1371/journal.pgph.0007265)
Supplement: Peer review history

# Peer review: Decision Letter 0

**DOI:** 10.1371/journal.pgph.0007265.r001

**Authors:** Razak Gyasi

12 May 2026

PGPH-D-26-00901

Understanding Dementia Care Gaps in SSA: An Integrated Analysis of Technology, Social Determinants, Community Perspectives, and Financial Constraints

PLOS Global Public Health

Dear Dr. Block Ngaybe,

Thank you for submitting your manuscript to PLOS Global Public Health. After careful consideration, we feel that it has merit but does not fully meet PLOS Global Public Health’s publication criteria as it currently stands. Therefore, we invite you to submit a revised version of the manuscript that addresses the points raised during the review process.

Please submit your revised manuscript by Jul 11 2026 11:59PM. If you will need more time than this to complete your revisions, please reply to this message or contact the journal office at globalpubhealth@plos.org. When you're ready to submit your revision, log on to https://www.editorialmanager.com/pgph/ and select the 'Submissions Needing Revision' folder to locate your manuscript file.

Please include the following items when submitting your revised manuscript:

- A letter that responds to each point raised by the editor and reviewer(s). You should upload this letter as a separate file labeled 'Response to Reviewers'.
- A marked-up copy of your manuscript that highlights changes made to the original version. You should upload this as a separate file labeled 'Revised Manuscript with Track Changes'.
- An unmarked version of your revised paper without tracked changes. You should upload this as a separate file labeled 'Manuscript'.

Guidelines for resubmitting your figure files are available below the reviewer comments at the end of this letter.

As the corresponding author, your ORCID iD is verified in the submission system and will appear in the published article. PLOS supports the use of ORCID, and we encourage all coauthors to register for an ORCID iD and use it as well. Please encourage your coauthors to verify their ORCID iD within the submission system before final acceptance, as unverified ORCID iDs will not appear in the published article. *Only*  the individual author can complete the verification step; PLOS staff *cannot*  verify ORCID iDs on behalf of authors.

We look forward to receiving your revised manuscript.

Kind regards,

Professor Razak Gyasi, PhD, PD

Academic Editor

PLOS Global Public Health

Journal Requirements:

1. During your revisions, please confirm whether the wording in the title is correct and update it in the manuscript file and online submission information if needed. Specifically, Please add ‘scoping review’ to the title of the manuscript.

2. We ask that a manuscript source file is provided at Revision. Please upload your manuscript file as a .doc, .docx, .rtf or .tex.

3. Please provide separate figure files in .tif or .eps format.

For more information about figure files please see our guidelines:  LINK

https://journals.plos.org/globalpublichealth/s/figures

https://journals.plos.org/globalpublichealth/s/figures#loc-file-requirements

4. We have noticed that you have uploaded Supporting Information files, but you have not included a list of legends. Please add a full list of legends for your Supporting Information files after the references list.

5. Some material included in your submission may be copyrighted. According to PLOS’s copyright policy, authors who use figures or other material (e.g., graphics, clipart, maps) from another author or copyright holder must demonstrate or obtain permission to publish this material under the Creative Commons Attribution 4.0 International (CC BY 4.0) License used by PLOS journals. Please closely review the details of PLOS’s copyright requirements here: PLOS Licenses and Copyright. If you need to request permissions from a copyright holder, you may use PLOS's Copyright Content Permission form.

Please respond directly to this email or email the journal office and provide any known details concerning your material's license terms and permissions required for reuse, even if you have not yet obtained copyright permissions or are unsure of your material's copyright compatibility.

Potential Copyright Issues:

a. Figure 2: please (a) provide a direct link to the base layer of the map (i.e., the country or region border shape) and ensure this is also included in the figure legend; and (b) provide a link to the terms of use / license information for the base layer image or shapefile. We cannot publish proprietary or copyrighted maps (e.g. Google Maps, Mapquest) and the terms of use for your map base layer must be compatible with our CC-BY 4.0 license.

Note: if you created the map in a software program like R or ArcGIS, please locate and indicate the source of the basemap shapefile onto which data has been plotted.

If your map was obtained from a copyrighted source please amend the figure so that the base map used is from an openly available source. Alternatively, please provide explicit written permission from the copyright holder granting you the right to publish the material under our CC-BY 4.0 license.

Please note that the following CC BY licenses are compatible with PLOS license: CC BY 4.0, CC BY 2.0 and CC BY 3.0, meanwhile such licenses as CC BY-ND 3.0 and others are not compatible due to additional restrictions.

If you are unsure whether you can use a map or not, please do reach out and we will be able to help you. The following websites are good examples of where you can source open access or public domain maps:

* U.S. Geological Survey (USGS) - All maps are in the public domain. (http://www.usgs.gov)

* PlaniGlobe - All maps are published under a Creative Commons license so please cite “PlaniGlobe, http://www.planiglobe.com, CC BY 2.0” in the image credit after the caption. (http://www.planiglobe.com/?lang=enl)

* Natural Earth - All maps are public domain. (http://www.naturalearthdata.com/about/terms-of-use/)

b. Fig 3-6: Please confirm whether you drew the images / clip-art within the figure panels by hand. If you did not draw the images, please provide (a) a link to the source of the images or icons and their license / terms of use; or (b) written permission from the copyright holder to publish the images or icons under our CC-BY 4.0 license. Alternatively, you may replace the images with open source alternatives. See these open source resources you may use to replace images / clip-art:

- https://commons.wikimedia.org

- https://openclipart.org/

If the reviewer comments include a recommendation to cite specific previously published works, please review and evaluate these publications to determine whether they are relevant and should be cited. There is no requirement to cite these works unless the editor has indicated otherwise.

Additional Editor Comments (if provided):

[Note: HTML markup is below. Please do not edit.]

Reviewers' comments:

Reviewer's Responses to Questions

**Comments to the Author**

1. Does this manuscript meet PLOS Global Public Health’s publication criteria?

Reviewer #1: Partly

Reviewer #2: Partly

Reviewer #3: No

Reviewer #4: Yes

Reviewer #5: Yes

Reviewer #6: Yes

Reviewer #7: Yes

**********

2. Has the statistical analysis been performed appropriately and rigorously?

Reviewer #1: Yes

Reviewer #2: Yes

Reviewer #3: N/A

Reviewer #4: I don't know

Reviewer #5: N/A

Reviewer #6: N/A

Reviewer #7: No

**********

3. Have the authors made all data underlying the findings in their manuscript fully available (please refer to the Data Availability Statement at the start of the manuscript PDF file)?

The PLOS Data policy

Reviewer #1: No

Reviewer #2: Yes

Reviewer #3: Yes

Reviewer #4: Yes

Reviewer #5: Yes

Reviewer #6: Yes

Reviewer #7: Yes

**********

4. Is the manuscript presented in an intelligible fashion and written in standard English?

Reviewer #1: Yes

Reviewer #2: Yes

Reviewer #3: Yes

Reviewer #4: Yes

Reviewer #5: Yes

Reviewer #6: Yes

Reviewer #7: Yes

**********

Reviewer #1: 1. Figure 1 (PRISMA diagram) indicates that 33 studies were included in the final review. However, the manuscript does not contain a summary table of these 33 studies. Without this, the reader cannot independently verify the data, assess the geographical spread, or understand the types of study designs informing your conclusions. I suggest to include a comprehensive Data Extraction Table (either in the main text or as a Supplementary File) that lists all 33 included studies. columns: Author(s), Year, Country of Focus, Study Design, Sample Size (N), Key Objectives, and Main Findings.

2. Figure 2 is captioned: "The numbers refer to the number of included studies from each country [25]." However, the numbers on the map completely contradict your PRISMA diagram. For example, the map shows "383" for South Africa, "31" for Kenya, and "10" for Tanzania. Your PRISMA diagram states that only 33 studies in total were included in this review. It appears this map was pulled directly from Reference 25 (Hassan et al.) without modifying the data to reflect your 33 included studies. Figure 2 must be completely regenerated to accurately map the geographical distribution of the 33 specific studies included in this scoping review.

3. The Methods section (2.4) states: "Two steps are followed in selecting studies... Any doubt about the inclusion of a study is resolved through discussion among the review team." This lacks the rigor expected by PRISMA-ScR guidelines. Please explicitly state whether screening (Title/Abstract and Full-Text) was conducted independently by two or more reviewers. If it was independent, mention how discrepancies were resolved (e.g., consensus or a third tie-breaking reviewer).

4. Sections 3 through 6 read more like a traditional narrative essay than a scoping review synthesis. You highlight specific papers (e.g., Wakawa et al., Ssonko et al., Beyene et al.) in deep detail, but it is unclear how these represent the aggregate of the 33 studies. I Suggest you quantify your findings at the beginning of each thematic section to ground the narrative in your dataset. For example, start the technology section by stating: "Of the 33 included studies, X focused on technological barriers, primarily distributed across Y countries..." before diving into the specific narrative examples. This grounds your thematic analysis in your actual systematic search results.

5. You noted that "All data are publicly available as this is a review of peer-reviewed publications." While true, to comply with PLOS policies, you must provide the aforementioned supplementary table detailing exactly which 33 publications comprise your dataset.

6. In Section 2.5, you state: "This synthesis differs from most data synthesis approaches, which aim to combine data quantitatively." This sentence is unnecessary; it is widely understood that scoping reviews do not require quantitative meta-analysis. I suggest removing this to improve flow.

Reviewer #2: The manuscript demonstrates clear scholarly value, but in its current form it falls short of the rigour, precision, and technical consistency required for publication in a high-impact journal.

At the conceptual level, the article is well-structured and addresses a timely and underexplored issue—dementia care gaps in Sub-Saharan Africa—using a multidimensional lens. The integration of technological, social, community, and financial perspectives reflects a strong systems-thinking approach and aligns appropriately with contemporary global health discourse. The literature base is recent and relevant, and the thematic synthesis shows evidence of critical engagement with existing studies.

However, several areas require substantive refinement before the manuscript can meet acceptable academic and editorial standards. Methodologically, the study lacks sufficient transparency. Key elements such as detailed search strategies, inclusion protocols, and reproducibility safeguards are either underdeveloped or vaguely described. This weakens the credibility of the review process and makes it difficult for readers or reviewers to assess the robustness of the evidence synthesis.

In terms of academic writing quality, the manuscript contains instances of imprecise language, generalisations, and occasional informal expressions that dilute its scholarly tone. Some arguments are presented without adequate empirical backing, while others rely on broad claims that require stronger citation support. There is also noticeable redundancy across sections, particularly between the abstract, discussion, and conclusion, which affects conciseness and clarity.

Reviewer #3: I have uploaded my review as an attachment.

Summary Recommendation:

The manuscript requires Major Revision. The authors must resolve the conflict between Figure 1 and Figure 2, provide a comprehensive table of included studies, and clarify the narrow temporal scope of their search strategy to ensure the "gaps" identified are methodologically grounded.

Reviewer #4: I am concerned about the scope used in the study and how it reflects on the map and the data presented in the study. It shows that the review covered the entire SSA. However, given the linguistic diversity of countries and research being done in the subregion, at the very least, French and English, how did the study factor this in the course of identifying literature for the study? If it was or not, either way, I think it will be important if such contextual clarity is provided.

Reviewer #5: Good work by the authors.

Couple of comments:

1. Please ensure that in text citations support the claims or sentences that they are cited to support. For instance, on page 2, citations 14-18 do not really discuss dementia facilities as the sentence that follows frames them to be. Neither does citation 19. So, please review your work to ensure citations align.

2. You should include an acknowledgement, perhaps in the technology-related challenges or discussion section to discuss the need to understand local beliefs around what constitutes normal aging and how to incorporate that into policies and interventions. Current framing of the article seem to lean toward reification of western conceptualization and absolute medicalization of aging at the expense of local beliefs.

Reviewer #6: Dementia is becoming a major public health problem in Sub-Saharan Africa (SSA). The review highlights four critical dementia care gaps in Sub-Saharan Africa. The summary aims to provide insight and guidance on dementia care gaps in SSA.

Reviewer #7: The statistical section is not included in the manuscript. The manuscript is well written. The manuscript satisfies the publication criteria of PLOS Global Public Health. All necessary data required to support the study are included.

**********

what does this mean?). If published, this will include your full peer review and any attached files.

**Do you want your identity to be public for this peer review?**  If you choose “no”, your identity will remain anonymous but your review may still be made public.

For information about this choice, including consent withdrawal, please see our Privacy Policy

Reviewer #1: No

Reviewer #2: **Yes:**  Prof Ajao Wale G.

Reviewer #3: **Yes:**  Abdelrahim Dafalla Elhag Ahmed

Reviewer #4: **Yes:**  Agbor Ayuk Agbor Junior

Reviewer #5: No

Reviewer #6: **Yes:**  Douglas Bosco Aidoo

Reviewer #7: **Yes:**  Humeda Suekit Ahmed

**********

[NOTE: If reviewer comments were submitted as an attachment file, they will be attached to this email and accessible via the submission site. Please log into your account, locate the manuscript record, and check for the action link "View Attachments". If this link does not appear, there are no attachment files.]

Attachment Submitted filename: REVIEWED PGPH-D-26-00901_reviewer.pdf

Attachment Submitted filename: Reviewer Report Manuscript NumberPGPHD2600901.docx

Attachment Submitted filename: Manuscript Number PGPH-D-26-00901.pdf

Attachment Submitted filename: Reviewer comment.docx

# Author comment: Author response to Decision Letter 1

**DOI:** 10.1371/journal.pgph.0007265.r002

5 Jul 2026

Attachment Submitted filename: Response_to_Reviewers.pdf

# Peer review: Decision Letter 1

**DOI:** 10.1371/journal.pgph.0007265.r003

**Authors:** Yong Zhang

19 Aug 2026

Dementia Care Gaps in Sub-Saharan Africa: A Scoping Review of Technology, Environmental, Community Perspectives, and Financial Constraints

PGPH-D-26-00901R1

Dear Mrs. Block Ngaybe,

We are pleased to inform you that your manuscript 'Dementia Care Gaps in Sub-Saharan Africa: A Scoping Review of Technology, Environmental, Community Perspectives, and Financial Constraints' has been provisionally accepted for publication in PLOS Global Public Health.

Before your manuscript can be formally accepted you will need to complete some formatting changes, which you will receive in a follow up email. A member of our team will be in touch with a set of requests.

Please note that your manuscript will not be scheduled for publication until you have made the required changes, so a swift response is appreciated.

IMPORTANT: The editorial review process is now complete. PLOS will only permit corrections to spelling, formatting or significant scientific errors from this point onwards. Requests for major changes, or any which affect the scientific understanding of your work, will cause delays to the publication date of your manuscript.

If your institution or institutions have a press office, please notify them about your upcoming paper to help maximize its impact. If they'll be preparing press materials, please inform our press team as soon as possible -- no later than 48 hours after receiving the formal acceptance. Your manuscript will remain under strict press embargo until 2 pm Eastern Time on the date of publication. For more information, please contact globalpubhealth@plos.org.

Thank you again for supporting Open Access publishing; we are looking forward to publishing your work in PLOS Global Public Health.

Best regards,

Yong Zhang, Ph.d

Academic Editor

PLOS Global Public Health

***********************************************************

Reviewer Comments (if any, and for reference):

Reviewer's Responses to Questions

**Comments to the Author**

Reviewer #2: All comments have been addressed

Reviewer #6: All comments have been addressed

**********

publication criteria?

Reviewer #2: Yes

Reviewer #6: Yes

**********

3. Has the statistical analysis been performed appropriately and rigorously?

Reviewer #2: Yes

Reviewer #6: (No Response)

**********

4. Have the authors made all data underlying the findings in their manuscript fully available (please refer to the Data Availability Statement at the start of the manuscript PDF file)?

The PLOS Data policy

Reviewer #2: Yes

Reviewer #6: Yes

**********

5. Is the manuscript presented in an intelligible fashion and written in standard English?

Reviewer #2: Yes

Reviewer #6: Yes

**********

Reviewer #2: The manuscript provides a valuable synthesis of recent evidence and offers practical recommendations for policy, research, technology development, caregiver support, and health-system strengthening. I recommend the publication.

Reviewer #6: The author(s) have addressed the issues mentioned earlier. Together, this manuscript is an addition to knowledge

**********

what does this mean?). If published, this will include your full peer review and any attached files.

**Do you want your identity to be public for this peer review?**  If you choose “no”, your identity will remain anonymous but your review may still be made public.

For information about this choice, including consent withdrawal, please see our Privacy Policy

Reviewer #2: **Yes:**  PROF WALE G. AJAO

Reviewer #6: No

**********

Attachment Submitted filename: APPROVED PGPH-D-26-00901_R1_reviewer.docx
